# A Landscape of Pharmacogenomic Interactions in Cancer

**DOI:** 10.1016/j.cell.2016.06.017

**Published:** 2016-07-28

**Authors:** Francesco Iorio, Theo A. Knijnenburg, Daniel J. Vis, Graham R. Bignell, Michael P. Menden, Michael Schubert, Nanne Aben, Emanuel Gonçalves, Syd Barthorpe, Howard Lightfoot, Thomas Cokelaer, Patricia Greninger, Ewald van Dyk, Han Chang, Heshani de Silva, Holger Heyn, Xianming Deng, Regina K. Egan, Qingsong Liu, Tatiana Mironenko, Xeni Mitropoulos, Laura Richardson, Jinhua Wang, Tinghu Zhang, Sebastian Moran, Sergi Sayols, Maryam Soleimani, David Tamborero, Nuria Lopez-Bigas, Petra Ross-Macdonald, Manel Esteller, Nathanael S. Gray, Daniel A. Haber, Michael R. Stratton, Cyril H. Benes, Lodewyk F.A. Wessels, Julio Saez-Rodriguez, Ultan McDermott, Mathew J. Garnett

**Affiliations:** 1 European Molecular Biology Laboratory, European Bioinformatics Institute, Wellcome Genome Campus, Cambridge CB10 1SA, UK; 2 Wellcome Trust Sanger Institute, Wellcome Genome Campus, Cambridge CB10 1SA, UK; 3 Institute for Systems Biology, Seattle, WA 98109, USA; 4 Division of Molecular Carcinogenesis, The Netherlands Cancer Institute, Amsterdam 1066 CX, The Netherlands; 5 Faculty of Medicine, Joint Research Centre for Computational Biomedicine, RWTH Aachen University, Aachen 52057, Germany; 6 Department of EEMCS, Delft University of Technology, Delft 2628 CD, the Netherlands; 7 Center for Cancer Research, Massachusetts General Hospital, Harvard Medical School, Charlestown, MA 02129, USA; 8 Genetically Defined Diseases and Genomics, Bristol-Myers Squibb Research and Development, Hopewell, NJ 08534, USA; 9 Cancer Epigenetics and Biology Program (PEBC), Bellvitge Biomedical Research Institute (IDIBELL), L’Hospitalet 08908, Barcelona, Catalonia, Spain; 10 Department of Cancer Biology, Dana-Farber Cancer Institute, Boston, MA 02215, USA; 11 Department of Biological Chemistry & Molecular Pharmacology, Harvard Medical School, Boston, MA 02215, USA; 12 Research Program on Biomedical Informatics, IMIM Hospital del Mar Medical Research Institute and Universitat Pompeu Fabra, Barcelona 08003, Spain; 13 Institució Catalana de Recerca i Estudis Avançats (ICREA), 08010 Barcelona, Catalonia, Spain; 14 Department of Physiological Sciences II of the School of Medicine, University of Barcelona, L’Hospitalet 08908, Barcelona, Catalonia, Spain; 15 Howard Hughes Medical Institute, Chevy Chase, MD 20815, USA; 16 Cancer Genomics Netherlands, Uppsalalaan 8, Utrecht 3584CT, the Netherlands

## Abstract

Systematic studies of cancer genomes have provided unprecedented insights into the molecular nature of cancer. Using this information to guide the development and application of therapies in the clinic is challenging. Here, we report how cancer-driven alterations identified in 11,289 tumors from 29 tissues (integrating somatic mutations, copy number alterations, DNA methylation, and gene expression) can be mapped onto 1,001 molecularly annotated human cancer cell lines and correlated with sensitivity to 265 drugs. We find that cell lines faithfully recapitulate oncogenic alterations identified in tumors, find that many of these associate with drug sensitivity/resistance, and highlight the importance of tissue lineage in mediating drug response. Logic-based modeling uncovers combinations of alterations that sensitize to drugs, while machine learning demonstrates the relative importance of different data types in predicting drug response. Our analysis and datasets are rich resources to link genotypes with cellular phenotypes and to identify therapeutic options for selected cancer sub-populations.

## Highlights

• We integrate heterogeneous molecular data of 11,289 tumors and 1,001 cell lines
• We measure the response of 1,001 cancer cell lines to 265 anti-cancer drugs
• We uncover numerous oncogenic aberrations that sensitize to an anti-cancer drug
• Our study forms a resource to identify therapeutic options for cancer sub-populations

## Graphical Abstract

**Figure:**
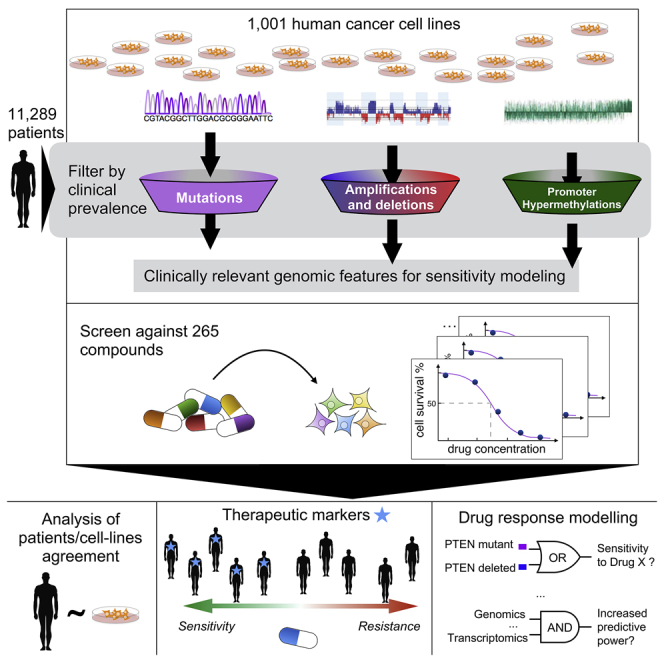

## Teaser

A look at the pharmacogenomic landscape of 1,001 human cancer cell lines points to new treatment applications for hundreds of known anti-cancer drugs.

## Introduction

Cancers arise because of the acquisition of somatic alterations in their genomes that alter the function of key cancer genes (Stratton et al., 2009). A number of these alterations are implicated as determinants of treatment response in the clinic (Chapman et al., 2011, Mok et al., 2009, Shaw et al., 2013). Studies from The Cancer Genome Atlas (TCGA) and the International Cancer Genome Consortium (ICGC) have generated comprehensive catalogs of the cancer genes involved in tumorigenesis across a broad range of cancer types (Lawrence et al., 2014, Tamborero et al., 2013b, Zack et al., 2013). The emerging landscape of oncogenic alterations in cancer points to a hierarchy of likely functional processes and pathways that may guide the future treatment of patients (Ciriello et al., 2013, Hanahan and Weinberg, 2000, Stratton et al., 2009).

Clinical trials are complex and expensive, and pre-clinical data that helps stratify patients can dramatically increase the likelihood of success during clinical development (Cook et al., 2014, Nelson et al., 2015). Thus, pre-clinical biological models that, as much as reasonably possible, capture both the molecular features of cancer and the diversity of therapeutic responses are a necessity. Human cancer cell lines are a facile experimental model and are widely used for drug development. Large-scale drug sensitivity screens in cancer cell lines have been used to identify clinically meaningful gene-drug interactions (Barretina et al., 2012, Basu et al., 2013, Garnett et al., 2012, Seashore-Ludlow et al., 2015). In the past, such screens have labored under the limitation of an imperfect understanding of the landscape of cancer driver genes, but it is now possible to view drug sensitivity in such models through the lens of clinically relevant oncogenic alterations.

**Figure 1:**
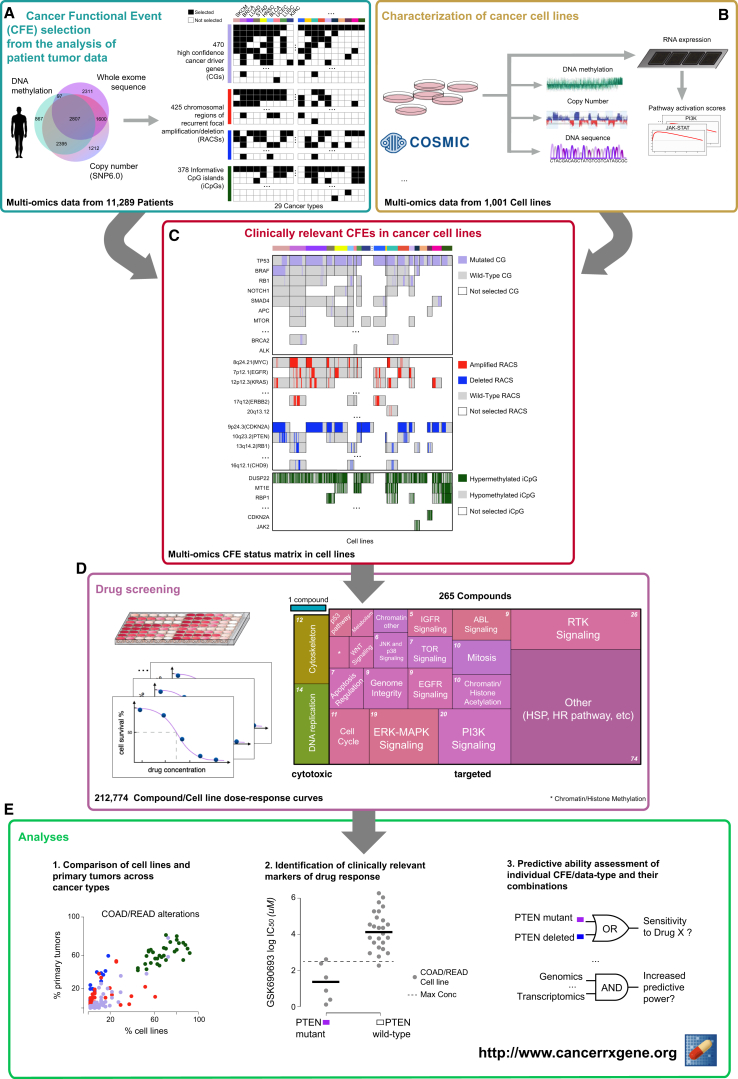
Overview of Data and Analyses (A) Publicly available genomic data for a large cohort of primary tumors were analyzed to identify clinically relevant features called cancer functional events. (B) A panel of 1,001 genomically characterized human cancer cell lines. (C) The catalog of CFEs from patient tumors was used to filter the set of molecular alterations identified in cell lines and subsequently was used for pharmacogenomic modeling. (D) Cancer cell lines were screened for differential sensitivity against 265 anti-cancer compounds. (E) The resultant datasets were used for pharmacogenomic modeling. See also Figure S1 and Table S1.

**Figure S1:**
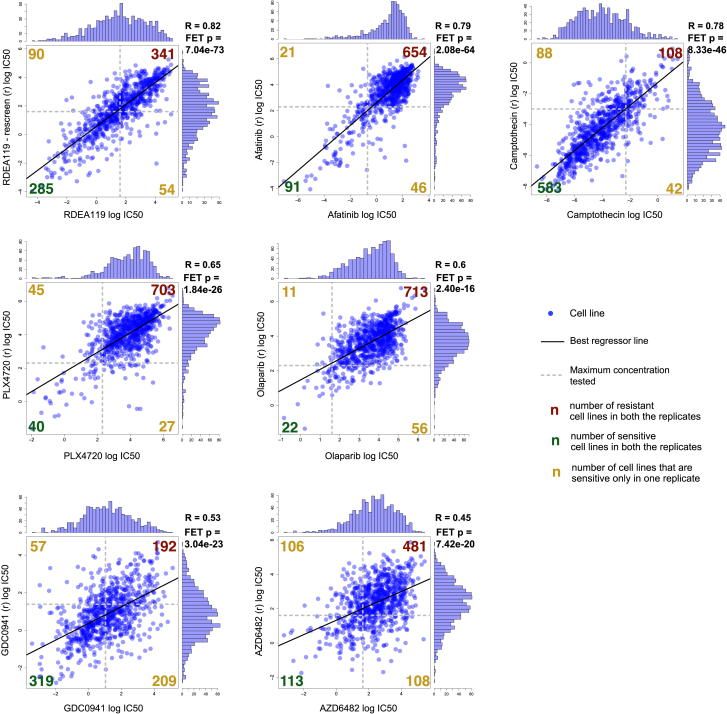
Screened Compound Duplicates, Related to Figure 1 Histograms, scatter plots and Pearson correlation scores between IC_50_ profiles for 7 compounds screened in biological duplicates. In all cases replicate data were generated at least one year apart. Superimposed to each scatter plot is a contingency table (and a corresponding Fisher exact test p-value) showing consistency of sensitive (IC_50_ ≤ maximal tested concentration) and resistant (IC_50_ > maximal tested concentration) cell lines across replicates.

Here, we analyzed somatic mutations, copy number alterations, and hypermethylation across a total of 11,289 tumor samples from 29 tumor types to define a clinically relevant catalog of recurrent mutated cancer genes, focal amplifications/deletions, and methylated gene promoters (Figure 1A; Tables S1A–S1D). These oncogenic alterations were investigated as possible predictors of differential drug sensitivity across 1,001 cancer cell lines (Figures 1B and 1C; Table S1E) screened with 265 anti-cancer compounds (Figures 1D and S1; Table S1F). We have carried out an exploration of these data to determine (1) the extent to which cancer cell lines recapitulate oncogenic alterations in primary tumors, (2) which oncogenic alterations associate with drug sensitivity, (3) whether logic combinations of multiple alterations better explain drug sensitivity, and (4) the relative contribution of different molecular data types, either individually or in combination, in predicting drug response (Figure 1E).

## Results

### Oncogenic Alterations in Human Tumors

We built a comprehensive map of the oncogenic alterations in human tumors using data from TCGA, ICGC, and other studies (Figure 1A; Table S1C). The map consisted of (1) cancer genes (CGs) for which the mutation pattern in whole-exome sequencing (WES) data is consistent with positive selection, 2) focal recurrently aberrant copy number segments (RACSs) from SNP6 array profiles, and 3) hypermethylated informative 5′C-phosphate-G-3′ sites in gene promoters (iCpGs) from DNA methylation data, hereafter collectively referred to as “Cancer functional events” (CFEs). We identified CFEs by combining data across all tumors (pan-cancer), as well as for each cancer type (cancer specific) (Tables S2A, S2D, and S2H).

The WES dataset consisted of somatic variant calls from 48 studies of matched tumor-normal samples, comprising 6,815 samples and spanning 28 cancer types (Tables S1A–S1D). CGs were detected per cancer type by combining the outputs of three algorithms: MutSigCV, OncodriveFM, and OncodriveCLUST (Lawrence et al., 2013, Rubio-Perez et al., 2015, Tamborero et al., 2013a). This identified 461 unique pan-cancer genes (Table S2A). We further added nine genes identified as putative tumor suppressors (Wong et al., 2014). We mined the COSMIC database to identify likely driver mutations in 358 of the 470 CGs (Table S2B; Supplemental Experimental Procedures). Most tumors harbored only a few driver mutations (median n = 2, range 0–64), consistent with previous reports (Kandoth et al., 2013, Vogelstein et al., 2013).

RACSs were identified using ADMIRE for the analysis of 8,239 copy number arrays spanning 27 cancer types (van Dyk et al., 2013) (Table S1D; Supplemental Experimental Procedures). In total, 851 cancer-specific RACSs were gained (286 segments) or lost (565 segments), with a median of 19 RACSs per tumor type (Table S2D). The median number of genes within each RACS was 15 for amplified regions and one for deleted regions. The majority of known driver gene amplifications (e.g., *EGFR*, *ERBB2*, *MET*, and *MYC*) and homozygous deletions (e.g., *CDKN2A*, *PTEN*, and *RB1*) were captured, with 320 RACSs (38%) containing at least one known putative cancer driver gene, in addition to 531 RACSs (62%) without known driver genes. A smaller pan-cancer set (due to overlap in RACSs across cancer types) was constructed by pooling these results, comprising 425 RACSs (117 amplified and 308 deleted) (Tables S2D–S2F).

iCpGs were identified using DNA methylation array data for 6,166 tumor samples spanning 21 cancer types (Table S1D). We defined 378 iCpGs based on a multimodal distribution of their methylation signal in at least one cancer type (Tables S2H and S2I). This also established a discretization threshold used to define such regions as hyper-methylated in the cell lines (Table S2J; Supplemental Experimental Procedures).

**Figure S2:**
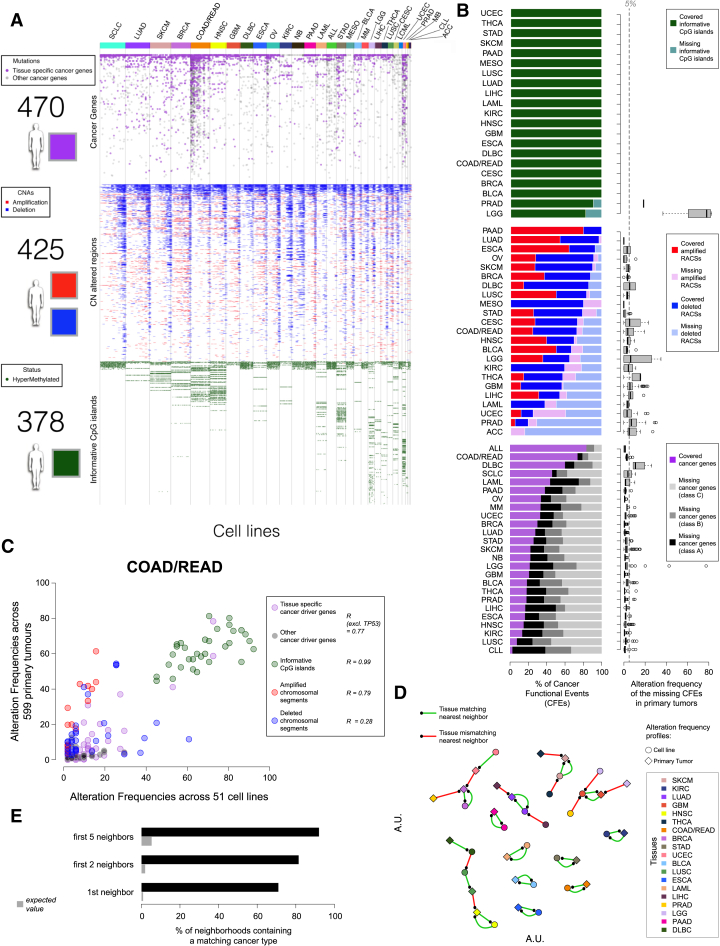
Cancer Functional Events on Cancer Cell Lines, Related to Figure 2 (A) Status of 1,273 Cancer Functional Events (CFEs) identified from primary tumor data in 1,001 cancer cell lines. Each column is a cell line, colors at the top indicate different cancer types, and each row is a CFE. The heatmap is horizontally divided in three parts with (i) high confidence cancer driver genes; (ii) focal recurrently aberrant copy number segments and (iii) informative CpG islands. A white space denotes absence of the functional events, whereas presence is indicated using the color schemes in the adjacent legends. (B) Number of cancer-specific CFEs occurring in at least one cell line from the corresponding tissue, across the three molecular data types. Box plots on the right show the frequency of the missing CFEs in the primary tumors for each cancer type. Percentages of missing cancer genes for each cancer types are grouped based on their confidence (i.e., A = more than two signals of positive selection, B = two signals of positive selection, C = one signal of positive selection). (C) Example of CFE frequency scatter plot for COAD/READ. Each circle is a CFE whose occurrence frequency across cell lines and primary tumors is given by its coordinates, respectively on the x- and y axis. Different CFE types are indicated by color and corresponding correlation scores are reported in the inset. (D) Nearest neighbor analysis for similarities among cell lines and primary tumors based on frequency profiles accounting for all the CFEs. The proximity of two points is proportional to the correlation across the two corresponding CFE frequency profiles. A line connects a point to its closest neighbor (indicated by the small black dot). (E) Performance of a k-nearest-neighbor classifier based on a comprehensive correlation distance between cell lines and primary tumors, accounting for all the CFEs.

In total, our multidimensional analysis of >11,000 patient tumor samples identified 1,699 cancer-specific CFEs, which were further merged into 1,273 unique pan-cancer CFEs (Figure S2A).

#### Oncogenic Alterations in Patient Tumors Are Conserved across Cell Lines

**Figure 2:**
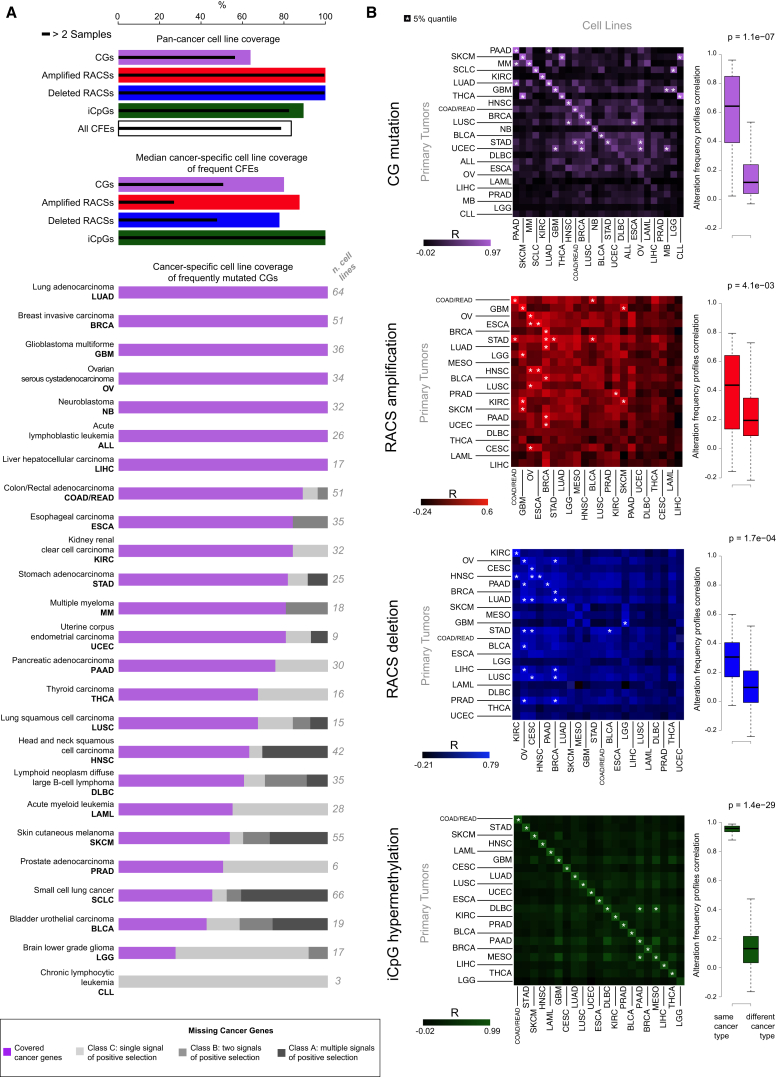
Representation of Cancer Functional Events in Cancer Cell Lines (A) First bar chart: the percentage coverage of cancer functional events (CFEs) in the pan-cancer dataset occurring in at least one cell line. Coverage for each class of CFEs individually and when combined is shown. Second bar chart: the median coverage by cancer type of frequently occurring (>5% of tumor samples) cancer-specific CFEs in at least one cell line. The solid line indicates coverage of CFEs occurring in >2 cell lines. Third bar chart: coverage in each cancer type of frequently occurring cancer genes (CGs). Missing cancer genes are grouped by the level of evidence supporting their classification as a cancer gene. The number of cell lines for each cancer-type and the full name of each cancer-type and associated acronym are shown. (B) Matrix of Pearson correlations of CFE frequency between cell lines and patient tumors for each cancer-type and class of CFEs. Box and whisker plots show the correlations of CFEs within the same (on-diagonal) and between different (off-diagonal) cancer-types. See also Figure S2, Table S2, and Data S1.

Next, we assessed the extent to which the mutational landscape of cancer cell lines captures that seen in primary tumors. We utilized a panel of 1,001 human cancer cell lines analyzed through WES (n = 1,001), copy number (n = 996), gene expression (n = 968), and DNA methylation (n = 957) (http://cancer.sanger.ac.uk/cell_lines) (Figure 1B) and which we reclassified according to the TCGA tissue labels (Figure 2A; Tables S1A and S1E). Molecular alterations identified in cell lines were filtered using the CFEs identified in the primary tumor samples, providing a set of clinically relevant CFEs for the cell lines (Figure 1C).

Of the 1,273 pan-cancer CFEs identified in patient tumors, 1,063 (84%) occurred in at least one cell line, and 1,002 (79%) occurred in at least three (Figure 2A). This concordance was greatest for the RACSs (100% of 425; Table S2G), followed by iCpGs (338 of 378, 89%; Table S2J) and CGs (300 of 470, 64%; Table S2C). When considering cancer-specific CFEs, concordance was highest for CFEs occurring in at least 5% of patients (median of 86% of CFEs covered across cancer types; Figure 2A; Data S1A). Coverage of CFEs varied by cancer type, and when we include infrequent CFEs (occurring in < 5% of patients), this concordance is markedly lower for the majority of cancer types (median coverage = 46%; Figure S2B). CFEs absent in cell lines are reported in Table S2K.

The correlation between the frequency of CFEs in cell lines and patient tumors was high for the majority of the cancer types and for all three classes of CFEs (Figures 2B and S2C; Table S2L; Supplemental Experimental Procedures). Using a simple nearest-neighbor classifier based on the presence of CFEs in cell lines and tumors across cancer types, we could correctly match the tissue of origin of cell lines to primary tumors (and vice versa) for 71% of the cases (27 out of 38 alteration profiles [randomly expected 1%]) (Figures S2D and S2E; Table S2M; Supplemental Experimental Procedures). This percentage increased to 81% and 92% (randomly expected 2% and 5%), when considering the second and fifth nearest-neighbors, respectively (Figure S2E).

**Figure 3:**
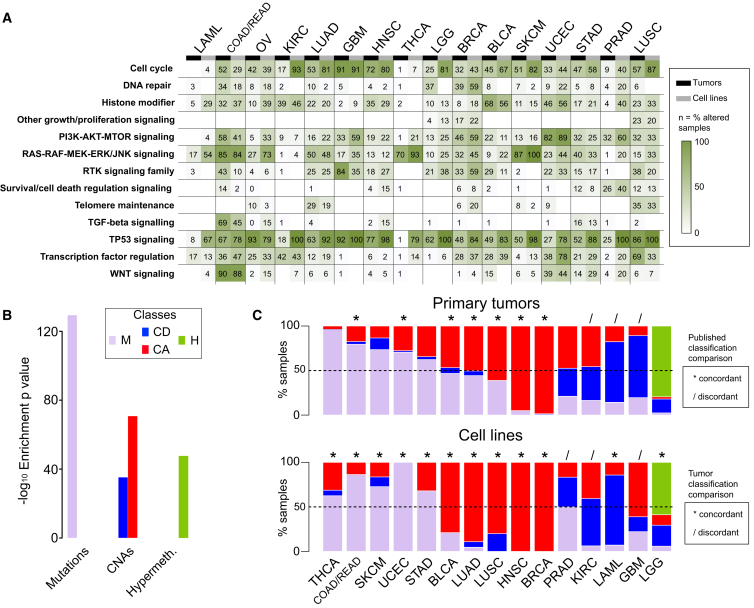
Comparative Analysis of Pathway Alterations and Global CFE Signatures in Cell Lines and Tumors (A) Concordance of CFEs in cancer-associated pathways between cell lines and tumors. (B) Enrichments of the dominant CFE type across four global classes. (C) Classification of primary tumors and cell lines from each cancer type into global classes based on CFEs. Segment lengths are the percentage of samples (cell lines or primary tumors) falling within each global class. For primary tumors, results are compared to published classifications (Ciriello et al., 2013) (top diagram), and for cell lines, the comparison is with primary tumors from the same cancer type (bottom diagram). The classification of concordance is based on the identity of the predominant class of CFEs. See also Figure S3, Table S3, and Data S1.

The frequency of alterations in 13 canonical cancer-associated pathways was highly correlated between cell lines and tumors of the same cancer type (median *R* = 0.75 across all 13 pathways) (Figure 3A; Table S3A).

**Figure S3:**
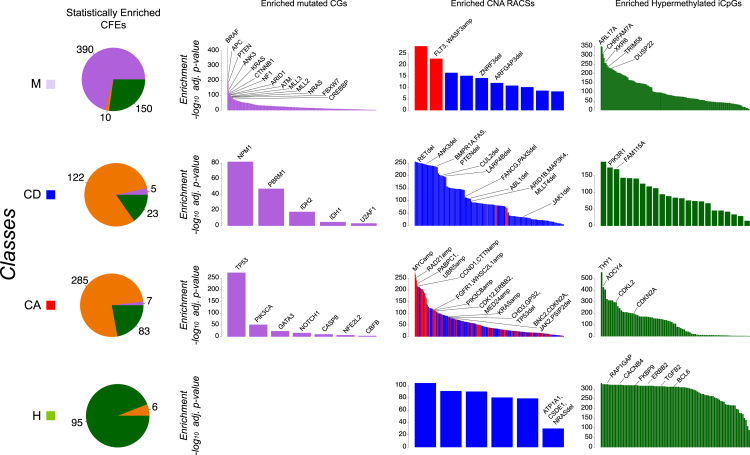
Enrichment of Cancer Functional Events in Global Signatures, Related to Figure 3 Enrichment analysis for global signatures of cancer functional events (CFEs) across different molecular data types identifies 4 classes of CFEs and cell-line/primary-tumor samples (on different rows). Pie charts on the left indicate the proportions of individually enriched CFE data types within each class (orange color indicates generic RACSs, both amplified and deleted). Bar diagrams on the right indicate, for each class and each CFE data type (on different columns), enrichment results for individual cancer functional events. Selected CFEs are highlighted.

A previous hierarchical classification of ∼3,000 tumors identified two major subclasses: M and C class (dominated by mutations and copy number alterations, respectively) (Ciriello et al., 2013). We expanded this analysis by including methylation data and by jointly analyzing cell lines and tumor samples. This integrated analysis of 3,673 samples (composed of 1,001 cell lines and 2,672 primary tumors for which all three data types were available and that were positive for at least one of the 1,250 CFEs [Tables S3B and S3C]) yielded four classes referred to as M, H, CD, and CA (Table S3D; Supplemental Experimental Procedures). Class M is enriched for CG mutations, class H for hypermethylation of iCpGs, and classes CD and CA for deleted and amplified RACSs, respectively (Figures 3B and S3; Tables S3E, S3F, and S3H; Data S1B). We observed a high concordance between the predominant class of CFEs in primary tumors and cell lines of the same tissue type (80% of cancer types, exceptions being GBM, KIRC, and PRAD) (Figure 3C; Table S3G; Data S1B).

Taken together, these results show that a sufficiently large panel of cell lines is able to capture individual clinically relevant genomic alterations, in addition to pathway alterations and global signatures of driver events.

### A Therapeutic Landscape of Human Cancers

#### Modeling Pharmacogenomic Interactions

To investigate how CFEs detected in primary tumors impact drug response, we first mapped these on our panel of cell lines (Figure 1C; Tables S2C, S2G, and S2J). Cell lines underwent extensive drug sensitivity profiling, screening 265 drugs across 990 cancer cell lines and generating 212,774 dose response curves (median number of screened cell lines per drug = 878, range = [366, 935]; Figure 1D). This is an expansion on previous pharmacogenomic datasets (Barretina et al., 2012, Basu et al., 2013, Garnett et al., 2012, Seashore-Ludlow et al., 2015). The effect of each drug on cell number was used to model sensitivity as IC_50_ (drug concentration that reduces viability by 50%) or AUC (area under the dose-response curve) values (Tables S4A and S4B).

Screened compounds included cytotoxics (n = 19) and targeted agents (n = 242) selected against 20 key pathways and cellular processes in cancer biology (Figure 1D; Table S1F). These 265 compounds include clinical drugs (n = 48), drugs currently in clinical development (n = 76), and experimental compounds (n = 141). We screened seven compounds as biological replicates and observed good correlation between replicate IC_50_ values with a median Pearson correlation (R) = 0.65 (0.78 for the compounds with most of IC_50_ values falling within the range of tested concentrations) and consistent classification of cell lines as sensitive or resistant to a compound (median Fisher’s exact test [FET] log_10_ p value = −26) (Figure S1). Cluster analysis based on AUC values confirmed that compounds with overlapping nominal targets or targeting the same process/pathway had similar activity profiles (Table S1G; Supplemental Experimental Procedures).

We used three distinct analytical frameworks to define the contribution of CFEs to the prediction of drug sensitivity (Figure 1E). ANOVA was used to identify single CFEs as markers of drug response. Logic models identified combinations of CFEs that improve the prediction of drug response. Lastly, we used machine-learning algorithms to assess the contribution of each molecular data type (CGs, RACS, iCpGs, and gene expression) in explaining variation in drug response. For consistency, all analyses used IC_50_ values. We carried out a pan-cancer, as well as a cancer-specific, analysis (for those 18 cancer types of sufficient sample size, n > 15 cell lines).

#### ANOVA Analysis Defines a Landscape of Pharmacogenomic Interactions

**Figure 4:**
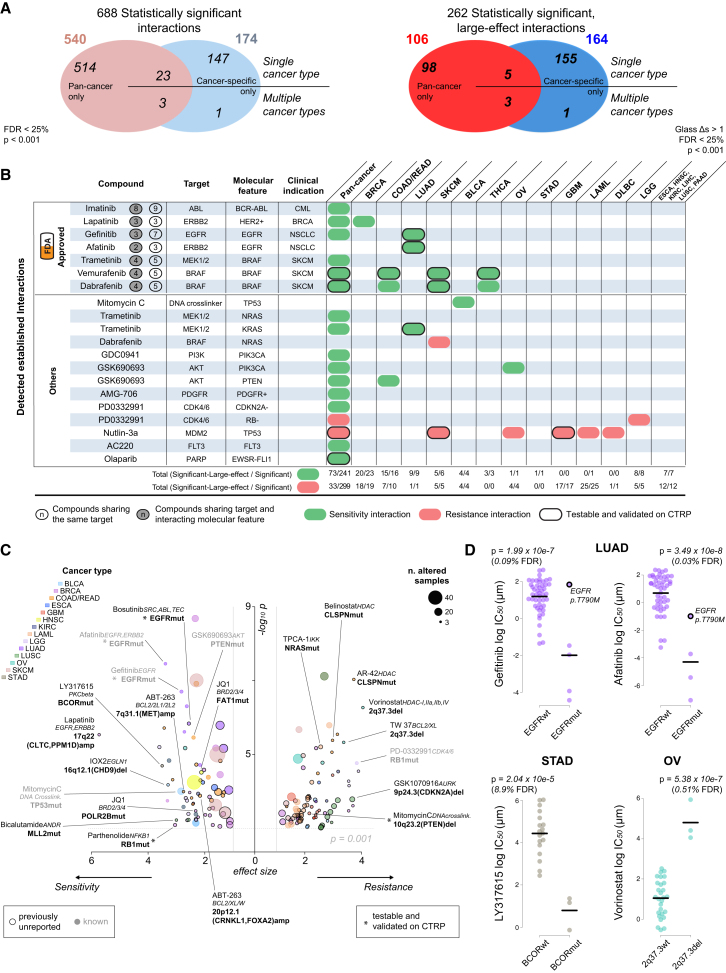
Pharmacogenomic Modeling of Drug Sensitivity (A) Pan-cancer and cancer-specific ANOVA analyses for statistically significant interactions between differential drug sensitivity and CFEs. Cancer-specific interactions are divided into those identified in a single or multiple cancer-specific analyses. (B) A summary of established pharmacogenomic interactions detected in this analysis including a subset of clinically approved markers. The total number of significant and significant large-effect interactions for each cancer type is provided. Testable interactions that were validated on the CTRP datasets are also indicated. (C) Volcano plot with effect size (x axis) and significance (y axis) of large-effect cancer-specific pharmacogenomic interactions. Each circle corresponds to a significant CFE-drug interaction. Circle size is proportional to the number of altered cell lines, and the color indicates cancer type. A subset of interactions is labeled with drug name, target (italics), and name of the associated CFE (bold). (D) Examples of cancer-specific pharmacogenomic interactions identified by our systematic ANOVA. Each circle represents the IC_50_ of an individual cell line. The co-incident resistance-associated EGFR T790M mutation is labeled. See also Figure S4 and Table S4.

**Figure S4:**
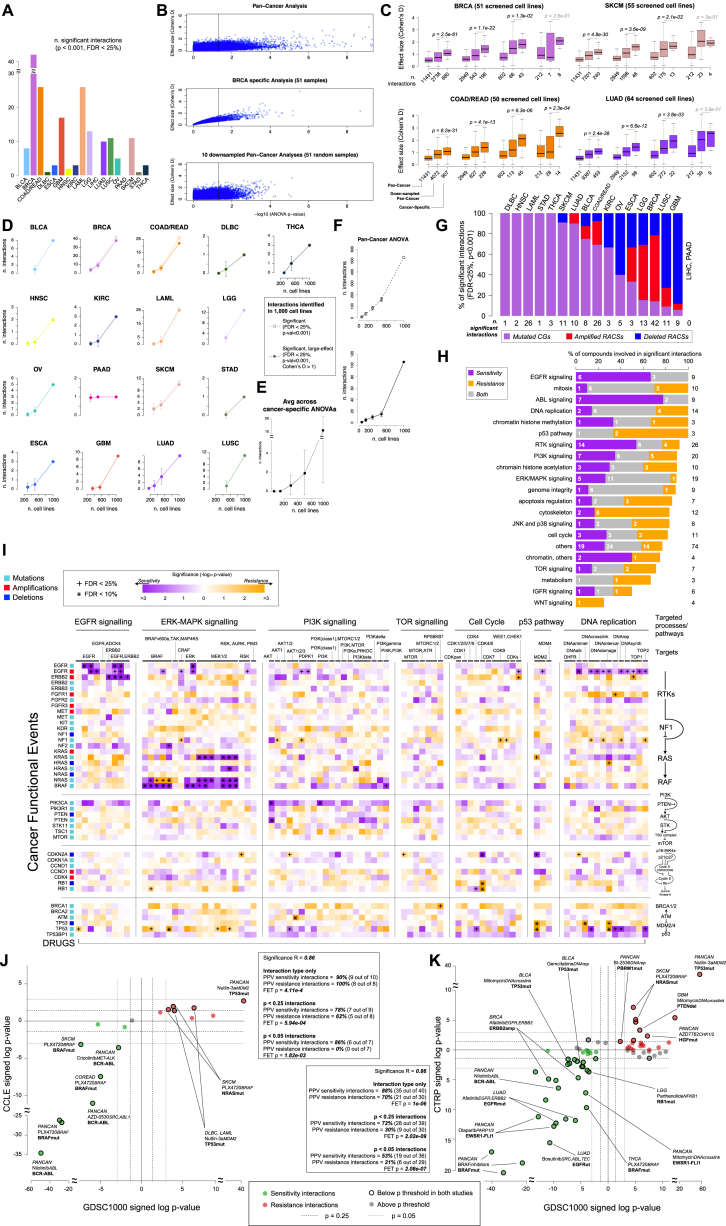
ANOVA Result Summaries, Down-Sampled ANOVA Result Summaries, and ANOVA Validation Using CCLE and CTRP Datasets, Related to Figure 4 (A) The number of statistically significant CFE-drug interactions for each cancer type. (B) Example of ANOVA down-sampling analysis outcomes. Each point is a tested drug-CFE interaction, with position on the x-/y axis indicating significance and effect size, respectively. The vertical line correspond to the significance level p = 0.05. The effect size increment observed in the BRCA specific ANOVA is more evident and less variable than that observed in the down-sampled pan-cancer ANOVA. (C) Effect-size variation for 4 different levels of statistical significance (indicated by the 4 groups of three box-plots) across pan-cancer, down-sampled, and cancer-specific ANOVAs. Each plot refers to a different cancer type (as indicated also by different colors). The effect size increment with respect to the pan-cancer analyses is consistently and significantly greater in the cancer-specific analyses than the down-sampled pan-cancer analyses. The total numbers of significant interactions (and the same value averaged across the sub-sampling simulations) according to the p-value threshold under consideration are reported. (D) Number of significant (dashed lines) and significant large-effect (solid lines) pharmacogenomic interactions identified across 18 cancer-specific ANOVAs (using the whole panel of cell lines) that are retained in simulated down-sampled cancer-specific ANOVAs involving 500, 300, 160 and 60 cell lines. A missing dot means that, for the cancer type under consideration, a cancer-specific analysis is not possible due to reduced sample sizes. (E) Average number of significant large-effect pharmacogenomic interactions identified across 18 cancer-specific ANOVAs (using the whole panel of cell lines) that are identifiable in simulated down-sampled cancer-specific ANOVAs. (F) Number of significant (top plot) and significant large-effect (bottom plot) pan-cancer pharmacogenomic interactions that are identifiable in simulated down-sampled pan-cancer ANOVAs. (G) Proportions of cancer functional event (CFE) types involved in significant pharmacogenomic interactions for each cancer type. (H) Percentage of drugs involved in at least one significant CFE-drug interaction (pan-cancer or cancer-specific) across drugs classified into cancer associated pathways and processes. (I) Pathway-centric overview of the identified pharmacogenomic interactions. Cells are color-coded according to corresponding –log_10_ p-values. Compounds are identified by the nominal therapeutic target. (J) ANOVA results on overlapping GDSC-CCLE datasets. Each circle represents a drug-CFE association. The y axis is the signed log_10_ p-values of the identified interactions on the CCLE and the x axis that on the GDSC. Markers highlighted in red or green are significant in both studies. FET: Fisher exact test of consistency of marker behavior on all or only significant associations. A subset of associations is labeled with cancer-type, drug name, drug target (italics) and associated CFE (bold text). (K) ANOVA results on overlapping GDSC-CTRP datasets.

For pan-cancer ANOVA, the set of CFEs included 267 CGs, 407 RACSs, and three gene fusions (*BCR-ABL*, *EWSR1-FLI1*, and *EWSR1-X*). Overall, for the 265 compounds, we identified 688 statistically significant interactions between unique CFE-drug pairs (p value < 10^−3^ at a false discovery rate [FDR] < 25%; Figure 4A), with 540 pan-cancer and 174 cancer-specific hits (Figure S4A; Table S4C). A subset of 262 CFE-drug pairs was additionally defined as large-effect interactions (Figure 4A). The effect size was quantified through Glass deltas (Δs) and Cohen’s D (CD) (Supplemental Experimental Procedures).

The majority of CFE-drug interactions was exclusively identified in either the pan-cancer or cancer-specific analysis (n = 662 of 688 significant interactions, 96%, and n = 254 of 262 significant large-effect interactions, 97%), with few overlapping interactions (Figure 4A; Table S4C). The effect size was frequently greater for the cancer-specific associations than for pan-cancer associations (CD > 1 for 100% and 30% of hits, respectively) (Table S4D). A possible explanation for this observation could be that cancer-specific associations, with fewer cell lines, require a larger effect size to be statistically significant. However, downsampled pan-cancer analyses confirmed that the increased effect size of cancer-specific associations is greater than expected by downsampling alone (Figures S4B and S4C; Supplemental Experimental Procedures). This indicates that sensitivity to many drugs is modulated by genomic alterations in the context of a defined tissue lineage.

Overall, 233 of 674 (34%) CFEs were significantly associated with the response to at least one compound, and more RACSs (62%) were associated with response than were CGs (38%). The importance of these two classes of CFEs varied by cancer type and was related to their prevalence (Figures 3C and S4G).

We identified significant associations for the majority of compounds (85%; n = 225 of 265). When compounds were classified by their nominal target into 20 specific biological processes (Figure S4H; Table S1F), CFEs best explained sensitivity to compounds targeting EGFR and ABL signaling, mitosis, and DNA replication and least explained sensitivity to compounds targeting TOR, IGF1R, and WNT signaling. For the latter, alternative non-genomic events may be the primary modulators of drug sensitivity. The proportion of cytotoxic and targeted compounds (Table S1F) associated with at least one significant large-effect interaction was similar (63% and 60%, respectively). However, compared to targeted agents, the significant interactions between CFEs and cytotoxics tended to be of a smaller effect size (average CD 0.96 vs. 1.32) and less significant (average –log_10_ p value 3.68 vs. 4.56).

We performed ANOVA on randomly downsampled subsets of cell lines (500, 300, 150, and 60 cell lines) and evaluated our ability to retain the set of statistically significant associations. The number of associations exponentially decreased as the number of cell lines was reduced, with a loss of ∼80% of pan-cancer associations when using 500 cell lines (Figures S4D–S4F; Supplemental Experimental Procedures). This highlights the utility of using a large cell line collection to increase statistical power and to preserve representation of diverse genotypes and histologies.

#### ANOVA Identifies Known and Novel Gene-Drug Associations

Among the individual CFE-drug associations, we identified many well-described pharmacogenomics relationships (Figure 4B). These included clinically relevant associations between alterations in *BRAF*, *ERBB2*, *EGFR*, and the *BCR-ABL* fusion gene and sensitivity to clinically approved drugs in defined tumor types, as well as associations between *KRAS*, *PDGFR*, *PIK3CA*, *PTEN*, *CDKN2A*, *NRAS*, *TP53*, and *FLT3* with drugs that target their respective protein products or pathways (Figure 4B; Table S4C). Moreover, we observed a secondary T790M *EGFR* mutation in lung adenocarcinoma (LUAD) and resistance to EGFR-targeted therapies (Gefitinib and Afatinib) (Godin-Heymann et al., 2008) (Figure 4D), as well as resistance of *NRAS* mutated melanoma patients to a BRAF inhibitor (Figure 4B; Table S4C) (Su et al., 2012).

A pathway-centric view highlighted the number of interactions between CFEs in cancer pathways (EGFR, ERK-MAPK, PI3K-MTOR, and DNA repair and cell-cycle-related pathways) and drugs targeting those CFEs (Figure S4I). For example, compounds targeting EGFR signaling showed potent activity in cells with *EGFR* and *ERBB2* alterations, but were ineffective in cells with downstream alterations in ERK-MAPK signaling, such as mutant *RAS*.

To explore the most important CFE-drug interactions, we focused on 262 associations with a large effect on drug sensitivity (p < 10^−3^, FDR < 25%, and Δ > 1, for both the cell line populations included in the test) (Figure 4C; Table S4C). For example, at the pan-cancer level, *U2AF1* mutations associate with sensitivity to multiple FLT3 inhibitors, such as AC220 (p = 8.3 × 10^−8^, CD = 2.5), Sorafenib (p = 3.04 × 10^−6^, CD = 2.8), Sunitinib (p = 5.6 × 10^−5^, CD = 2.5), and XL-184 (p = 1.3 × 10^−4^, CD = 1.9); *PTEN* mutations associate with sensitivity to an AKT inhibitor in COAD/READ (p = 3.5 × 10^−6^, CD = 2.4). The chemotherapeutic Mitomycin C is widely used to treat BLCA, and here, we detect, in the BLCA specific analysis, a sensitizing interaction with mutations in *TP53* (p = 9.9 × 10^−5^, CD = 2.8) that are highly prevalent in this cancer type. In LUSC cells, loss-of-function mutations in the DNA methyltransferase *MLL2* are associated with sensitivity to the clinical anti-androgen Bicalutamide (p = 6.02 ∼ 10^−4^, CD = 3); the BCL-2 inhibitor, ABT-263, shows activity in COAD/READ cells that harbor focal amplifications of *MET* (p = 1.02 × 10^−4^, CD = 2.8) or *FOXA1/CRNKL1* (p = 1.31 × 10^−4^, CD = 2.2), events found in almost 60% of colorectal tumors; and truncating mutations in the co-repressor of BCL6, *BCOR*, statistically interact (p = 2.04 × 10^−5^, CD = 3.5) with sensitivity to a PKC beta inhibitor in STAD (Figure 4D), and deletions of a RACS (2q37.3) containing *MTERFD2* and *SNED1* is associated with resistance to the HDAC inhibitor Vorinostat (p = 5.4 × 10^−7^, CD = 4; Figure 4D) in OV cell lines.

Interestingly, 24 of the 262 associations are driven by RACSs that do not contain known cancer genes (Tables S4C and S2D). For these regions, the patterns of drug sensitivity may give clues as to the likely contained driver cancer gene(s).

#### Logic Formulas of Drug Response Refine Pharmacogenomic Modeling

**Figure S5:**
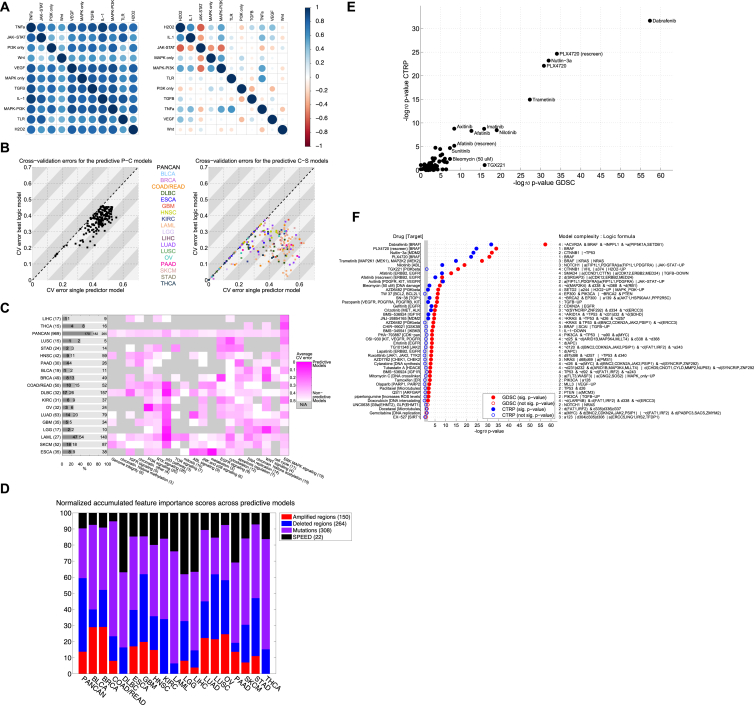
LOBICO Performance and Validation of LOBICO Models on CTRP, Related to Figure 5 (A) Pearson correlation of SPEED pathway activity scores across all cell lines using the original publication cutoffs (left) and our optimized cutoffs (right). (B) Multi-predictor models outperform single predictor models. Scatter plot with the 5-fold cross-validation (CV) error for single predictor models (x axis) and the best (lowest CV error) multi-predictor model (y axis) averaged across 10 repeats for the cancer-specific datasets and 5 repeats for the pan-cancer dataset. Each point represents one of the 390 predictive logic models. The CV errors for the pan-cancer dataset (n = 182) are on the left; the CV errors from the 18 cancer-specific datasets (n = 208) are on the right. (C) CV errors across cancer types and drug classes. Left: Number of drugs for which LOBICO was run, i.e., the drugs with 5 or more sensitive cell lines, number of drugs where a predictive model was inferred, and number of drugs, where the predictive model was a multi-predictor model, for the pan-cancer and each cancer-specific analysis. Center: CV error averaged across all drugs in a drug class (columns) for which LOBICO was run on the pan-cancer or cancer-specific dataset (rows). Grey indicates that no LOBICO models were run for the drugs in a drug class. (D) Feature importance scores across data types: Normalized feature importance (FI) scores for each cancer type grouped into four categories (amplified RACSs; deleted RACSs; mutations in CGs; SPEED pathway activity). These scores were averaged across the drugs for which the LOBICO analysis was performed. (E) t test p-values for LOBICO models on GDSC and CTRP. The scatter plot depicts the −log_10_ p-values for t tests that quantify the difference between cell lines predicted to be sensitive and resistant according to LOBICO. The x axis depicts p-values for the difference between these two groups based on the IC_50_s within GDSC. The y axis depicts p-values for the difference between these two groups based on the AUCs within CTRP. Drugs with a p-value lower than 10^−7^ are annotated. (F) t test p-values on GDSC and CTRP for predictive LOBICO models. The scatter plot depicts the −log_10_ p-values for t tests that quantify the difference between cell lines predicted to be sensitive and resistant according to LOBICO. The 43 drugs are sorted based on the t test p-value derived from the GDSC IC_50_s. P-values are considered significant at p < 0.023 (1/43).

Many genomic alterations occur together or in a mutually exclusive way that suggests a biological function (Babur et al., 2015). We hypothesized that combinations of CFEs could, in some contexts, improve our ability to explain variation in drug sensitivity. We employed a computational approach termed “logic optimization for binary input to continuous output” (LOBICO) to find the optimal logic model combining CFEs to explain the IC_50_ values for a drug, for example, “if *RAS* or *RAF* mutated, then sensitive to MEK inhibition” (Knijnenburg et al., 2016). LOBICO binarizes the IC_50_s, labeling cell lines as sensitive or resistant, and uses these together with the continuous IC_50_s to find optimal models (Table S5C) (Supplemental Experimental Procedures). We employed 5-fold cross-validation (CV) to select the appropriate model complexity from a set of eight possible models, ranging from single CFE predictor models to complex multi-input models with up to four CFEs. We required solutions to have specificity greater than 80%. The input features included the CGs, RACSs, gene fusions, and binarized pathway activity scores derived from the basal gene expression profiles of the cell lines (Figure S5A; Tables S5A, S5B, and S5D). The latter is based on 11 transcriptional signatures of pathway activation (Parikh et al., 2010) (Table S5B; Supplemental Experimental Procedures). LOBICO was executed for each drug separately utilizing pan-cancer and cancer-specific molecular datasets. This led to the inference of 1,112 logic models (Table S5E).

**Figure 5:**
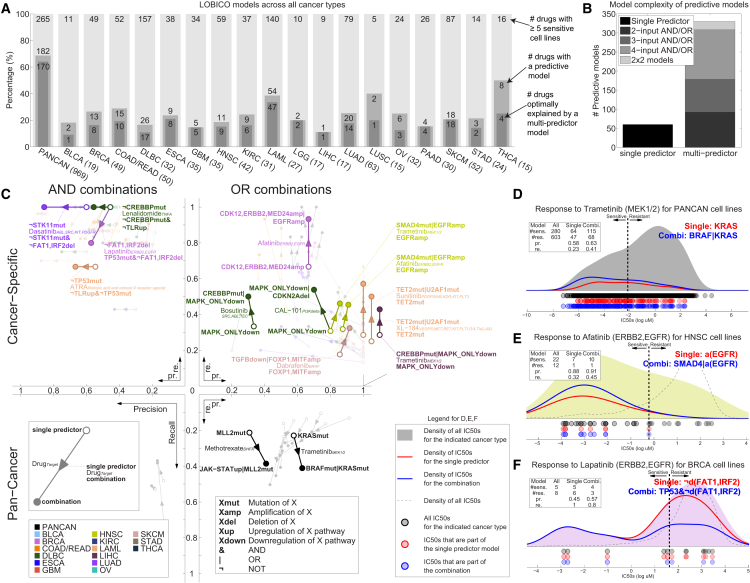
Logic Models of CFEs Explain Drug Sensitivity (A) The number of predictive LOBICO models from the pan-cancer and cancer-specific analyses. The number of cell lines for each cancer type is given in brackets. (B) Optimal model complexity for each of the predictive logic models. (C) Strong AND/OR model combinations involving clinically approved drugs from the pan-cancer and cancer-specific analyses. Each arrow goes from the precision (x axis) and recall (y axis) of the single-predictor model to that of the logic combination. The arrow color reflects cancer type, and drug names and nominal targets (italics) are shown. (D) Distribution of IC_50_ values of all cell lines (gray) in response to Trametinib with respect to the KRAS mutant single-predictor model (red line) and the KRAS OR BRAF mutant combination (blue line). The dashed line is the IC_50_ threshold used to classify cell lines as sensitive and resistant. The inset table shows the number of cell lines classified as sensitive or resistant for each model and the associated precision (pr.) and recall (re.). (E) HNSC cell lines response to Afatinib with respect to EGFR amplification and the combination of EGFR amplification OR a SMAD4 mutation. (F) BRCA cell lines response to Lapatinib with respect to lack of the FAT1/IRF2 deletion and the logical TP53 mutant AND lack of the FAT1/IRF2 deletion combination. See also Figure S5, Table S5, and Data S1.

In the pan-cancer dataset we found that for 69% (182 of 265) of the drugs, the IC_50_s were better explained than expected by chance (p value < 0.05 and FDR < 5%). Across the cancer-specific datasets, on average, 24% of the drugs were explained by the inferred logic models (Figure 5A). We termed these logic models (182 from the pan-cancer dataset and 208 from the 18 cancer-specific datasets) “predictive models”. When considered together, the pan-cancer and cancer-specific LOBICO analyses identified predictive models for 208 out of 265 (78%) drugs. Importantly, for 85% of the 390 predictive models, a multi-input model achieved better performance than did the best single-predictor model (Figure 5B). Although the pan-cancer dataset produced the largest number of predictive models, the CV error was consistently higher than for cancer-specific datasets (Figure S5B). This is in agreement with the ANOVA analysis, where larger effect sizes were observed for the cancer-specific datasets. The response to drugs that target the p53 or ERK-MAPK pathway were especially well-predicted by LOBICO (Figure S5C).

We observed that CGs had the largest role in explaining drug response, followed by RACSs and the pathway activities derived from gene expression (Figure S5A; Supplemental Experimental Procedures). The small number of pathway signatures had a disproportionately large effect in the logic models, showing that basal pathway activation scores provide relevant information to predict drug response beyond the genomic CFEs (Costello et al., 2014) (Figure S5D).

LOBICO uncovered many known, as well as novel, associations (Table S5F). Figure 5C depicts a selection of particularly strong and consistent “and/or” combinations found for clinically approved drugs. For example, in the pan-cancer dataset, the “or” combination of *KRAS* or *BRAF* improved the precision and recall compared to single predictor models to explain cell line sensitivity to a number of MEK and RAF inhibitors (e.g., Trametinib in Figures 5C and 5D).

In general, the “or” combinations led to models with higher recall (Figure 5C, right quadrants) as compared with the single-predictor model. For example, HNSC cell lines that have an *EGFR* amplification or a *SMAD4* mutation account for 45% (10 out of 22) of cell lines sensitive to the ERRB2/EGFR inhibitor Afatinib, whereas considering only the *EGFR* amplified cell lines accounts for only 32% (7 out of 22) of the sensitive cell lines (Figure 5E). Conversely, “and” combinations led to models with higher precision (Figure 5C, left quadrants). For example, BRCA cell lines that lack a deletion of the *FAT1*/*IRF2* locus and are *TP53* mutant show increased sensitivity to the ERRB2/EGFR inhibitor Lapatinib. This is achieved at higher precision (57% instead of 45% for the single predictor model), but at a lower recall (80% instead of 100%) (Figure 5F). Collectively, LOBICO analysis highlights the importance of considering combinations of oncogenic alterations as biomarkers for drug response.

#### Validation of Pharmacogenomic Modeling Results on Independent Datasets

We sought to validate our pharmacogenomic models using independent drug sensitivity datasets from the Cancer Cell Line Encyclopedia (CCLE) (Barretina et al., 2012) and the Cancer Therapeutics Response Portal (CTRP; second version) (Seashore-Ludlow et al., 2015). This analysis was for necessity restricted to only those compounds and cell lines shared with our own study (hereafter referred to as GDSC). The shared set consisted of 466 cell lines and 76 compounds from the CTRP study (Tables S4I–S4K) and 389 cell lines and 15 compounds from the CCLE study (Tables S4E–S4G; Supplemental Experimental Procedures). Validation was performed using IC_50_ values from the GDSC and CCLE studies and AUC values from the CTRP study (where IC_50_ values were not reported).

We performed ANOVA on the overlapping set of cell lines/compounds. We validated 53% (19 of 36 on CTRP) and 86% (6 of 7 on CCLE) of the testable sensitivity associations identified in the GDSC, and 21% (6 of 29 on CTRP) and 0% (0 of 7 on CCLE) of testable resistance associations (p < 0.05, Fisher’s exact test CTRP: p = 8.1 × 10^−9^; CCLE: p = 0.01; Figures S4J and S4K; Tables S4H and S4L; Supplemental Experimental Procedures). A significant Pearson correlation of the CFE-drug interaction significance was observed between the GDSC dataset and the other two datasets (R = 0.86 for CTRP and R = 0.86 for CCLE; Figures S4J and S4K). Similarly, using LOBICO, we validated 44% (17 of 39) of testable models using the CTRP, including both single and multi-input models, and observed a significant Pearson correlation of the interaction significance between the two datasets (R = 0.96; Figures S5E and S5F; Data S1C). Thus, even within the relatively limited set of overlapping drugs and cell lines, resulting in reduced statistical power, we observed reasonable-to-good rates of validation for the set of pharmacogenomic interactions identified in our study, including a number of novel associations. Complete summaries of these comparisons are provided in Tables S4E–S4L and S5G, Data S1C, and Supplemental Experimental Procedures.

#### Contribution of Different Molecular Data Types in Predicting Drug Response

To investigate the power of different combinations of molecular data to predict drug response, we built linear and non-linear models of drug sensitivity (elastic net [EN] regression and Random Forests [Costello et al., 2014]). As input features, we used CGs, RACSs, iCpGs, and gene expression data.

**Figure S6:**
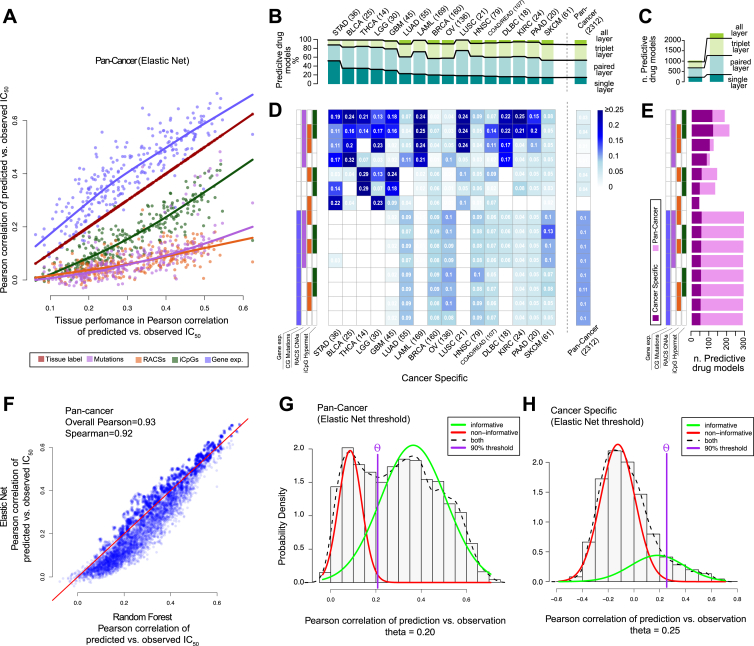
Predictive Ability Assessment of Individual Molecular Feature Layers and Layer Combinations, Related to Figure 6 (A) Predictive performance (Pearson correlation of predicted versus observed IC_50_ values) of tissue label versus other feature layers in pan-cancer analysis with Elastic Net. (B) Percentages of all the predictive models ($R_{\text{pan-cancer}}\geq 0.2$ and $R_{\text{cancer-specific}}\geq 0.25$) across different cancer types and molecular data type. Absolute counts of best performing models are indicated above the bars. (C) Absolute counts of pan-cancer and cancer-specific models separated by number of feature layers. (D) Heatmap split by cancer types and possible feature combination, showing the percentage of all predictive models. (E) Count of all predictive models by data type combination separated in pan-cancer and cancer-specific analysis. (F) Comparison of Random Forests versus Elastic Net performances in the pan-cancer analysis. (G) Deriving pan-cancer threshold of predictive models by fitting a mixed Gaussian distribution across all build models, while assuming that one distribution is informative and the other one is not. A model is considered predictive if the ratio of informative to non-informative is at least 9, resulting in a minimal Pearson correlation of ∼0.2 pan-cancer models achieving high performances due to tissue bias. (H) Deriving cancer-specific threshold in same manner as for pan-cancer, resulting in minimal Pearson correlation of ∼0.25. Negative correlations result from overfitting and too small sample sizes.

Here, we refer to EN models using IC_50_ values (Table S4A), but very similar results were obtained with Random Forests (Figure S6F; Table S6A). We assessed the predictive power of each model using the Pearson correlation coefficient (*R*) of observed versus predicted IC_50_ values. For each of the 265 compounds, we built pan-cancer and cancer-specific models (for 18 cancer types) and considered a model with a corresponding $R_{\text{pan-cancer}}\geq 0.21$ and $R_{\text{cancer-specific}}\geq 0.25$ as predictive (Figures S6G and S6H; Supplemental Experimental Procedures).

**Figure 6:**
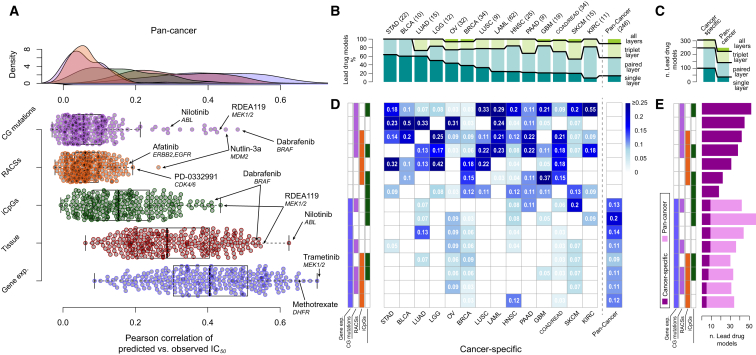
Predictive Ability of Combinations of Molecular Data Types (A) Predictive performances of individual pan-cancer pharmacogenomic models using elastic net modeling and the indicated single data types. Selected outlier predictive models are labeled. (B) The number of molecular data types included in the best-performing models (lead models) across the pan-cancer and cancer-specific analyses. The best-performing models use combinations of multiple data types. Absolute counts of best performing models are given. (C) Absolute counts of lead models from the pan-cancer and cancer-specific analyses and the number of molecular data types used in the models. (D) A heat map of the percentage of lead models identified in the pan-cancer and cancer-specific analyses incorporating different combinations of molecular data types. (E) Absolute count of lead models identified in pan-cancer and cancer-specific analyses incorporating different combinations of molecular data types. Data types are ordered from most (top) to least (bottom) predictive in the cancer-specific analysis. See also Figure S6 and Table S6.

In a pan-cancer analysis, the most predictive data type was gene expression, closely followed by the tissue of origin of the cell lines (Figure 6A). By comparison, genomic features (CG mutations and RACSs alterations) performed poorly. The predictive power of gene expression and tissue type was strongly correlated, while RACSs and CGs are less correlated with the tissue type (Figure S6A). This is consistent with the tissue specificity of gene expression (Ross et al., 2000).

Next, we compared the most predictive data types in pan-cancer versus cancer-specific analyses (Figures 6B and 6C). For each drug, we identified the best-performing combination of data types and the corresponding model, referred to as the “lead model”. Notably, paired molecular data types contributed to the most lead models in both the pan-cancer (∼42% of all models) and the cancer-specific analyses (∼45% for all cancer types) (Figures 4B and 4C). In the pan-cancer analysis, all of the lead models use gene expression data (Figures 6D and 6E), but for 211 drugs (∼86%), the models are improved by including methylation, RACSs, CGs, or any combination of those additional data types. In addition, we identified 379 predictive (non-lead) models (∼17%) independent of gene expression (Figures S6B–S6E).

In a cancer-specific analysis, the majority of lead models are based solely on genomics features (Figures 6D and 6E). For 120 cases (∼38%) the lead model is based on genomics alone (CGs and RACS). We found that genomics in combination with methylation provided an additional 117 lead models (∼37%), whereas genomics in combination with gene expression contributed 19 (∼6%). The remaining lead models use methylation alone (∼7%), gene expression alone (∼3%), or a combination of genomic, epigenetic, and transcriptomic features (12%). Therefore, in the context of a cancer-specific analysis, ∼74% (237 of 319) of lead models were explained by genomics, either alone or when combined with methylation (Figures 6D and 6E).

## Discussion

### Constructing a Pharmacogenomics Resource

Cancer cell lines are important tools for drug development. Here, we have extended previous efforts with the systematic expansion of the pharmacological, genomic, transcriptomic, and epigenetic characterization of 1,001 human cancer cell lines. These datasets can be investigated through the COSMIC and Genomics of Drug Sensitivity in Cancer Web portal (http://www.cancerrxgene.org). To the best of our knowledge, this is the largest and most extensively characterized panel of cancer cell lines and should enable a broad range of studies linking genotypes with cellular phenotypes.

Our analysis of >11,000 patient tumor samples and the subsequent superimposing of salient cancer features on cell lines exemplifies how large-scale cancer sequencing can be used to empower biological research and maximizes the potential clinical relevance of the pharmacological models reported.

The majority of CFEs identified from a broad range of tumor types is captured within a large cell line panel and often at a frequency similar to that observed in patient cohorts. However, the picture is far from complete; many CFEs occurring at low to moderate frequency (2%–5%) are represented by a single cell line or not at all, and coverage by cancer type is variable. As we enter an era of precision cancer medicine, where many drugs are active in small molecularly defined subgroups of patients (e.g., only 3%–7% of lung cancer patients harbor the drug sensitizing *EML4-ALK* gene fusion [Soda et al., 2007]), the scarcity of models for many cancer genotypes and tissues is a limitation. New cell culturing technologies enable derivation of patient cell lines with high efficiency and thus make derivation of a larger set of cell lines encompassing the molecular diversity of cancer a realistic possibility (Liu et al., 2012, Sato et al., 2011).

### Pharmacogenomic Models of Drug Sensitivity

Pharmacogenomic screens in cancer cell lines are an unbiased discovery approach for putative markers of drug sensitivity. We identified a wealth of molecular markers of drug sensitivity, including completely novel associations not easily explained with our current knowledge. With appropriate validation and follow-up studies, these putative biomarkers may aid patient stratification and help to explain the heterogeneity of clinical responses.

Going beyond single gene-drug interactions, “logic” combinations of CFEs consistently perform better than single events in sensitivity prediction. Clinical support for this comes from the observation that *BRAF* mutant melanoma patients treated with BRAF inhibitors show heterogeneity of response that may be explained by the presence of additional molecular alterations (Chapman et al., 2011). Our analyses suggest that clinical studies in cancer patients should be designed to enable combinations of genomic alterations to be detected, which has implications for both trial size and the statistical approaches employed.

We validated our pharmacogenomic models using independent datasets from the CCLE and CTRP. Consistent with previous reports, this demonstrated good consistency in the set of markers identifiable across these studies (Cancer Cell Line Encyclopedia Consortium, 2015) and lends additional support to the results presented here. However, our ability to validate some pharmacogenomic associations was restricted by the limited number of overlapping cell lines and compounds between these studies. Furthermore, the consistency between datasets is not perfect, and efforts toward standardization to reduce methodological and biological differences across the different studies are likely to improve future correlation between datasets.

### Glimpses of a Precision Medicine Landscape

**Figure 7:**
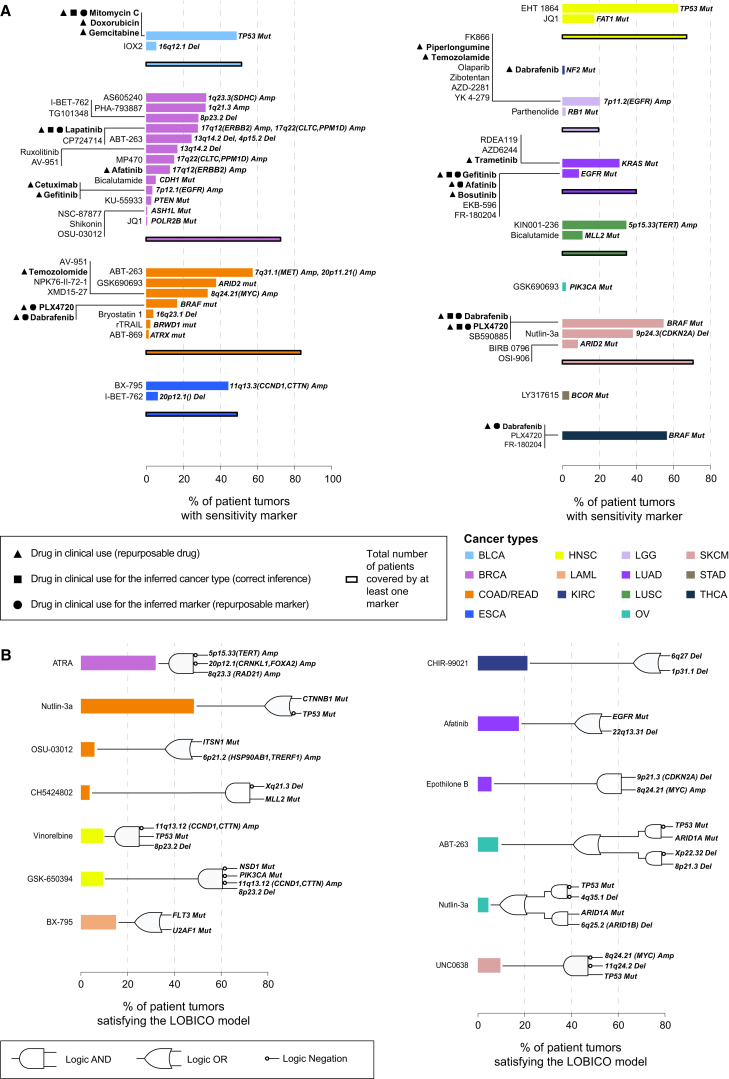
A Precision Medicine Landscape (A) Percentages of primary tumor samples for each cancer type harboring a sensitivity marker to a given compound and the accumulate percentage of patients for all compounds. (B) Percentages of primary tumors whose genomic features satisfy the logic model for sensitivity for a given drug. Corresponding logic circuits are shown to the right of the bars. See also Table S7.

For many of our pharmacological models, the defining CFE is present in clinical populations at a frequency that would make testing in a clinical trial setting feasible (Figure 7). For example, the alkylating agent Temozolamide (used to treat glioblastoma multiforme) shows activity in *MYC* amplified colorectal cancer lines (present in 33% of primary tumors) (Figure 7A). Overall, we found that a median of 50% of primary tumor samples harbor at least one CFE, or logic combination of CFEs, associated with increased drug response; ranging from 0.63% (OV) to 83.61% (COAD/READ) (Figure 7; Tables S7A–S7C; Supplemental Experimental Procedures). This suggests that there are likely to be a number of molecular subtypes within many cancers that, following appropriate validation, could be tested in the clinical trial setting using these stratifications for treatment selection.

Using machine learning, we determined that within each specific cancer type, genomic features (either driver mutations or copy number alterations) generated the most predictive models, with the addition of methylation data further improving our models. While informative in the pan-cancer setting, baseline gene expression data was less informative in the more clinically relevant tissue-specific setting. Prioritizing the design of diagnostics that deliver driver mutations, copy number alterations, and DNA methylation profiles might be the most cost effective means in the short-term to stratify patients for cancer treatment.

### Conclusions

The clinical development of molecularly targeted cancer therapies remains a formidable challenge. Our current analysis is restricted by the availability of patient genomic datasets, the cell lines and compounds screened, and methodological and biological variables, as well as the inherent limitation associated with the use of in vitro cancer cell lines. Nonetheless, our results represent a comprehensive attempt to describe the landscape of clinically relevant pharmacogenomics interactions in cellular models of cancer, complementing previous efforts (Barretina et al., 2012, Basu et al., 2013, Garnett et al., 2012, Seashore-Ludlow et al., 2015). The data resource and analyses described here should enable the matching of drug response with oncogenic alterations to provide insights into cancer biology and to accelerate the development of patient stratification strategies for clinical trial design.

## Experimental Procedures

### Cancer Cell Line Characterization

Genomic data for a panel of 1,025 genetically unique human cell lines were assembled from the COSMIC database. 1,001 cell lines were included in this study (Table S1E). Variants and copy number alterations were identified as described in the Supplemental Experimental Procedures. Microsatellite instability data were assembled as detailed in the Supplemental Experimental Procedures. Gene fusions from a subset cell lines (∼700) were identified by targeted PCR sequencing or split probe fluorescence in situ hybridization (FISH) analysis (Table S2C).

### Variant Identification in Tumors

Variant data from sequencing of 6,815 tumor normal sample pairs derived from 48 different sequencing studies were compiled (Rubio-Perez et al., 2015). To aid in the analysis, the tumor data were reannotated using a pipeline consistent with the COSMIC database (Vagrent: https://zenodo.org/record/16732#.VbeVY2RViko).

### Methylation Data

For primary tumors, raw data for 6,035 methylation samples, covering 18 tumor types, were downloaded from the TCGA data portal. For the cell lines, data were generated in-house as described in the Supplemental Experimental Procedures. In both cases, Infinium HumanMethylation450 BeadChip arrays were preprocessed using the R Bioconductor package Minfi. Only CpG site probes falling on the promoter region of the known genes were considered, i.e., TSS1500, TSS200, 5′ UTR, and 1st exon. Probes containing SNPs and non-specific probes, falling on sex chromosomes, and not associated with a gene were discarded. Methylation beta values of CpG islands were averaged across CpG sites.

### Identification of Cancer Functional Events

The selection of cancer-driver genes (together with the variant recurrence filter) of the recurrently copy-number-altered chromosomal regions and the informative CpG islands is detailed in the Supplemental Experimental Procedures.

### Gene Expression Data

Cell line pellets collected during exponential growth in RPMI or DMEM/F12 were lysed with TRIzol (Life Technologies) and stored at −70°C. Following chloroform extraction, total RNA was isolated using the RNeasy Mini Kit (QIAGEN). DNase digestion was followed by the RNAClean Kit (Agencourt Bioscience). RNA integrity was confirmed on a Bioanalyzer 2100 (Agilent Technologies) prior to labeling using 3′ IVT Express (Affymetrix). Microarray analysis was performed as described in the Supplemental Experimental Procedures.

### Cell Line versus Tumor Comparisons

All analyses evaluating the extent to which cell lines resemble primary tumors are detailed in the Supplemental Experimental Procedures.

### Cell Viability Assays

Experimental protocols used for compound screening are detailed in the Supplemental Experimental Procedures. Effects on cell viability were measured, and a curve-fitting algorithm was applied to this raw dataset to derive a multiparameter description of the drug response (half maximal inhibitory concentration (IC_50_),and area under the curve [AUC]) through a multilevel mixed model (Vis et al., 2016) (Supplemental Experimental Procedures).

### Statistical Models of Drug Response

For each drug an ANOVA model was fitted to correlate drug response with the status of Cancer Functional Events (CFEs), as described in Garnett et al. (2012), implemented in GDSCtools (http//gdsctools.readthedocs.io) and detailed in the the Supplemental Experimental Procedures. The downsampling ANOVA simulation studies are detailed in the Supplemental Experimental Procedures. We applied the LOBICO (Knijnenburg et al., 2016) framework as detailed in the Supplemental Experimental Procedures. Machine learning models were computed as detailed in the Supplemental Experimental Procedures.

## Author Contributions

Conceptualization, F.I., T.A.K., D.J.V., G.R.B., M.P.M., M.Sc., L.F.A.W., J.S.-R., U.M., and M.J.G.; Methodology, F.I., T.A.K., D.J.V., G.R.B., M.P.M., M.Sc., S.B., U.M., and M.J.G.; Software, F.I., T.A.K., D.J.V., M.P.M., M.Sc., T.C., H.L., and E.v.D.; Validation, F.I., T.A.K., D.J.V., M.P.M., N.A., S.B., H.L., P.G., and M.J.G.; Formal Analysis, F.I., T.A.K., D.J.V., M.P.M., and M.Sc.; Investigation, G.R.B., S.B., P.G., T.M., and L.R.; Resources, D.J.V., G.R.B., M.Sc., E.G., S.B., H.L., P.G., E.v.D., H.C., H.d.S., H.H., T.M., S.M., L.R., X.D., R.K.E., Q.L., X.M., J.W., T.Z., N.S.G., S.S., D.T., N.L.-B., P.R.-M., M.E., D.A.H., C.H.B., U.M., and M.J.G.; Data Curation, F.I., D.J.V., G.R.B., M.Sc., E.G., H.L., P.G., H.C., H.d.S., H.H., S.M., S.S., M.So., D.T., N.L.B., P.R.-M., L.F.A.W., J.S.-R., U.M., and M.J.G.; Writing – Original Draft, F.I., T.A.K., D.J.V., G.R.B., M.P.M., U.M., and M.J.G.; Writing – Review & Editing, F.I., T.A.K., D.J.V., G.R.B., M.P.M., M.Sc., N.A., L.F.A.W., J.S.-R., U.M., and M.J.G.; Visualization, F.I., T.A.K., M.P.M., M.Sc., and E.G.; Supervision, D.A.H., M.R.S., C.H.B., L.F.A.W., J.S.-R., U.M., and M.J.G.; Project Administration, F.I., U.M., and M.J.G.; Funding Acquisition, D.A.H., C.H.B., M.R.S., L.F.A.W., J.S.-R., U.M., and M.J.G.

## Accession Numbers

The accession numbers for the sequencing/copy number, transcriptional, and methylation data reported in this paper are, respectively, EGA: EGAS00001000978, GEO: GSE68379, and ArrayExpress: E-MTAB-3610.

## Supplemental Information

Document S1. Supplemental Experimental Procedures and Data S1

Table S1. Lists of Cancer Types, Samples, Studies, Cell Lines, and Screened Compounds, Related to Figure 1 (A) Cancer types included in the study, with definition of corresponding TCGA labels, indicating the tissue of origin of the samples. (B) Number of samples included in the studies, across cancer types and sample types (cell line or tumor), and their level of characterization. (C) Studies from which the patient tumor data was derived. (D) Complete list of tumor samples, with corresponding studies, molecular data availability, information about hypermutator and microsatellite instability status, and TCGA identifier mapping across molecular data types. (E) Annotated complete list of cell lines included in the study with annotations, screening setting information and molecular/drug-response data availability. (F) List of screened compounds, with corresponding targets, targeted process/pathways and additional information. (G) Compound clustering based on AUC pattern similarity.

Table S2. Lists of Cancer Functional Events and Characterization of Patient Tumors and Cell Lines, Related to Figure 2 (A) List of cancer driver genes predicted by the intOGen pipeline or from Wong et al., 2014. (B) Full list of genomic variants found in tumors. (C) Full list of genomic variants found in cell line. (D) Pan-cancer and cancer-specific region of focal amplification/deletion (RACSs). (E) Pan-Cancer/Cancer-Specific RACSs mapping. (F) Full list of RACSs copy number alterations (CNAs) found in primary tumors. (G) Full list RACSs CNAs found in cell lines. (H) Full list of informative CpG islands with hyperlinks to webpages containing corresponding statistical scores. (I) Full list of informative CpG islands found hypermethylated in primary tumors. (J) Full list of informative CpG islands found hypermethylated in cell lines. (K) Cancer Functional Events (CFEs) identified in primary tumor and not occurring in cell lines. (L) Pair-wise correlation scores among CFE alteration frequency profiles, across cell lines and tumors, with hyperlinks to web-pages containing scatter- and anchored-plots. (M) Global correlation matrix resulting from scaling and averaging the pair-wise correlation matrices computed across all tumors and cell lines (used as input to the k-nearest-neighbor classifier).

Table S3. Comparison of Cell Lines and Primary Tumors at the Level of Pathway Alterations and Oncogenic Signatures, Related to Figure 3 (A) Summary of 13 different pathway alterations across cancer types and comparison between cell lines and tumors. Includes hyperlinks to scatter plots and statistical scores. (B) Binary matrix summarizing the status of the Cancer Functional Events (CFEs) across cell lines and tumors. Only fully characterised tumor samples have been included and only CFEs occurring in at least one sample, and samples with at least one occurring CFE have been included. (C) Cell lines and Tumors binary matrix modeled as bipartite network (edge list, .sif format). (D) Identified classes of CFEs and cell-line/tumor samples. (E) CFE type enrichments of the identified classes and sample type compositions. (F) Individual CFE enrichments of the identified classes. (G) Cancer type classification across the identified classes and classification concordance analysis. (H) Copy number amplifications/deletions enrichments of the identified classes.

Table S4. Drug Response Data, ANOVA Results, and Validation on Independent Datasets, Related to Figure 4 (A) IC*50* values across all screened cell lines and drugs. (B) Area under the dose-response curve (AUC) scores across all screened cell lines and drugs. (C) Full list of significant ANOVA interactions (both Pan-cancer and Cancer-specific ones), with hyperlinks to web-pages containing plots and all statistical scores. (D) Tabular summary of Pan-Cancer and Cancer-Specific ANOVA interaction effect sizes. (E) Set of overlapping cell lines between our study and the Cancer Cell Line Encyclopedia dataset (CCLE; Barretina et al., 2012). (F) Set of overlapping compounds between our study and the CCLE (Barretina et al., 2012). (G) Drug response indicators (IC*50*s s and Activity area for the CCLE, and IC*50*s s and Area under the response curve for our study) for the set of overlapping cell lines and compounds in Tables S4E and S4F, together with additional screening information, such as maximal tested concentrations across the two screenings, etc. (H) Set of significant and robust pharmacogenomic interactions identified in our study that are testable on the CCLE and have been validated on this independent dataset on the set of overlapping cell lines and compounds in Tables S4E and S4F, together with hyperlinks to webpages with scatterplots and statistical scores across the two studies. (I) Set of overlapping cell lines between our study and the Cancer Target Discovery And Development (2015 version) (CTRP, Seashore-Ludlow et al., 2015). (J) Set of overlapping compounds between our study and the CTRP (Seashore-Ludlow et al., 2015). (K) Drug response indicators (Area under the dose response curve for CTRP, and IC*50*s and Area under the response curve for our study) for the set of overlapping cell lines and compounds in Tables S4I and S4J, together with additional screening information, such as maximal tested concentrations across the two screenings, etc. (L) Set of significant large-effect size pharmacogenomic interactions identified in our study that are testable on the CTRP and have been validated on this independent dataset on the set of overlapping cell lines and compounds in Tables S4I and S4J, together with hyperlinks to webpages with scatterplots and statistical scores across the two studies.

Table S5. LOBICO Models and Validation on an Independent Dataset, Related to Figure 5 (A) SPEED Pathway activity scores across cell lines and pathways. (B) SPEED signature reliability scores. (C) Binarized IC*50*s across all screened cell lines and drugs. (D) Binarized SPEED pathway activity scores, across cell lines and pathways. (E) Full list of significant LOBICO models, with hyperlinks to webpages containing plots, statistical scores and ROC analysis outcomes. (F) Interesting AND and OR combinations uncovered by LOBICO. (G) LOBICO models for CTRP validation (legend in file).

Table S6. Machine Learning Performances, Related to Figure 6 Performances of the machine learning models across individual data types and their combinations

Table S7. Projecting the Identified Pharmacogenomic Markers Back onto Patient Tumors, Related to Figure 7 (A) Incidence of drug sensitivity markers identified through ANOVA in the primary tumor samples. (B) Incidence of drug resistance markers identified through ANOVA in the primary tumor samples. (C) Percentages of primary tumors samples satisfying selected formulas of drug sensitivity.

Document S2. Article plus Supplemental Information
