# Supplementary material for: A Landscape of Pharmacogenomic Interactions in Cancer
Source: Cell. 2016 Jul 28;166(3):740–54. doi: 10.1016/j.cell.2016.06.017 (PMC4967469; doi:10.1016/j.cell.2016.06.017)
Supplement: Document S1. Supplemental Experimental Procedures and Data S1 [file mmc1.pdf]

Cell, Volume *166*

Supplemental Information

A Landscape of Pharmacogenomic Interactions in Cancer

Francesco Iorio, Theo A. Knijnenburg, Daniel J. Vis, Graham R. Bignell, Michael P. Menden, Michael Schubert, Nanne Aben, Emanuel Gonçalves, Syd Barthorpe, Howard Lightfoot, Thomas Cokelaer, Patricia Greninger, Ewald van Dyk, Han Chang, Heshani de Silva, Holger Heyn, Xianming Deng, Regina K. Egan, Qingsong Liu, Tatiana Mironenko, Xeni Mitropoulos, Laura Richardson, Jinhua Wang, Tinghu Zhang, Sebastian Moran, Sergi Sayols, Maryam Soleimani, David Tamborero, Nuria Lopez-Bigas, Petra Ross-Macdonald, Manel Esteller, Nathanael S. Gray, Daniel A. Haber, Michael R. Stratton, Cyril H. Benes, Lodewyk F.A. Wessels, Julio Saez-Rodriguez, Ultan McDermott, and Mathew J. Garnett

SUPPLEMENTAL EXPERIMENTAL PROCEDURES

Table of Contents

1. Variant identification in cell lines
2. Microsatellite instability data
3. Selection of cancer driver genes
4. Variants recurrence filter
5. Identification of recurrently copy number altered chromosomal regions
6. Cell line methylation profiling
7. Identification of informative CpG island and methylation data discretisation
8. Transcriptional data pre-processing
9. Tumors/cell lines integrated analysis: Frequency profile comparisons
10. Tumors/cell lines integrated analysis: Knn classification performances and euclidean embedding
11. Tumors/cell lines integrated analysis: identification of classes of samples and selected functional events
12. Tumors/cell lines integrated analysis: enrichment analysis of event types across the identified classes
13. Tumors/cell lines integrated analysis: enrichment analysis of individual cancer functional event occurrences in a given class
14. Cell viability assay, dose response curve fitting model, comparison across replicates and compound cluster analysis
15. ANOVA model, effect-size, significance and p-value correction
16. ANOVA down-sampling studies
17. Pathway activity signatures and inference (SPEED analysis)
18. LOBICO to predicted drug response
19. LOBICO: cross-validation and feature importance scores
20. LOBICO: statistical test to identify predictive models
21. LOBICO: finding interesting pairwise AND and OR combinations
22. LOBICO: plotting a selected group of AND and OR combinations
23. Model selection, filtering and projection on primary tumor data
24. Validation of the ANOVA interactions and LOBICO models on independent datasets
25. Machine learning models

1. Variant identification in cell lines

After sequencing variants were identified by comparison to a reference genome. A matched lympoblastoid line was used as the reference genome for a small subset (n=39) of the cell lines where this was available. Differences from the reference genome were identified using the CaVEMan and Pindel algorithms identifying substitution and small insertions/deletions, respectively (https://github.com/cancerit). The resulting variants were then screened against approx. 8,000 normal samples to remove sequencing artefacts and germline variants (428 in-house normal exomes, 6500 normal exomes (NHLBI GO Exome Sequencing Project, June 20th 2012 release), 1000 genomes project (29th March 2012 release)) as well as variants in the DBSNP database (only those with associated minor allele frequency).

The remaining putatively somatic variants were classed as validated if present in other large scale cell-line sequencing datasets (the CCLE targeted sequencing (Barretina et al., 2012), NCI60 exome sequencing or previous capillary sequencing of 70 known cancer genes across 770 cell lines (Garnett et al., 2012)) and all such validated variants, together with other high quality variants (read depth ≥ 15 and a mutant allele burden ≥ 15% with no reads in the reference normal) were entered into the COSMIC cell line project database. Additional validation was carried out for putatively oncogenic ‘low confidence’ variants seen in

genes listed in the Cancer Gene Census (v67). Several transcripts are listed in COSMIC for some genes and this results in duplication of variants when exported, such duplicates were removed from the dataset.

All the BAM files have been deposited on the European Genome-phenome Archive (accession number: EGAS00001000978).

2. Microsatellite instability data

Analysis of microsatellite instability (MSI) was carried out according to the guidelines set down by "The International Workshop on Microsatellite Instability and RER Phenotypes in Cancer Detection and Familial Predisposition" workshop. Samples were screened using the markers BAT25, BAT26, D5S346, D2S123 and D17S250 and were characterised as MSI if two or more markers showed instability.

3. Selection of cancer driver genes

As described in (Rubio-Perez et al., 2015) we identified a set of high confidence cancer genes and we filtered individual variants based on the their occurrence frequency in COSMIC (v68) (http://cancer.sanger.ac.uk/cosmic/), as follows. We analysed sequence data from 6,815 matched tumor normal sample sets collated from 48 tumor-resequencing cohorts covering 28 major human cancer types. Briefly, three methods identifying complementary signals of positive selection were used: (a) OncodriveFM (Gonzalez-Perez and Lopez-Bigas, 2012), which identifies genes biased towards the accumulation of mutations with high functional impact; (b) OncodriveCLUST (Tamborero et al., 2013a), which identifies genes with an abnormal clustering of mutations across the protein sequence; and (c) MutSigCV (Lawrence et al., 2013), which identifies genes mutated at frequencies significantly above the background mutation rate. Genes stated as non-expressed in the corresponding cancer tissue were excluded from the observation set but used for the construction of the background models. The analysis was performed for each tumor cohort and in a pooled sample set to increase the statistical power to detect lowly-recurrent drivers acting consistently across several tumor types. The results of each method were combined by following the rationale explained by (Tamborero et al., 2013b). This analysis identified 461 cancer genes, which were classed as having evidence levels of A, B or C, as below and where genes with the former had the strongest evidence for selection and C the weakest. Level A = the gene exhibited more than one signal of positive selection (significant by more than one method) across one cohort of somatic mutations; Level B = the gene displayed only one single signal of positive selection in a certain cohort, and was a well-established cancer driver (i.e. included in the Cancer Gene Census); and Level C = the gene displayed only one single signal of positive selection, and was connected through a protein-protein or otherwise functional interactions (in the Reactome (Croft et al., 2014) or the PathwayCommons networks (Cerami et al., 2011)) to a gene with A or B evidence levels.

A recent analysis of nonsense mutations across 7,651 diverse tumors identified 55 putative recessive oncogenes (Wong et al., 2014), 46 of which were present in the set of 461 genes described above. The remaining 9 genes were added to the list of cancer genes (*AMOT*, *ASXL2*, *FTSJD1*, *LARP4B*, *MBD2*, *PHLPP1*, *RNF43*, *SACS* and *ZNRF3*). Therefore the final set consisted of 470 high confidence cancer genes.

We compared these results with the current version of the Cancer Gene Census, a manually curated catalogue of cancer genes, which is widely used as a benchmark (http://cancer.sanger.ac.uk/census). The current version (v72) contains 189 cancer genes driven by somatic point mutations, nonsense and frame shift mutations, of which 99 were identified in 1 or more of the 27 tumor types studied. The majority of the Cancer Gene Census genes not identified in this study are either found in tumor types not included in this analysis (e.g. basal cell carcinoma) and/or are mutated at very low frequency (e.g. FANCA in AML).

4. Variants recurrence filter

Variants from both the cell lines and tumors were screened against the ‘systematic screen data’ from COSMIC (v68) (derived from large-scale clinical datasets) to identify the recurrent variants most likely to contribute to carcinogenesis (‘driver mutations’). We defined recurrence for missense variants, which are

generally activating and therefore 'pile-up' within a limited number of codons within the gene, differently to protein truncating mutations, which are inactivating events that occur across the footprint of the gene. For missense variants the number of non-synonymous variants in each codon of all genes within the systematic screen data in COSMIC (v68) was calculated, and any codon with ≥ 3 variants was classed as recurrently mutated. Inframe indels were treated in the same manner (although separately). With regards to protein truncating variants all genes that contained > 10 truncating variants (frameshifting indels, essential splice and nonsense mutations) within the systematic screen data were classed as recurrently inactivated.

5. Identification of recurrently copy number altered chromosomal regions

Segment copy number data was downloaded from the TCGA (Cancer Genome Atlas Research Network et al., 2013) (8,182 samples) and analysed with ADMIRE (van Dyk et al., 2013). The cohorts of COAD and READ were merged due to their high similarity in tissue type and response profile. The ADMIRE analysis results comprised copy number segments statistically different from expectation. Filter criteria were defined to focus the analysis on potential driver segments. The filter list required the segments to include at least one protein coding or antisense gene, but no more than 100 of them. It required the deletions to include an exon (a proxy for gene disruption) and amplifications to span a gene (as sub-genic amplifications are unlikely to be functional). The false discovery rate (FDR) controlled p-value was required to be smaller than 0.05, and the segment was required to be at recursion level two or higher unless it was a top-level segment. To ensure clinical relevance, the identified segment needed to be affected in at least 2.5% of the subjects. The latter was evaluated on two levels, using the overall background variance, and using the local background variance. The first was calculated on the $\log_2$ values not part of any identified segment, regardless of filtering. The second was calculated on the recursion level below the identified segment.

Within each tumor type the segments obtained after filtering (Table S2D) were further compacted by pruning all overlapping segments such that only the shortest were retained. This results in a fairly concise set of segments per tumor type. The pan-cancer set of segments was derived from the entire collection of filtered cancer specific segments, but only the largest overlapping segment was retained (Table S2E).

CEL files containing copy number profiles for all the cell lines in the panel (from SNP6.0 arrays) have been deposited on the European Genome-phenome Archive (accession number: EGAS00001000978)

6. Cell line methylation profiling

DNA samples were assessed for integrity, quantity and purity by electrophoresis in a 1.3% agarose gel, Picogreen quantification, and Nanodrop measurements. All samples were randomly distributed into 96 well plates. Bisulfite conversion of 500 ng of genomic DNA was performed using EZ DNA methylation kit (Zymo Research. Irvine) following manufacturer's instructions. 200 ng of bisulfite converted DNA were used for hybridization on the HumanMethylation450 BeadChip (Illumina, Inc. San Diego). Briefly, samples were whole genome amplified followed by an enzymatic end-point fragmentation, precipitation and resuspension. The resuspended samples were hybridized onto the beadchip for 16 hours at 48ºC and washed. A single nucleotide extension with labeled dideoxy-nucleotides was performed and repeated rounds of staining were applied with a combination of fluorescently labeled antibodies differentiating between biotin and DNP. Fluorescent signal from the microarray was measured with a HiScan scanner (Illumina, Inc. San Diego) using iScan Control Software (V 3.3.29).

Raw methylation profiles have been deposited on Gene Expression Omnibus (GEO) (accession number: GSE68379).

7. Identification of informative CpG island and methylation data discretisation

After pre-processing, the beta signal of each CpG island was analysed in the context of each cancer type *C*. Pre-processed beta-signals for primary tumors and cell-lines are available at http://www.cancerrxgene.org/gdsc1000/.

Due to the high tissue specificity of the epigenomic profiles, a systematic Hartigan's dip test for unimodality was executed on each of the beta signals across the tumor samples of *C* (for each *C* with methylation data available for at least 100 tumor samples), with the aim of identifying a set of CpG islands for which this signal did not distribute unimodally. Such CpG islands were deemed unlikely to be tissue-specific, hence consistently hyper methylated or hypo methylated across all the *C* tumor samples. Additionally, they were considered *informative*, because by using their bimodal beta signal distribution is it possible to stratify the tumor samples from *C* into two classes of lowly and highly methylated samples, respectively.

Once a set of informative CpG islands was identified, for each cancer type *C*, we found that for all of them the beta signal distribution was bimodal, hence we fitted a two Gaussians mixture model distribution to each of their beta signals, using the standard Expectation-Maximization (EM) algorithm. Finally, by examining the means of the two Gaussian distributions in the resulting fitted model, we labeled them as the generator of the low and high beta values, respectively. For each informative CpG islands we fixed a discretization threshold equal to the beta value for which the posterior probability of the high-beta distribution was at least 10-fold higher than that of the low-beta distribution. For each cancer type *C* for which methylation data for less than 100 tumor samples was available (DLBC, MESO, PAAD), or no methylation data was available from a matched tumor dataset (ALL, CLL, LCML, MB, MM, NB, OV, SCLC), we used as *C*-specific set the 19 CpG islands found informative in at least 2 other cancer types. In this case, a discretization threshold for a CpG island in this pooled set was computed as the median of all of its discretization threshold values obtained from the analyses of the cancer types with available tumor data (as explained above).

The cancer specific threshold value of each informative CpG islands was finally used to discretize its corresponding beta values across both the tumor and the cell line samples from the cancer type under consideration, thus coding all the methylation datasets in a binary fashion (where 1 indicates a relatively high-level of methylation and zero means a low level).

The R-code used to perform this analysis is available on request, all the intermediate results and plots are available at http://www.cancerrxgene.org/gdsc1000/, and the final list of informative CpG islands is reported in Table S2H.

8. Transcriptional data pre-processing

Microarray data generated as specified in the Experimental Procedures of the main text, was analyzed as described on the Human Genome U219 96-Array Plate using the Gene Titan MC instrument (Affymetrix). The robust multi-array analysis (RMA) algorithm (Irizarry et al., 2003) was used to establish intensity values for each of 18562 loci (BrainArray v.10, (Dai et al., 2005)).

Raw data was finally deposited in ArrayExpress (accession number: E-MTAB-3610). The RMA processed dataset is available at http://www.cancerrxgene.org/gdsc1000/.

9. Tumors/cell lines integrated analysis: Frequency profile comparisons

For each data-omic *D*, cancer type *C*, and sample type *T* (cell lines or tumors) a frequency profile *P(D,C,T)* was assembled, containing *n* entries: one for each of the cancer functional events (CFEs) involving genomic features from the *D* data-omic (i.e. high confidence cancer driver gene mutations, copy number alterations of recurrently amplified/deleted chromosomal regions, methylation status of informative CpG islands). The value of the *i-th* entry of this profile corresponded to the percentage of *T* samples from cancer type *C* in which the *i-th* CFE was present.

Once all these profiles were assembled for all the possible *(D,C,T)* triplets, for a given *D* we computed Pearson's correlation (*R*) scores between each pair of profiles *P(D,C,tumors)* and *P(D,C,cell lines)*, for all the cancer type *C* for which *D* data was available for tumors and cell lines both. Grouping the resulting *R*-scores based on the omic *D* and arranging them into pairwise comparison matrices M_D (containing *m* rows and columns, with *m* = number of cancer types for which *D* data was available for cell lines and tumor

both), which the generic entry *i,j* contains $R(P(D,C_i,tumors), P(D,C_j,cell\ lines))$, yielded the results showed in the heatmaps of Figure 2B and Table S2L. To avoid correlation boosts due to consistently high frequency of occurrence across all the cancer types and sample types, the CFE accounting for *TP53*-mutations was excluded from these comparisons. Additionally to make the *R*-scores comparable across different cancer types, the same set of CFE (i.e. the pan-cancer set) was used to assembled each *P(D,C,T)* profile.

The tendency of the *R*-scores computed between profiles of cell lines and tumors from the same cancer type to be higher than those computed between profiles of cell lines and tumors from different cancer types (i.e. the statistical difference between the on/off diagonal values of the three M_D matrices) was quantified with a Welch's t-test (yielding the box plots in Figure 2B).

10. Tumors/cell lines integrated analysis: Knn classification performances and euclidean embedding

To evaluate the performances of a K-nearest-neighbors classifier in assigning the correct cancer lineage to a given Cancer Functional Events (CFEs) frequency profile (computed as described in the previous section) by looking at its closest neighbor (i.e. the most correlated other profile), and to visualise all the profiles into a low dimensional space, the *R*-score computation was extended also to cell-line versus cell-line, and tumor versus tumor comparisons. In other words, for a given data omic *D* all the possible comparison $R(P(D,C_1,T_1), P(D,C_2,T_2))$, with C_1 and C_2 both belonging to the set of cancer types for which *D* data was available for cell lines and tumors both and T_1 and T_2 can be both cell lines or tumors. After this, all the resulting *R*-scores were scaled in [0,1] on a data-omic basis, then averaged across the different omics. The resulting final averaged set of *R*-scores (Table S2M) was finally used to compute the performances of a K-nearest neighbor classifier (Figure S2E) and to embed all the profiles into a bi-dimensional space where their pair-wise distance is inversely proportional to their pair-wise correlation (Figure S2D). This was obtained by making use of the t-SNE tool (Van der Maaten and Hinton, 2008), by giving as input to the dimensional scaling algorithm the final averaged set of *R*-scores, and performing 5,000 optimization-iterations with a perplexity parameter equal to 50% of the number of points to be embedded (i.e. the total number of cell lines and tumors frequency profiles).

11. Tumors/cell lines integrated analysis: identification of classes of samples and selected functional events

Two multi-omics datasets were assembled, respectively for cell lines and tumors, by pooling together all the Cancer Functional Events (CFEs) occurrence profiles across all the cell line and tumor samples and the three data omics layers (i.e. mutations, copy number alterations and hypermethylations). Subsequently these two datasets were merged together (Table S3B) and modeled as a bipartite network *G* (Table S3C, in simple interaction format (sif)), where the first class of nodes corresponded to CFEs, the second class of nodes corresponded to samples (both cell lines and tumors) and a link connected the node corresponding to the *i-th* CFE to the node corresponding to *j-th* sample, if the *i-th* CFE occurred in the *j-th* sample. A fast community detection algorithm, based on a greedy strategy (Newman, 2004) was then applied to this network, yielding 4 communities (i.e. groups of densely interconnected nodes), or classes, containing both CFE and sample nodes.

12. Tumors/cell lines integrated analysis: enrichment analysis of event types across the identified classes

Given a class $C = \{S,E\}$, where *S* is a set of sample-nodes and *E* is a set of cancer functional event (CFE) nodes, the statistical enrichment of a given CFE type *T* (i.e. mutated high confidence cancer genes, amplified and delete recurrently copy number altered chromosomal regions, and hypermethylated informative CpG islands) in *C* was quantified through a p-value assignment derived from an hypergeometric test with parameter *x, k, n, N*, where *N* was the total number of nodes corresponding to CFEs of any type in the bipartite network *G* (modeling the combined cell-lines/tumors dataset); n was the total number of nodes corresponding to CFEs of type *T* in in *G*; $k = |E|$ was the total number of CFE nodes in class *C*; *x* was the total number of nodes corresponding to CFE of type *T* in class *C*.

13. Tumors/cell lines integrated analysis: enrichment analysis of individual cancer functional event occurrences in a given class

Given a class $C = \{S,E\}$, where S is a set of sample nodes and E is a set of Cancer Functional Event (CFE) nodes, and an individual CFE e belonging to E. The number of occurrences of e across all the samples in the combined datasets was equal to the degree of the corresponding node in G, defined as above, (i.e. number of sample nodes connected to the e node). Then the statistical enrichment of these occurrences in class C was computed through a p-value assignment derived from an hypergeometric test with parameter x, k, n, N, where N was the total number of occurrences of all the CFEs across all the samples of the combined cell-line/tumor datasets (equal to the total number of links in the network G); n is the total number of occurrences of e in the combined cell-line/tumor datasets (equal to the degree of the e node in G); k was the total number of links between nodes in S and nodes in E (i.e. the total number of links in class C); x was the total number of links connecting the e node to nodes in S (i.e. the total number of links connecting e to the sample nodes of C).

14. Cell viability assay, dose response curve fitting model, comparison across replicates and compound cluster analysis

The majority of the screened compounds were sourced from SelleckChem. We have adopted industry standards for the storage of compounds under inert conditions (low O2, dark, low humidity) to maximize their stability. Compound stability and precipitation have been monitored through visual inspection of compound solutions and using an Echo acoustic dispenser plate audit function. Furthermore, the same 10 cell lines have been screened every 2 - 3 months with compounds to confirm that compound activity was retained.

Cells were seeded in 384-well microplates at ~15% confluency in culture medium with 10% FBS and Penicillin/Streptomycin. The optimal cell number for each cell line was determined to ensure that each was in growth phase at the end of the assay (~85% confluency). For adherent cell lines, after overnight incubation cells were treated with either 9 concentrations of each compound (2-fold dilutions series), or 5 concentrations of each compound (4-fold dilution series), using liquid handling robotics (Beckman Coulter), and then returned to the incubator for assay at a 72-h time point. Cells were fixed in 4% formaldehyde for 30 minutes and then stained with 1μM of the fluorescent nucleic acid stain Syto60 (Invitrogen) for 1 hour. For suspension cell lines, cells were treated with compound immediately following plating, returned to the incubator for a 72-h time point, then stained with 55 μg/ml Resazurin (Sigma) prepared in Glutathione-free media for 4 hours. Quantitation of fluorescent signal intensity is performed using a fluorescent plate reader at excitation and emission wavelengths of 630/695 nM for Syto60, and 535/595 nM for Resazurin.

All screening plates were subjected to stringent quality control measures and to assess the quality of our screening a Z-factor score comparing negative and positive control wells was calculated across all screening plates.

In estimating the characteristic IC_{50} dose-response value, all data is fitted in a single model (Vis et al., 2016). The assumed dose-response model is a two-parameter sigmoid that models the relative viability. The latter is obtained by scaling the observed intensities to the mean intensities of the control wells. Since the assumed response is strictly between 0 and 100% relative viability, the values are capped at 0 and 100.
Particularly, in this non-linear mixed effect model, it is assumed that the position and shape parameters vary.

$$f(x_{pos_{ij}}, x_{shape_i}, x) = \frac{1}{1 + e^{-\frac{x - x_{pos_{ij}}}{x_{shape_i}}}}$$

The parameters x_{pos} and x_{shape} are allowed to vary across the cell lines and for each cell line-drug pair a drug specific deviance is accommodated. On the cell line level, the two parameters are assumed to be correlated. The variation due to the drug is nested in the cell line level. The model is fitted using The R Project for Statistical Computing package nlme (Pinheiro et al., 2007). The x-positions represent the concentration dilution series, in which nine is the highest and one is the lowest screening concentration, such that a unit decrease translates to a two-fold dilution. Doubling the interval on x accommodates

alternative designs with five concentration points and four-fold dilutions. The interpretation of the dose-response curve as a function of the dilution series removes the scaling differences in the maximum test concentrations between the drugs, which ranges from the low nanoMolar (Bryostatin 1) to the milliMolar (DMOG) range. Note that the IC_{50}s are recorded as the natural logarithm of the half-maximal inhibitory μM concentrations

To evaluate reproducibility of results we screened 7 compounds in biological replicates observing a median Pearson correlation between patterns of IC_{50} values across cell lines equal to 0.65 when considering all the 7 compounds, and equal to 0.78 when considering the compounds for which the majority of compared IC_{50} values fell within the range of tested concentrations in at least one replicate (Figure S1). Furthermore, the sets of sensitive ($IC_{50} \leq$ maximal tested concentration) and resistant ($IC_{50} >$ maximal tested concentration) cell lines were highly overlapping across replicates, yielding statistically significant Fisher exact test p-values for all the 7 compounds.

Finally we performed a cluster analysis of the screened compounds based on similarity of their AUC profile across cell lines. Cell lines and compounds with less than 50% of AUC values available were not considered in this analysis and remaining missing AUCs were imputed with a k-nearest-neighbour (knn) approach. The first filtering reduced the original dataset (containing AUC values for 81% of all the possible compound/cell-line combinations) from 265 drugs and 990 cell lines to 223 drugs and 925 cell lines (with AUC values for 92% of the compound/cell-line combinations). On this dataset, the remaining 8% of missing AUCs were imputed through knn. The cluster analysis was performed through consensual non-negative matrix factorization (Brunet et al., 2004), with clustering cardinalities k ranging in [5,30], a maximal number of 500 iterations in each clustering and 20 clustering trials for each k. The optimal number of clusters was determined as a trade-off between the best cophenetic coefficient across the different values of k and the maximal k. Results are reported in Table S1G, together with a silhouette width score for each samples, quantifying how much it is well-placed in its cluster.

15. ANOVA model, effect-size, significance and p-value correction

A drug–response vector consisting of n IC_{50} values from treatment of n cell lines was constructed for each drug. The model was linear (no interaction terms) with dependant variables represented by the described vector and factors including tissue type, and screening medium (for the pan-cancer analysis only), micro-satellite instability status (for the cancer types with positive samples for this feature) and the status of a CFE (one model for each CFE). These factors were selected based on a preliminary analysis assessing their impact on differential drug response in the pan-cancer context as well as for all the cancer types included in the study. This was performed through a type-II error ANOVA modelling drug response as a linear combination of the tissue of origin of the cell lines (only in the pan-cancer context), screening medium (i.e. DMEM or RPMI/F12) and growth properties (i.e. adherent, semi-adherent or suspension) (Table S1E). We observed that for growth properties these variables are essentially homogenous for cell lines within a specific cancer type, and so the contribution of these factors to the cancer-specific analysis is negligible. In contrast, media type has an impact on the response to several compounds when performing a pan-cancer analysis.

For the pan-cancer analysis, the union of all the cancer-specific CFEs (across omics layers and cancer types) was used. To reduce the number of tests, the set of informative CpG islands were excluded from this analysis. Additionally, only CFEs occurring in at least 3 cell lines were considered and CFEs with identical pattern of positive occurrence were merged together, thus resulting into a final set of 677 (individual or combined) features across 987 cell lines (screened against at least 1 drug). In order to include as many cell lines as possible in the pan-cancer analysis (even those not matching a TGCA type), values of the tissue factor were determined by looking at the GDSC.description_1 label in the cell lines annotation file (Table S1E). Whereas for the cancer-specific analysis, only cell lines with a matching TCGA label were used. The tissue factors corresponding to 'digestive_system' and 'urogenital_system' were further sub-classed by using the more specific GDSC.description_2 label.

For all the tested gene-drug associations, effect size estimations versus pooled standard deviation (quantified through the Cohen's d), effect sizes versus. individual standard deviations (quantified through

two different Glass deltas, for the CFE positive and the CFE negative population respectively), CFE p-values and all the other statistical scores where obtained from the fitted models. A CFE-drug pair was tested only if at least three cell lines were contained in the two sets resulting from the dichotomy induced by the CFE-status (i.e. at least 3 positive cell lines and at least 3 negative cell lines), for the pan-cancer and all the cancer-specific analyses as well.

The resulting p-values were corrected (all together those obtained in the pan-cancer analysis and on a cancer type basis those obtained in a given cancer-specific analysis), with the Tibshirani-Storey method (Storey and Tibshirani, 2003). A p-value threshold of 10^{-3} and a false discovery rate threshold equal to 25% were finally used to call significant associations across all the performed analyses. A pan-cancer and 18 cancer-specific (where at least 15 cell-line samples and sequencing and copy-number variation data was available for primary tumors) analyses were performed.

A Python package implementing the ANOVA analysis described in this section is available at http://gdsctools.readthedocs.io, together with detailed instructions on how to reproduce the results presented in our manuscript.

16. ANOVA down-sampling studies

This analysis aimed to assess whether the tendency of the cancer-specific interactions to associate with larger effect sizes (compared to the pan-cancer interactions) originates solely from the population-size reduction. Four cancer types (BRCA, COAD/READ, SKCM and LUAD) were selected because they are among those with the largest numbers of cancer specific associations and more than 20 available samples.

For a cancer type C with n available samples, 10 down-sampled pan-cancer ANOVAs were executed. To perform each of these analyses, a simulated cell line dataset was composed by randomly selecting n samples from the pan-cancer input matrix of features and their columns corresponding to their corresponding positive CFEs. Then effect-sizes of the resulting ANOVA tests (for fixed levels of significance) where compared across pan-cancer, down-sampled and C-specific analyses. As expected, all the tested cancer types showed a strong consistent increase of effect size due to the down-sampling. Nevertheless, within the cancer specific analyses, this increment was significantly more evident and less variable.

A second down-sample ANOVA study was conducted to determine how many of the statistical significant and large-effect pharmacogenomic interactions (p-value < 0.001, FDR < 25%, Glass Δs > 1) reported in our study (S) would be still detectable if using reduced cell line sub-sets. To this aim, for n = 500, 300, 150, and 60, and a number of iterations $k = 1,\ldots,50$, n cell lines were randomly selected from the whole panel (reported in Table S1E), in a way that the spectrum of values of the tissue factor used in the pan-cancer ANOVA (assembled as described in the supplementary experimental procedures section 15), was preserved. On this set of cell lines a pan-cancer and 18 cancer-specific ANOVAs were performed. The number of significant and large-effect interactions S_k, were then determined in each of these k iterations and the overlap between S_k and S assessed. The average cardinalities of these overlaps across all the iterations were then collected for all n values, and reported in Figure S4D, S4E and S4F. These results show that only a limited number of the interactions identified based on an analysis of the whole panel of 991 screened cell lines would have been still detectable on n cell lines, with an average loss of 95% of high confidence cancer specific interactions and a loss of 70% of high confidence pan-cancer interactions already at the first n (= 500, Figures S4D, S4E and S4F).

17. Pathway activity signatures and inference (SPEED analysis)

To infer the activity of different signaling pathways, we used activation signature genes provided by the SPEED platform (Parikh et al., 2010). These are derived from multiple activation-response experiments using a total of 215 perturbations performed in 77 different experiments and covering 11 different signaling pathways [URL: http://speed.sys-bio.net/cgi-bin/database_statistics.py]. We chose this platform in favor of more recently derived breast cancer activation signatures (Gatza et al., 2010; 2014) to cover a large variation in experimental conditions and obtain signatures that are the consensus between different tissues. Those signatures represent genes that are differentially expressed when a pathway is perturbed. Comparing the expression level of those perturbation-response genes in the basal expression profile of different cell

lines gives us insight about the constitutive activity caused by mutations, copy number alterations, or other signaling aberrations. This activity may represent well-known mutational activations, but more importantly can also represent signaling activation for which there is no clear mutational marker available.

In order to derive consensus signature sets of genes for 11 different pathways the authors of (Parikh et al., 2010) defined the following quantities: z-scores – the number of standard deviations a certain gene is expressed in the perturbed condition compared to the basal condition; overall expression – the percentile of top expressed genes that a gene needs to belong to in order to be considered; overlap – in which percentage of experiments this needs to be the case; and uniqueness – if a given gene can be associated to more than one pathway.

In the original SPEED publication the authors suggested to use a constant cutoff of $z < 1\%$, expression > 50%, overlap > 20% and allowing non-unique genes. However, they only evaluated gene lists by their overlap with Gene Ontology categories, and not based on how well their enrichment scores are able to differentiate between microarrays where a given pathway perturbation is present and those where it is absent. This resulted in pathways that were highly correlated, as shown in Figure S5A. To counteract this, we performed a scan of the four adjustable platform parameters to optimize the order obtained by GSEA scores between control and stimulated experiments. The set of control arrays comprised all the un-stimulated arrays in the database, and the stimulated set all arrays in the database where a certain pathway was perturbed. This way, we allow cross-activation of pathways while minimizing the fit to random differences in gene expression by different initial conditions. We trained the parameters using 5x cross validation and selected the model with the best fit on the part of the data set not used for training. As a performance measure we used the area under the precision-recall curve that was obtained by the order of raw GSEA scores. Table S5B shows the improved separation between basal and pathway-perturbed arrays.
We calculated the raw basal GSEA scores for all cell lines using the gene lists obtained. As we do not have a background set, we transformed the resulting bimodal distribution for each pathway across all cell lines into a normal distribution using a kernel density estimator (kCDF function from the R package sROC) followed by the transformation $\log(x/(1 - x))$.

18. LOBICO to predicted drug response

As binary input features we used the set of cancer functional events (CFEs), excluding iCpGs and including gene expression derived binary pathway activity scores computed with the SPEED framework (Parikh et al., 2010) as detailed in SEP17.

Each of the 11 SPEED signatures resulted in two binary features: one representing upregulation by thresholding the continuous SPEED activity scores at a value of 3, and one representing downregulation (thresholding at a -3). These threshold values corresponded to three standard deviations above/below the mean of the normalized scores. The output feature was the response of the cancer cell lines to an anti-cancer drug as measured by the IC_{50}, discretized as detailed in below.

LOBICO was employed in a pan-cancer and 18 cancer-specific analyses and run for each drug individually, when the number of sensitive cell lines was 5 or higher. A cross-validation strategy was employed and feature importance scores derived as detailed in SEP19. Statistical tests to identify predictive models, selection of interesting pairwise AND and OR combinations and representative cases highlighted in Figure 5C, are detailed in the SEP20 to 22.
LOBICO finds the optimal logic function of the binary features that minimizes the error, defined as the sum of the weighted misclassified cell lines. The weight is proportional to the binarization threshold distance. Consequently, misclassification of cell lines close to the binarization threshold does not affect the optimization criterion, whereas there is a large penalty for misclassifying cell lines that are extremely sensitive or resistant to the drug. The total weight of each class is normalized in order to balance class importance. LOBICO was constrained to find solutions with a specificity of 0.8 or higher and applied with the same eight different complexities as in (Knijnenburg et al., 2016) ranging from simple single-predictor models to multi-input AND and OR models.

Note that the IC_{50}s were recorded as the natural logarithm of the half-maximal inhibitory μM concentrations. Although LOBICO uses the continuous IC_{50} values, it is necessary to define a binarization threshold. This threshold is used to divide the cell lines into two classes: the sensitive cell lines and the resistant cell lines. We employed the procedure described in (Knijnenburg et al., 2016) to find the binarization threshold for each of the drugs. The binarization threshold for each of drugs was determined using all cell lines in the pan-cancer dataset.

There is one minor change in the 'upsampling' step of this procedure with respect to (Knijnenburg et al., 2016): In (Knijnenburg et al., 2016), the standard deviation of an IC_{50} was derived from the confidence interval of the IC_{50}. Here, the standard deviation of an IC_{50} was set 0.2, which was the typical across all IC_{50}s. This adjustment was made because we employed a different curve fitting algorithm compared to our previous work (Garnett et al., 2012). The new curve fitting algorithm does not provide a confidence interval per IC_{50}. Additionally, parameter t was set to 0.03 instead of 0.05 in (Knijnenburg et al., 2016).

19. LOBICO: cross-validation and feature importance scores

For each LOBICO analysis, a stratified 5-fold cross-validation (CV) strategy was employed. The 5-fold CV was repeated 10 times for the cancers-specific datasets and 5 times for the pan-cancer dataset with different random seeds for assigning samples to test and training folds. We derived feature importance (FI) scores for each of the binary features for each inferred logic model of a certain model complexity. Additionally, we computed aggregated FI scores, which are based on all models complexities that have a CV error equal or smaller than the CV error for the single-predictor model.

20. LOBICO: statistical test to identify predictive models

We implemented a straightforward statistical test to decide whether an inferred logic model performed significantly better than random. First, we selected the optimal model complexity of the employed logic model, i.e. the model complexity with the lowest average CV error. Then, we took the inferred class labels (sensitive (1) and resistant (0)) of the inferred logic model (with the optimal complexity) when applied to the test folds in the CV. We did this for each of the repeats, and counted the average number of predicted sensitive (1) cell lines across the repeats, say x. We created 1,000,000 binary vectors with the original length (number of cell lines) with ones in x random positions and recorded the error (i.e. the sum of the weighted misclassified cell lines) associated with each permutation. The permutated errors, the mean of which is 0.5, were compared to the original CV error. The permutation test P-value was computed using EPEPT (Enhanced P-value Estimation for Permutation Tests) (Knijnenburg et al., 2011; 2009). This was done for all 1112 logic models. We derived Q-values for each of the 1112 P-values. A logic model was called 'predictive' when both its p-value and q-value (FDR) were smaller than 0.05. The performance of the inferred logic models was measured using the normalized CV error. This error is between 0 and 1, and is 0.5 in the case of randomly predicting the sensitivity of cell lines.

21. LOBICO: finding interesting pairwise AND and OR combinations

We identified relevant AND and OR combinations of CFEs that explain drug sensitivity. We considered the AND and OR pairs that met the following constraints across all repeats (5 repeats for the pan-cancer dataset and 10 repeats for the cancer-specific datasets):

- The pairwise combination should be present in the logic formula of the optimal or sub-optimal solution of a predictive model (p-value < 0.05, q-value <0.05), either in the logic formulas associated with the optimal model complexity (according to CV) or in logic formulas with lower model complexity.
- The pairwise combination should be present in the predictive model of a drug in at least 4 of the 6 folds (the 5 CV training folds plus the model on the complete dataset).
- The pairwise combination should be found across at least 2 drugs in the same drug class.
- The feature importance (FI) score of each member of the pair should be higher than 0.03.
- The sum of the feature importance scores of both members of the pair should be higher than 0.10.

22. LOBICO: plotting a selected group of AND and OR combinations

The arrows that are highlighted in color and annotated with text in Figure 5C meet the following constraints (applied in this order):

- The drug associated with the arrow is clinically approved as notated in Table SF1.
- The difference in the F-measure (harmonic mean of precision and recall) when going from the single predictor model to the combination is at least 0.04.
- The improvement in precision or recall when going from the single predictor model to the combination is at least 0.10.
- At most one feature in a combination can be a binarized pathway activity (SPEED signature).
- Per quadrant, a combination cannot occur more than twice. (The same combination can occur for different drugs and different cancer types.) In case of more than two instances of the same combination, we selected the two with the highest differential F-measure.
- Per quadrant, a drug cannot occur more than once per cancer type. In case of more than one instance of the same drug for a particular cancer type, we selected the one with the highest differential F-measure.
- Per quadrant, a drug cannot occur more than three times. In case of more than three instances of the same drug, we selected the three with the highest differential F-measure.
- Per quadrant, no more than two colored arrows per cancer type are allowed. In case of more than two instances of the same cancer type, we selected the two with the highest differential F-measure.

The grey arrows in the background represent all combinations mentioned in the main text and are available in Table S5F. However, for clarity at most 10 grey arrows were plotted per cancer per quadrant based on the differential F-measure.

23. Model selection, filtering and projection on primary tumor data

The models shown in Figure 7B have been selected as follows: from the whole list of predictive models in Table S5E, we selected those (i) obtained in cancer-specific analyses, (ii) guaranteeing a cross-validation error lower than 0.25, (iii) not including SPEED pathway activity scores, (iv) not including negations only as terms, (v) not including (for the cancer type under consideration) not-cancer-specific cancer genes, (vi) referring to cancer types for which primary tumor samples with both mutations and copy number alteration data were available.

From the selected models we removed terms containing features whose relative importance is lower than 0.05, and dropped out the resulting filtered models with only one input feature in input.
The final list of selected models is contained in Table S7C. From this list, those models for which the difference between the percentage of primary-tumor and cell-line samples satisfying them was greater than 25 were not visualized in Figure 7B.

24. Validation of the ANOVA interactions and LOBICO models on independent datasets

Drug response data in the Cancer Cell Line Encyclopaedia (CCLE) (Barretina et al., 2012) dataset (latest version) was downloaded from the CCLE web-portal:
http://www.broadinstitute.org/ccle/data/browseData?conversationPropagation=begin
(file: CCLE_NP24.2009_Drug_data_2015.02.24.csv: Pharmacologic profiles for 24 anticancer drugs across 504 CCLE lines, 24-Feb-2015).

Drug response data in the Cancer Therapeutic Response Portal version 2 (CTRP) dataset (Seashore-Ludlow et al., 2015) was downloaded from the supplementary information files of the corresponding main publication, available online on the Cancer Discovery journal web-site at:
http://cancerdiscovery.aacrjournals.org/content/early/2015/10/14/2159-8290.CD-15-0235/suppl/DC1
(Supplemental Tables S1 – S7, file: 145780_2_supp_3058746_nrhtdz.xlsx).

From these files, identifiers of screened cell-lines and compounds in the two studies were extracted and mapped to the cell-lines and compound identifiers of our study (refered to as GDSC). This resulted in 389 overlapping cell-lines, the $CL_{GDSC\text{-}CCLE}$ set (Table S4E), and 15 overlapping compounds for the CCLE, the $D_{GDSC\text{-}CCLE}$ set (Table S4F), and 466 overlapping cell-lines, the $CL_{GDSC\text{-}CTRP}$ set (Table S4I), and 76 overlapping compounds for the CTRP, the $D_{GDSC\text{-}CTRP}$ set (Table S4J). All the available drug response indicators included in the CCLE and CTRP where extracted for all the cell-lines and compounds in $CL_{GDSC\text{-}CCLE}$ and $D_{GDSC\text{-}CCLE}$ from the CCLE file mentioned above, and for all the cell-lines and compound in $CL_{GDSC\text{-}CTRP}$ and $D_{GDSC\text{-}CTRP}$ from the CTRP file mentioned above. For CCLE, the drug response indicators are the half-maximal inhibitory concentrations (IC_{50}s) and the Compound Activity Areas, defined as 1 – the area under the drug/cell-line dose response curve (AUC). For the CTRP the drug response indicators are the AUCs; IC_{50} values were not available for the CTRP study. These drug response indicators are contained in Table S4G and Table S4K, side-by-side with corresponding IC_{50}s and AUCs from the GDSC, together with information on the range of tested concentrations across the considered screenings.

To validate the pan-cancer and cancer-specific interactions identified through ANOVA we took the following strategy:

For each of the studies CCLE and CTRP, here denoted by *S*:

i) Pan-cancer and cancer-specific high-confidence pharmacogenomic interactions $I_{GDSC\text{-}S}$ were extracted from those originally identified in the GDSC (Table S4C). This subset $I_{GDSC\text{-}S}$ included only interactions involving compounds in the $D_{GDSC\text{-}S}$ set, with an ANOVA p-value < 10^{-3}, both Glass Δs (measures of the effect size of the interaction) > 1, and FDR < 25%. This resulted in a subset of 32 interactions for $I_{GDSC\text{-}CCLE}$ and 106 interactions for $I_{GDSC\text{-}CTRP}$.

ii) Each interaction *i = (CFE, Compound)* in $I_{GDSC\text{-}S}$ was retested two times on the subset of cell lines in $CL_{GDSC\text{-}S}$ using IC_{50} values from the GDSC, and the drug response indicator from the study *S,* i.e. IC_{50} values if *S = CCLE,* and AUC values if *S = CTRP*. These newly performed ANOVA tests were executed using the same settings of the GDSC ANOVA described in SEP 15. Specifically, we used tissue of origin, screening medium, micro-satellite instability (MSI) in addition to the cancer functional event (CFE) under consideration as co-factors for pan-cancer interactions, and only MSI in addition to the CFE under consideration for cancer-specific interactions. In the case that in the GDSC or in *S*, there were not sufficient drug response observations were available for the cell lines in $CL_{GDSC\text{-}S}$ (threshold values for the ANOVA factors described in SEP 15) then *i* was deemed not-testable on the study under consideration, i.e. GDSC or *S*. For *S* = CCLE, 22 out of 32 interactions in $I_{GDSC\text{-}S}$ were re-testable on both GDSC and CCLE. For *S* = CTRP, 70 out of 106 interactions in $I_{GDSC\text{-}S}$ were re-testable on both GDSC and CTRP. In each test, the status of the CFE under consideration was determined using data from the GDSC.

iii) Each of the interactions in $I_{GDSC\text{-}S}$ that was re-testable on both the GDSC and *S* when restricting the analysis on $CL_{GDSC\text{-}S}$, was considered validated on *S* if both the re-performed ANOVA tests yielded a p-value smaller than a given threshold *P* and the test had the same ‘interaction sign’, i.e. increased drug sensitivity or drug resistance. Different threshold values for *P* values were used: 1 (i.e. concordance of the interaction sign only), 0.25 (lax threshold), and 0.05 (stringent threshold).

iv) To assess the overall reproducibility of significant ANOVA associations in GDSC and *S*, we built and analyzed contingency tables that compare counts of significant interactions in GDSC and *S*. Specifically, for each threshold value of P, we assembled a 3x3 table as follows. The rows of a 3x3 contingency table accounted for the total number of testable interactions that were significantly sensitive (1st row), non-significant (2nd row), and significantly resistant (3rd row), on $CL_{GDSC\text{-}S}$ when using IC_{50} values from the GDSC, whereas the columns accounted for the total number of testable interactions that were significantly sensitive (1st column), non-significant (2nd column), and significantly resistant (3rd column), on $CL_{GDSC\text{-}S}$ when using the drug response indicator values from *S*. Additionally, we derived 2x2 contingency tables from the 3x3 matrices by discarding the central row and column, i.e. accounting only for significant interactions in both the

studies. Fisher exact tests were performed on these contingency matrices for all the considered threshold *P* values.

v) Finally, a Pearson correlation test was performed between the two patterns of ANOVA $-log_{10}$ p-values resulting from the two re-performed tests described in *ii,* across all the testable interactions in I_{GDSC-S}.

Results from this analysis have been assembled in Table S4H and Figure S4J for *S* = CCLE, and Table S4L and Figure S4K for *S* = CTPR.

To validate the LOBICO models we followed a similar strategy. Here, we focused solely on the CTRP dataset, since the CCLE has only 15 drugs in common with the GDSC. Specifically, we ran LOBICO for the 466 common cell lines between the GDSC and the CTRP (Table S4I) for each of the 76 common compounds (Table S4J) using the same settings as for the original analysis (described in paragraphs 18 to 22). Of note, the GDSC contains rescreens or duplicate screens for 10 of the 76 compounds resulting in a total of 86 LOBICO analyses.

For each of the 86 analyses, we selected the best model according to cross-validation (CV) and applied that model to the cell lines dividing them into a group that is predicted to be sensitive and a group that is predicted to be resistant. Then, we performed a t-test comparing these two groups both for the GDSC IC_{50}s as well as for the CTRP AUCs. The t-test was only performed when both groups had at least 5 cell lines.

25. Machine learning models

Predictive models of drug activity (i.e. IC_{50}) for each compound, using 4 different molecular data types and their combinations, were built across 18 cancer types and across a pan-cancer setting with Elastic Net (EN) and Random Forests (RF), for a total of 137,726 models. All models were 1,000 times bootstrapped, with 80% of the data used for training, 10% for cross-training (determining model parameters) and the remaining 10% exclusively for testing. Based on the cross-training the EN mixing parameter (between 0 and 1, in intervals of 0.1) and number of input features were fitted for the EN regression, using the R-package 'glmnet' version 2.0-2. The RF implementation was based on R-package 'randomForest' version 4.6-10 and during cross training the number of trees was determined (between 10 and 1000, in intervals of 10). Using the test set, we estimated predictive power by calculating Pearson correlation between the predicted and observed drug activity. Models are assumed predictive if their observed Pearson correlation has a likelihood of over 90% to be derived from the informative versus the non-informative distribution across all models, which for pan-cancer and cancer-specific setting are ~0.2 (Figure S6G) and ~0.26 (Figure S6H), respectively.

SUPPLEMENTAL REFERENCES

Barretina, J., Caponigro, G., Stransky, N., Venkatesan, K., Margolin, A.A., Kim, S., Wilson, C.J., Lehár, J., Kryukov, G.V., Sonkin, D., et al. (2012). The Cancer Cell Line Encyclopedia enables predictive modelling of anticancer drug sensitivity. Nature 483, 603–607.

Brunet, J.-P., Tamayo, P., Golub, T.R., and Mesirov, J.P. (2004). Metagenes and molecular pattern discovery using matrix factorization. Proceedings of the National Academy of Sciences of the United States of America 101, 4164–4169.

Cancer Genome Atlas Research Network, Weinstein, J.N., Collisson, E.A., Mills, G.B., Shaw, K.R.M., Ozenberger, B.A., Ellrott, K., Shmulevich, I., Sander, C., and Stuart, J.M. (2013). The Cancer Genome Atlas Pan-Cancer analysis project. Nature Genetics 45, 1113–1120.

Cerami, E.G., Gross, B.E., Demir, E., Rodchenkov, I., Babur, Ö., Anwar, N., Schultz, N., Bader, G.D., and Sander, C. (2011). Pathway Commons, a web resource for biological pathway data. Nucleic Acids Res 39, D685–D690.

Ciriello, G., Miller, M.L., Aksoy, B.A., Senbabaoglu, Y., Schultz, N., and Sander, C. (2013). Emerging landscape of oncogenic signatures across human cancers. Nature Genetics 45, 1127–1133.

Croft, D., Mundo, A.F., Haw, R., Milacic, M., Weiser, J., Wu, G., Caudy, M., Garapati, P., Gillespie, M., Kamdar, M.R., et al. (2014). The Reactome pathway knowledgebase. Nucleic Acids Res 42, D472–D477.

Dai, M., Wang, P., Boyd, A.D., Kostov, G., Athey, B., Jones, E.G., Bunney, W.E., Myers, R.M., Speed, T.P., Akil, H., et al. (2005). Evolving gene/transcript definitions significantly alter the interpretation of GeneChip data. Nucleic Acids Res 33, e175.

Garnett, M.J., Edelman, E.J., Heidorn, S.J., Greenman, C.D., Dastur, A., Lau, K.W., Greninger, P., Thompson, I.R., Luo, X., Soares, J., et al. (2012). Systematic identification of genomic markers of drug sensitivity in cancer cells. Nature 483, 570–575.

Gatza, M.L., Lucas, J.E., Barry, W.T., Kim, J.W., Wang, Q., Crawford, M.D., Datto, M.B., Kelley, M., Mathey-Prevot, B., Potti, A., et al. (2010). A pathway-based classification of human breast cancer. Proceedings of the National Academy of Sciences 107, 6994–6999.

Gatza, M.L., Silva, G.O., Parker, J.S., Fan, C., and Perou, C.M. (2014). An integrated genomics approach identifies drivers of proliferation in luminal-subtype human breast cancer. Nature Genetics 46, 1051–1059.

Gonzalez-Perez, A., and Lopez-Bigas, N. (2012). Functional impact bias reveals cancer drivers. Nucleic Acids Res 40, e169.

Irizarry, R.A., Hobbs, B., Collin, F., Beazer-Barclay, Y.D., Antonellis, K.J., Scherf, U., and Speed, T.P. (2003). Exploration, normalization, and summaries of high density oligonucleotide array probe level data. Biostatistics 4, 249–264.

Knijnenburg, T., Klau, G., Iorio, F., Garnett, M., McDermott, U., Shmulevich I., Wessels L. (2016). Logic models to predict continuous outputs based on binary inputs with an application to personalized cancer therapy. bioRxiv doi: http://dx.doi.org/10.1101/036970.

Knijnenburg, T.A., Lin, J., Rovira, H., Boyle, J., and Shmulevich, I. (2011). EPEPT: a web service for enhanced P-value estimation in permutation tests. BMC Bioinformatics 12, 411.

Knijnenburg, T.A., Wessels, L.F.A., Reinders, M.J.T., and Shmulevich, I. (2009). Fewer permutations, more accurate P-values. Bioinformatics 25, i161–i168.

Lawrence, M.S., Stojanov, P., Polak, P., Kryukov, G.V., Cibulskis, K., Sivachenko, A., Carter, S.L., Stewart, C., Mermel, C.H., Roberts, S.A., et al. (2013). Mutational heterogeneity in cancer and the search for new cancer-associated genes. Nature 499, 214–218.

Newman, M.E.J. (2004). Fast algorithm for detecting community structure in networks. Phys Rev E Stat Nonlin Soft Matter Phys 69, 066133.

Parikh, J.R., Klinger, B., Xia, Y., Marto, J.A., and Bluthgen, N. (2010). Discovering causal signaling pathways through gene-expression patterns. Nucleic Acids Res 38, W109–W117.

Pinheiro, J., Bates, D., DebRoy, S., and Sarkar, D. (2007). Linear and nonlinear mixed effects models. R Package Version.

Rubio-Perez, C., Tamborero, D., Schroeder, M.P., Antolín, A.A., Deu-Pons, J., Perez-Llamas, C., Mestres, J., Gonzalez-Perez, A., and Lopez-Bigas, N. (2015). In silico prescription of anticancer drugs to cohorts of 28 tumor types reveals targeting opportunities. Cancer Cell 27, 382–396.

Seashore-Ludlow, B., Rees, M.G., Cheah, J.H., Cokol, M., Price, E.V., Coletti, M.E., Jones, V., Bodycombe, N.E., Soule, C.K., Gould, J., et al. (2015). Harnessing Connectivity in a Large-Scale Small-Molecule Sensitivity Dataset. Cancer Discov 5, 1210–1223.

Storey, J.D., and Tibshirani, R. (2003). Statistical significance for genomewide studies. Proceedings of the National Academy of Sciences of the United States of America 100, 9440–9445.

Tamborero, D., Gonzalez-Perez, A., and Lopez-Bigas, N. (2013a). OncodriveCLUST: exploiting the positional clustering of somatic mutations to identify cancer genes. Bioinformatics 29, 2238–2244.

Tamborero, D., Gonzalez-Perez, A., Perez-Llamas, C., Deu-Pons, J., Kandoth, C., Reimand, J., Lawrence, M.S., Getz, G., Bader, G.D., Ding, L., et al. (2013b). Comprehensive identification of mutational cancer driver genes across 12 tumor types. Sci Rep 3, 2650.

Turcan, S., Rohle, D., Goenka, A., Walsh, L.A., Fang, F., Yilmaz, E., Campos, C., Fabius, A.W.M., Lu, C., Ward, P.S., et al. (2012). IDH1 mutation is sufficient to establish the glioma hypermethylator phenotype. Nature 483, 479–483.

Van der Maaten, L., and Hinton, G. (2008). Visualizing data using t-SNE. Journal of Machine Learning Journal of Machine Learning Research 9, 2579-2605

van Dyk, E., Reinders, M.J.T., and Wessels, L.F.A. (2013). A scale-space method for detecting recurrent DNA copy number changes with analytical false discovery rate control. Nucleic Acids Res 41, e100.

Vis, D.J., Bombardelli, L., Lightfoot, H., Iorio, F., Garnett, M.J., Wessels, L.F.A. Multilevel models improve precision and speed of IC50 estimates. Pharmacogenomics, 17 (7) [in press] doi: 10.2217/pgs.16.15.

Wong, C.C., Martincorena, I., Rust, A.G., Rashid, M., Alifrangis, C., Alexandrov, L.B., Tiffen, J.C., Kober, C., Chronic Myeloid Disorders Working Group of the International Cancer Genome Consortium, Green, A.R., et al. (2014). Inactivating CUX1 mutations promote tumorigenesis. Nature Genetics 46, 33–38.

Supplemental Data S1

A. Cancer functional events covered by the cell lines

The median percentage of cancer-type specific (C-S) driver genes (CGs) found mutated in at least one cell line sample across individual cancer types was equal to 80% (min = 0% for CLL and max = 100% for LUAD, BRCA, GBM, OV, NB, ALL, LIHC) when considering CGs mutated in at least 5% of the tumor samples from the cancer type under consideration (Figure 2A). The median value was 22% (min = 3% for CLL, max = 83% for ALL) (Figure S2B) when considering all the CGs.

When considering C-S RACSs amplified in at least 5% of the primary tumor samples from the corresponding cancer type, the median coverage across cancer types was equal to 88% (Figure 2A) (min = 0% for ACC, THCA, LAML, KIRC and MESO, max = 100% for CESC, DLBC, ESCA, BRCA, LUAD, and PAAD). The median value was equal to 80% (min = 0% for ACC, LAML, KIRC and MESO, max = 100% for DLBC, BRCA, ESCA, and PAAD), when considering all the C-S RACSs (Figure S2B).

Similarly, the median percentage of coverage for C-S recurrently deleted RACSs (i.e. deleted in at least 5% of the tumor samples from the corresponding cancer type), was equal to 78% (Figure 2A) (min = 0% for ACC, max = 100% for PAAD, LUAD and MESO). The median value dropped to 70% when considering all the deleted RACSs (Figure S2B) (min = 0% for ACC, and max = 100% for PAAD, LUAD, and MESO, in both the cases).

All the C-S sets of informative CpG islands (iCpG) with a finite discretization threshold (computed as detailed in the SEP) were observed as hypermethylated in at least one cell line sample from the cancer type under consideration (according to the same threshold), but for LGG (83%) and PRAD (90%).

In many of the cases where cancer functional event (CFE) coverage in the cell line panel was modest, a CFE was either altered at a very low frequency in patient tumors of the relevant cancer type, or few cell lines of the relevant cancer type were available. For example, CGs absent in the corresponding cell lines had a median frequency of 1.30% in primary tumor samples (Table S2K) (min median = 0.35% for BRCA specific CGs missing in BRCA cell lines, max median = 11% for for DLBC specific CGs missing in DLBC cell lines) (Table S2K). Similarly, the majority of C-S RACSs absent in cell lines had a low alteration frequency in the primary tumors (median average alteration frequency across cancer types = 4.3%, min = 0.04% for LUAD, max = 7.11% for PRAD) (Figure S2B and Table S2K).
Furthermore, many CGs absent in the cell lines are associated with less strong evidence for positive selection (56% classed as Level C, 23% classed as Level B), with the median composition of the three confidence levels across cancer types = 54% C, 24% B, 23% A (Figure S2B and Table S2K).

B. Classes of cancer functional event and enrichment analysis

By applying the method described in (Ciriello et al., 2013) to our integrated dataset of tumor and cell lines, as described in the experimental procedures and the extended experimental procedures (SEP) 11, we identified 4 classes of Cancer Functional Events: M, CD, CA, and H.

Class M is enriched for Cancer Genes (CGs) mutations, classes CD and CA for recurrently altered chromosomal segments (RACSs) copy number alterations, and class H for hypermethylated informative CpG islands (iCpGs). All these feature types are defined in the experimental procedures and the SEP3, 5, and 7.

The M class represented 34% of samples (36% of the primary tumors and 28% of the cell lines). It was enriched for CG mutations (Fisher exact test (FET) p-value = 5.41×10^{-130}) and was dominated by cell lines and tumors from THCA (95% of the tumors and 63% of the cell lines), SKCM (73% of the tumors and 73% of the cell lines), and COAD/READ (80% of the tumors and 86% of the cell lines) (Figure 3C, Table S3D, and Table S3E).

When looking at enrichments of individual CFEs in class M (as detailed in the SEP12), we found that the top enriched 10 mutations involved: *BRAF*, *APC*, *PTEN*, *ANK3*, *KRAS*, *CTNNB1*, *NF1*, *ARID1A*, *ATM* and *MLL3* (Table S3F). Class M included, as individually enriched, deleted segments containing *ZNF3*, and *ARFGAP3* (Figure S3 and Table S3F) and hypermethylated CpG islands in the promoter regions of *DUSP22* and *ARL17A*.

Class CD represented 15% of the samples (15% of the tumors and 13% of the cell lines) and was enriched for RACSs copy number alterations (CNAs) (FET p-value = 5.12×10^{-36}), particularly for RACSs deletions (FET p-value = 2.04×10^{-15}). It was dominated by, consistently across cell lines and tumors, the LAML (68% of the tumors and 79% of the cell lines), KIRC (38% of the tumors and 53% of the cell lines), and GBM (70% of the tumors and 17% of the cell lines) samples, and partially covered PRAD, SKCM, and LUAD samples, consistently across cell lines and tumors (Figure 3C, Table S3D, and Table S3E).

The most significantly enriched RACS alterations in the CD class involved deletions of segments containing *RET*, *ANK3*, *PTEN*, *CUL2*, *ABL1*, *JAK1* and *MAP3K4*. Class CD included 5 individually enriched mutations involving, *NPM1*, *PBRM1*, *IDH2*, *IDH1*, and *U2AF1*. Thus making the AML samples highly representative of this class and its underlying signature. A small number of iCpG hypermethylations were enriched as well, for example in the promoters of *FAM115A* and *PIK3R1* (Figure S3 and Table S3F).

Class CA was enriched for copy number alteration CFEs (FET p-value = 2.95×10^{-12}) and represents, consistently across cell lines and tumors, most of the BRCA (99% of the tumors and 100% of the cell lines), HNSC (95% of the tumors and 100% of the cell lines), LUAD (51% of the tumors and 89% of the cell lines) and LUSC (61% of the tumors and 80% of the cell lines) (Figure 3C, Table S3D, and Table S3E).

Class CA was prominently enriched for amplified RACSs (FET p-value = 2.38×10^{-16}, Table S3H). Among these we found amplifications of regions containing *MYC* (the most significant amplified gene), *RAD21*, *PABPC1*, *FGFR1*, *PIK3CB*, *ERBB2*, and *KRAS*, and deletions of segments including *CDKN2A* and *TP53*; consistently with this, enriched hypermethylated iCpGs were found in the promoters of *CDKN2A* and *CDKL2* (among the others). 7 mutations were enriched at the individual level and included alterations in *TP53*, *PIK3CA*, *NOTCH1*, *GATA3*, *CASP8* and *NFE2L2* (Figure S3 and Table S3F).

Finally, the H class was enriched for hypermethylated iCpGs (p-value = 2.19×10^{-48}) and was exclusively composed by LGG samples (80% of the tumors and 59% of the cell lines), consistent with the hypermethylation phenotype of glioma (Turcan et al., 2012).

Using this classification of tumor samples, we observed a good concordance between the contributions of each our 4 classes to the considered 15 cancer types and that reported in the previous study: 70% of M-to-M and C-to-CA/CD matching classifications, including BLCA and LUAD partially belonging to M and C in both cases with ratios close to 50% (Ciriello et al., 2013). Additionally, in our case, a clear partition of the C class into two subclasses (CD and CA, respectively enriched for copy number deletions and amplifications) emerged unsupervised from the data and correctly covered samples from relevant cancer types. Finally, due to the inclusion of a larger epigenomic dataset, we were able to detect an additional class (enriched for hypermethylated CpG islands) correctly classifying most of the LGG samples to this.

Finally, we observed concordance using these 4 classes when applied to primary tumors and cell lines of the same tissue type (the predominant CFE type, i.e. mutations, copy number alterations and hypermethylation) is consistently detected for 100% of the cancer types. For 12 out of 15 cancer types, 80% are consistently classified even when distinguishing between CA (associated to amplifications) and CD (associated to deletions). Collectively, these data demonstrate that in addition to concordance between primary tumor samples and matched cell lines at the level of CFE frequencies, this is also evident when looking at more global signatures of functional events (Table S3G).

C. Validation of the LOBICO models on an independent dataset

LOBICO was successfully run for 83 out of the 86 compounds in common between our study and the CTRP (2nd version, (Seashore-Ludlow et al., 2015)) (see Supporting Experimental Procedure, paragraph 24). For the remaining 3 compounds, fewer than 5 cell lines were sensitive according to the automated procedure to binarize the IC_{50}s, and thus LOBICO analysis was not performed. Table S5G provides details of all models. For 80 of the 83 models the groups of predictive and sensitive cell lines contained at least 5 cell lines. The p-values for the t-tests on the GDSC IC_{50}s and CTRP AUCs for the 81 models were highly correlated (Figure S5E) (Pearson correlation: 0.96, p-value = $2.0x10^{-43}$). When the 10 rescreens are removed the correlation is similar (0.95), but with a higher, yet still very significant p-value of $4.8x10^{-37}$. Selection of significant t-test p-values using a Bonferroni corrected p-value of 0.01 as a cut-off showed significant overlap between GDSC and CTRP (Fisher exact test, p = $1.7x10^{-6}$). This value drops to p = $1.5x10^{-5}$ in case the rescreens were not considered.

We applied a further filter to focus on interesting results. Particularly, we considered the 44 'predictive' LOBICO models. These models have a p-value < 0.05 and FDR < 5% according to the permutation tests (See SEP Section 20). Note that these are different from the t-test p-values. Of these 44 models, 7 were single-predictor models and 37 were multi-predictor models according to CV. For 43 of these 44 models the groups of predictive and sensitive cell lines contained at least 5 cell lines. The t-test p-values for these 43 models for GDSC and CTRP are depicted in Figure S5F. Also from this figure, there is clearly a large agreement between the two studies; LOBICO models inferred on GDSC data show separation between drug response measurements both for the GDSC IC_{50}s as well as the CTRP AUCs. Specifically, 9 out of the 10 best GDSC models were also significant in CTRP. (Here, we used a P-value cutoff of a 0.023, i.e. 1/43, corresponding to a FWER of 1 and a corresponding FDR smaller than 1%, to decide upon significance). In total, there were 39 models that were statistically significant for the GDSC data, 17 (44%) of which also showed significance in the CTRP dataset. We have created violin plots with the underlying drug response data for these models (available at: http://www.cancerrxgene.org/gdsc1000/Logic_models.html).
