# Supplementary material for: A Landscape of Pharmacogenomic Interactions in Cancer
Source: Cell. 2016 Jul 28;166(3):740–54. doi: 10.1016/j.cell.2016.06.017 (PMC4967469; doi:10.1016/j.cell.2016.06.017)
Supplement: Document S2. Article plus Supplemental Information [file mmc9.pdf]

Cell

Resource

# A Landscape of Pharmacogenomic Interactions in Cancer

## Graphical Abstract

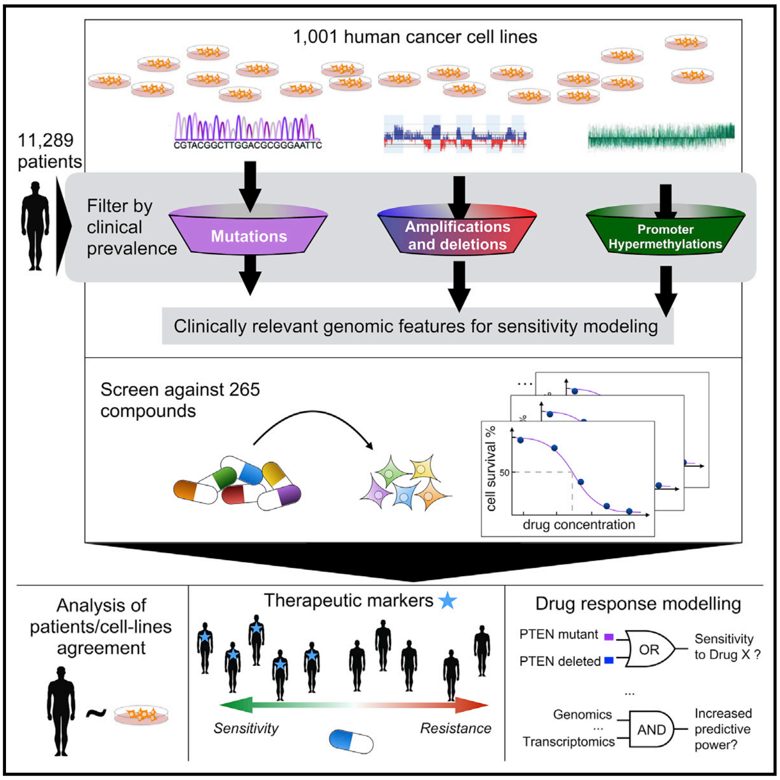


## Authors

Francesco Iorio, Theo A. Knijnenburg, Daniel J. Vis, ..., Julio Saez-Rodriguez, Ultan McDermott, Mathew J. Garnett

## Correspondence

um1@sanger.ac.uk (U.M.),
mg12@sanger.ac.uk (M.J.G.)

## In Brief

A look at the pharmacogenomic landscape of 1,001 human cancer cell lines points to new treatment applications for hundreds of known anti-cancer drugs.

## Highlights

- We integrate heterogeneous molecular data of 11,289 tumors and 1,001 cell lines
- We measure the response of 1,001 cancer cell lines to 265 anti-cancer drugs
- We uncover numerous oncogenic aberrations that sensitize to an anti-cancer drug
- Our study forms a resource to identify therapeutic options for cancer sub-populations

## Accession Numbers

GSE68379
E-MTAB-3610

Iorio et al., 2016, Cell *166*, 740–754
July 28, 2016 © 2016 The Author(s). Published by Elsevier Inc.
http://dx.doi.org/10.1016/j.cell.2016.06.017

# A Landscape of Pharmacogenomic Interactions in Cancer

Francesco Iorio,[1,2,20] Theo A. Knijnenburg,[3,4,20] Daniel J. Vis,[4,20] Graham R. Bignell,[2,20] Michael P. Menden,[1,5,20] Michael Schubert,[1] Nanne Aben,[4,6] Emanuel Gonçalves,[1] Syd Barthorpe,[2] Howard Lightfoot,[2] Thomas Cokelaer,[1,2,17] Patricia Greninger,[7] Ewald van Dyk,[4] Han Chang,[8] Heshani de Silva,[8] Holger Heyn,[9] Xianming Deng,[10,11,18] Regina K. Egan,[7] Qingsong Liu,[10,11] Tatiana Mironenko,[2] Xeni Mitropoulos,[7] Laura Richardson,[2] Jinhua Wang,[10,11] Tinghu Zhang,[10,11] Sebastian Moran,[9] Sergi Sayols,[9,19] Maryam Soleimani,[2] David Tamborero,[12] Nuria Lopez-Bigas,[12,13] Petra Ross-Macdonald,[8] Manel Esteller,[9,13,14] Nathanael S. Gray,[10,11] Daniel A. Haber,[7,15] Michael R. Stratton,[2] Cyril H. Benes,[7] Lodewyk F.A. Wessels,[4,6,16,21] Julio Saez-Rodriguez,[1,5,21] Ultan McDermott,[2,21,*] and Mathew J. Garnett[2,21,*]

[1]European Molecular Biology Laboratory, European Bioinformatics Institute, Wellcome Genome Campus, Cambridge CB10 1SA, UK
[2]Wellcome Trust Sanger Institute, Wellcome Genome Campus, Cambridge CB10 1SA, UK
[3]Institute for Systems Biology, Seattle, WA 98109, USA
[4]Division of Molecular Carcinogenesis, The Netherlands Cancer Institute, Amsterdam 1066 CX, The Netherlands
[5]Faculty of Medicine, Joint Research Centre for Computational Biomedicine, RWTH Aachen University, Aachen 52057, Germany
[6]Department of EEMCS, Delft University of Technology, Delft 2628 CD, the Netherlands
[7]Center for Cancer Research, Massachusetts General Hospital, Harvard Medical School, Charlestown, MA 02129, USA
[8]Genetically Defined Diseases and Genomics, Bristol-Myers Squibb Research and Development, Hopewell, NJ 08534, USA
[9]Cancer Epigenetics and Biology Program (PEBC), Bellvitge Biomedical Research Institute (IDIBELL), L'Hospitalet 08908, Barcelona, Catalonia, Spain
[10]Department of Cancer Biology, Dana-Farber Cancer Institute, Boston, MA 02215, USA
[11]Department of Biological Chemistry & Molecular Pharmacology, Harvard Medical School, Boston, MA 02215, USA
[12]Research Program on Biomedical Informatics, IMIM Hospital del Mar Medical Research Institute and Universitat Pompeu Fabra, Barcelona 08003, Spain
[13]Institució Catalana de Recerca i Estudis Avançats (ICREA), 08010 Barcelona, Catalonia, Spain
[14]Department of Physiological Sciences II of the School of Medicine, University of Barcelona, L'Hospitalet 08908, Barcelona, Catalonia, Spain
[15]Howard Hughes Medical Institute, Chevy Chase, MD 20815, USA
[16]Cancer Genomics Netherlands, Uppsalalaan 8, Utrecht 3584CT, the Netherlands
[17]Present address: Bioinformatics and Biostatistics Hub, C3BI, USR 3756 IP CNRS, Institut Pasteur, 75015 Paris, France
[18]Present address: Innovation Center for Cell Signaling Network, School of Life Sciences, Xiamen University, 361102 Xiamen, China
[19]Present address: Institute of Molecular Biology, Mainz 55128, Germany
[20]Co-first author
[21]Co-senior author
*Correspondence: um1@sanger.ac.uk (U.M.), mg12@sanger.ac.uk (M.J.G.)
http://dx.doi.org/10.1016/j.cell.2016.06.017

## SUMMARY

Systematic studies of cancer genomes have provided unprecedented insights into the molecular nature of cancer. Using this information to guide the development and application of therapies in the clinic is challenging. Here, we report how cancer-driven alterations identified in 11,289 tumors from 29 tissues (integrating somatic mutations, copy number alterations, DNA methylation, and gene expression) can be mapped onto 1,001 molecularly annotated human cancer cell lines and correlated with sensitivity to 265 drugs. We find that cell lines faithfully recapitulate oncogenic alterations identified in tumors, find that many of these associate with drug sensitivity/resistance, and highlight the importance of tissue lineage in mediating drug response. Logic-based modeling uncovers combinations of alterations that sensitize to drugs, while machine learning demonstrates the relative importance of different data types in predicting drug response. Our analysis and datasets are rich resources to link genotypes with cellular phenotypes and to identify therapeutic options for selected cancer sub-populations.

## INTRODUCTION

Cancers arise because of the acquisition of somatic alterations in their genomes that alter the function of key cancer genes (Stratton et al., 2009). A number of these alterations are implicated as determinants of treatment response in the clinic (Chapman et al., 2011; Mok et al., 2009; Shaw et al., 2013). Studies from The Cancer Genome Atlas (TCGA) and the International Cancer Genome Consortium (ICGC) have generated comprehensive catalogs of the cancer genes involved in tumorigenesis across a broad range of cancer types (Lawrence et al., 2014; Tamborero et al., 2013b; Zack et al., 2013). The emerging landscape of oncogenic alterations in cancer points to a hierarchy of likely functional processes and pathways that may guide the future treatment of

 © 2016 The Author(s). Published by Elsevier Inc.
This is an open access article under the CC BY license (http://creativecommons.org/licenses/by/4.0/).

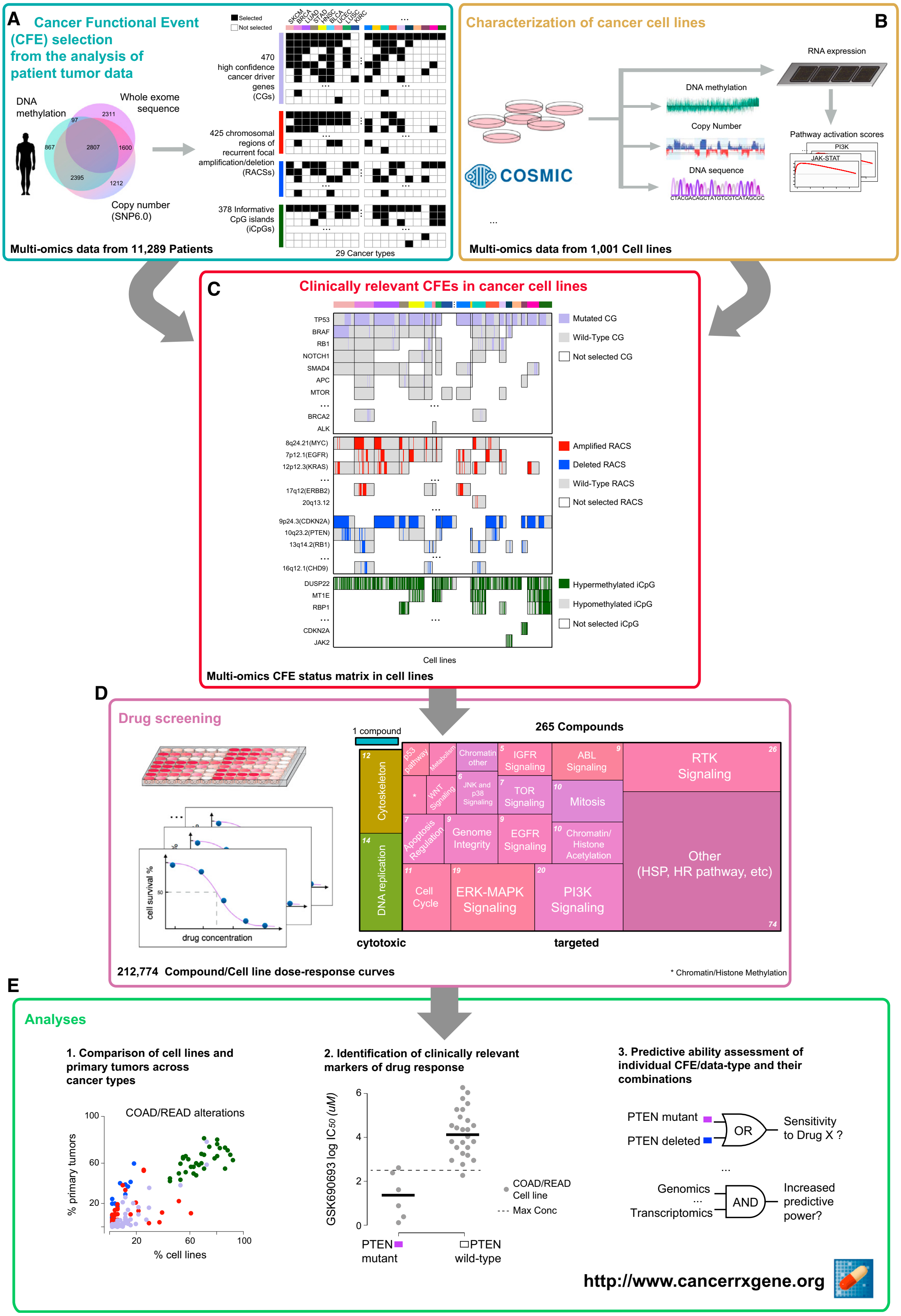


*(legend on next page)*

patients (Ciriello et al., 2013; Hanahan and Weinberg, 2000; Stratton et al., 2009).

Clinical trials are complex and expensive, and pre-clinical data that helps stratify patients can dramatically increase the likelihood of success during clinical development (Cook et al., 2014; Nelson et al., 2015). Thus, pre-clinical biological models that, as much as reasonably possible, capture both the molecular features of cancer and the diversity of therapeutic responses are a necessity. Human cancer cell lines are a facile experimental model and are widely used for drug development. Large-scale drug sensitivity screens in cancer cell lines have been used to identify clinically meaningful gene-drug interactions (Barretina et al., 2012; Basu et al., 2013; Garnett et al., 2012; Seashore-Ludlow et al., 2015). In the past, such screens have labored under the limitation of an imperfect understanding of the landscape of cancer driver genes, but it is now possible to view drug sensitivity in such models through the lens of clinically relevant oncogenic alterations.

Here, we analyzed somatic mutations, copy number alterations, and hypermethylation across a total of 11,289 tumor samples from 29 tumor types to define a clinically relevant catalog of recurrent mutated cancer genes, focal amplifications/deletions, and methylated gene promoters (Figure 1A; Tables S1A–S1D). These oncogenic alterations were investigated as possible predictors of differential drug sensitivity across 1,001 cancer cell lines (Figures 1B and 1C; Table S1E) screened with 265 anti-cancer compounds (Figures 1D and S1; Table S1F). We have carried out an exploration of these data to determine (1) the extent to which cancer cell lines recapitulate oncogenic alterations in primary tumors, (2) which oncogenic alterations associate with drug sensitivity, (3) whether logic combinations of multiple alterations better explain drug sensitivity, and (4) the relative contribution of different molecular data types, either individually or in combination, in predicting drug response (Figure 1E).

## RESULTS

### Oncogenic Alterations in Human Tumors

We built a comprehensive map of the oncogenic alterations in human tumors using data from TCGA, ICGC, and other studies (Figure 1A; Table S1C). The map consisted of (1) cancer genes (CGs) for which the mutation pattern in whole-exome sequencing (WES) data is consistent with positive selection, 2) focal recurrently aberrant copy number segments (RACSs) from SNP6 array profiles, and 3) hypermethylated informative 5′C-phosphate-G-3′ sites in gene promoters (iCpGs) from DNA methylation data, hereafter collectively referred to as "Cancer functional events" (CFEs). We identified CFEs by combining data across all tumors (pan-cancer), as well as for each cancer type (cancer specific) (Tables S2A, S2D, and S2H).

The WES dataset consisted of somatic variant calls from 48 studies of matched tumor-normal samples, comprising 6,815 samples and spanning 28 cancer types (Tables S1A–S1D). CGs were detected per cancer type by combining the outputs of three algorithms: MutSigCV, OncodriveFM, and OncodriveCLUST (Lawrence et al., 2013; Rubio-Perez et al., 2015; Tamborero et al., 2013a). This identified 461 unique pan-cancer genes (Table S2A). We further added nine genes identified as putative tumor suppressors (Wong et al., 2014). We mined the COSMIC database to identify likely driver mutations in 358 of the 470 CGs (Table S2B; Supplemental Experimental Procedures). Most tumors harbored only a few driver mutations (median n = 2, range 0–64), consistent with previous reports (Kandoth et al., 2013; Vogelstein et al., 2013).

RACSs were identified using ADMIRE for the analysis of 8,239 copy number arrays spanning 27 cancer types (van Dyk et al., 2013) (Table S1D; Supplemental Experimental Procedures). In total, 851 cancer-specific RACSs were gained (286 segments) or lost (565 segments), with a median of 19 RACSs per tumor type (Table S2D). The median number of genes within each RACS was 15 for amplified regions and one for deleted regions. The majority of known driver gene amplifications (e.g., *EGFR*, *ERBB2*, *MET*, and *MYC*) and homozygous deletions (e.g., *CDKN2A*, *PTEN*, and *RB1*) were captured, with 320 RACSs (38%) containing at least one known putative cancer driver gene, in addition to 531 RACSs (62%) without known driver genes. A smaller pan-cancer set (due to overlap in RACSs across cancer types) was constructed by pooling these results, comprising 425 RACSs (117 amplified and 308 deleted) (Tables S2D–S2F).

iCpGs were identified using DNA methylation array data for 6,166 tumor samples spanning 21 cancer types (Table S1D). We defined 378 iCpGs based on a multimodal distribution of their methylation signal in at least one cancer type (Tables S2H and S2I). This also established a discretization threshold used to define such regions as hyper-methylated in the cell lines (Table S2J; Supplemental Experimental Procedures).

In total, our multidimensional analysis of >11,000 patient tumor samples identified 1,699 cancer-specific CFEs, which were further merged into 1,273 unique pan-cancer CFEs (Figure S2A).

### *Oncogenic Alterations in Patient Tumors Are Conserved across Cell Lines*

Next, we assessed the extent to which the mutational landscape of cancer cell lines captures that seen in primary tumors. We utilized a panel of 1,001 human cancer cell lines analyzed through WES (n = 1,001), copy number (n = 996), gene expression

**Figure 1. Overview of Data and Analyses**

(A) Publicly available genomic data for a large cohort of primary tumors were analyzed to identify clinically relevant features called cancer functional events.

(B) A panel of 1,001 genomically characterized human cancer cell lines.

(C) The catalog of CFEs from patient tumors was used to filter the set of molecular alterations identified in cell lines and subsequently was used for pharmacogenomic modeling.

(D) Cancer cell lines were screened for differential sensitivity against 265 anti-cancer compounds.

(E) The resultant datasets were used for pharmacogenomic modeling.

See also Figure S1 and Table S1.

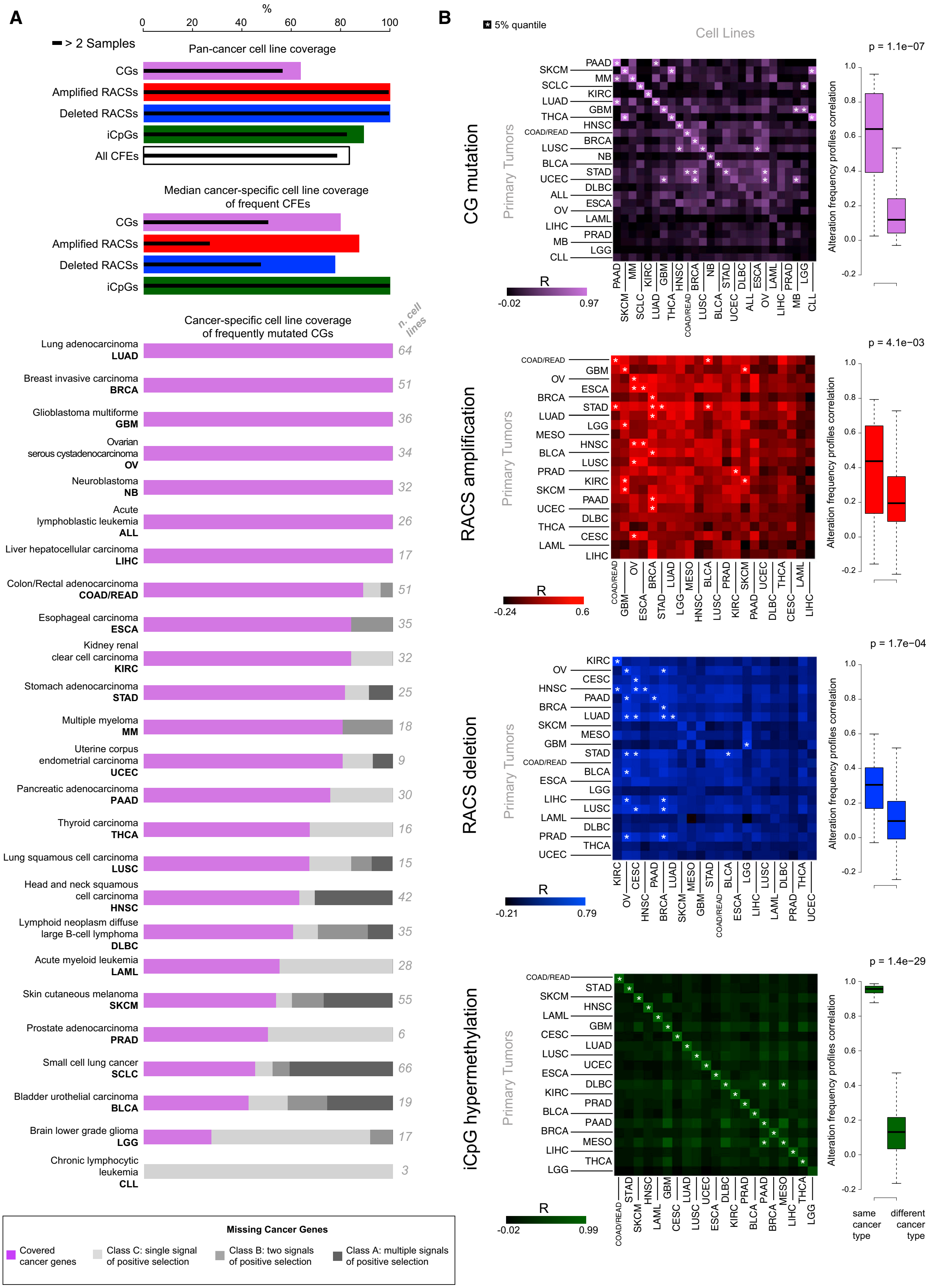


(legend on next page)

(n = 968), and DNA methylation (n = 957) (http://cancer.sanger.ac.uk/cell_lines) (Figure 1B) and which we reclassified according to the TCGA tissue labels (Figure 2A; Tables S1A and S1E). Molecular alterations identified in cell lines were filtered using the CFEs identified in the primary tumor samples, providing a set of clinically relevant CFEs for the cell lines (Figure 1C).

Of the 1,273 pan-cancer CFEs identified in patient tumors, 1,063 (84%) occurred in at least one cell line, and 1,002 (79%) occurred in at least three (Figure 2A). This concordance was greatest for the RACSs (100% of 425; Table S2G), followed by iCpGs (338 of 378, 89%; Table S2J) and CGs (300 of 470, 64%; Table S2C). When considering cancer-specific CFEs, concordance was highest for CFEs occurring in at least 5% of patients (median of 86% of CFEs covered across cancer types; Figure 2A; Data S1A). Coverage of CFEs varied by cancer type, and when we include infrequent CFEs (occurring in < 5% of patients), this concordance is markedly lower for the majority of cancer types (median coverage = 46%; Figure S2B). CFEs absent in cell lines are reported in Table S2K.

The correlation between the frequency of CFEs in cell lines and patient tumors was high for the majority of the cancer types and for all three classes of CFEs (Figures 2B and S2C; Table S2L; Supplemental Experimental Procedures). Using a simple nearest-neighbor classifier based on the presence of CFEs in cell lines and tumors across cancer types, we could correctly match the tissue of origin of cell lines to primary tumors (and vice versa) for 71% of the cases (27 out of 38 alteration profiles [randomly expected 1%]) (Figures S2D and S2E; Table S2M; Supplemental Experimental Procedures). This percentage increased to 81% and 92% (randomly expected 2% and 5%), when considering the second and fifth nearest-neighbors, respectively (Figure S2E).

The frequency of alterations in 13 canonical cancer-associated pathways was highly correlated between cell lines and tumors of the same cancer type (median $R = 0.75$ across all 13 pathways) (Figure 3A; Table S3A).

A previous hierarchical classification of ~3,000 tumors identified two major subclasses: M and C class (dominated by mutations and copy number alterations, respectively) (Ciriello et al., 2013). We expanded this analysis by including methylation data and by jointly analyzing cell lines and tumor samples. This integrated analysis of 3,673 samples (composed of 1,001 cell lines and 2,672 primary tumors for which all three data types were available and that were positive for at least one of the 1,250 CFEs [Tables S3B and S3C]) yielded four classes referred to as M, H, CD, and CA (Table S3D; Supplemental Experimental Procedures). Class M is enriched for CG mutations, class H for hypermethylation of iCpGs, and classes CD and CA for deleted and amplified RACSs, respectively (Figures 3B and S3; Tables S3E, S3F, and S3H; Data S1B). We observed a high concordance between the predominant class of CFEs in primary tumors and cell lines of the same tissue type (80% of cancer types, exceptions being GBM, KIRC, and PRAD) (Figure 3C; Table S3G; Data S1B).

Taken together, these results show that a sufficiently large panel of cell lines is able to capture individual clinically relevant genomic alterations, in addition to pathway alterations and global signatures of driver events.

## A Therapeutic Landscape of Human Cancers

### *Modeling Pharmacogenomic Interactions*

To investigate how CFEs detected in primary tumors impact drug response, we first mapped these on our panel of cell lines (Figure 1C; Tables S2C, S2G, and S2J). Cell lines underwent extensive drug sensitivity profiling, screening 265 drugs across 990 cancer cell lines and generating 212,774 dose response curves (median number of screened cell lines per drug = 878, range = [366, 935]; Figure 1D). This is an expansion on previous pharmacogenomic datasets (Barretina et al., 2012; Basu et al., 2013; Garnett et al., 2012; Seashore-Ludlow et al., 2015). The effect of each drug on cell number was used to model sensitivity as $IC_{50}$ (drug concentration that reduces viability by 50%) or AUC (area under the dose-response curve) values (Tables S4A and S4B).

Screened compounds included cytotoxics (n = 19) and targeted agents (n = 242) selected against 20 key pathways and cellular processes in cancer biology (Figure 1D; Table S1F). These 265 compounds include clinical drugs (n = 48), drugs currently in clinical development (n = 76), and experimental compounds (n = 141). We screened seven compounds as biological replicates and observed good correlation between replicate $IC_{50}$ values with a median Pearson correlation (R) = 0.65 (0.78 for the compounds with most of $IC_{50}$ values falling within the range of tested concentrations) and consistent classification of cell lines as sensitive or resistant to a compound (median Fisher's exact test [FET] $\log_{10}$ p value = −26) (Figure S1). Cluster analysis based on AUC values confirmed that compounds with overlapping nominal targets or targeting the same process/pathway had similar activity profiles (Table S1G; Supplemental Experimental Procedures).

We used three distinct analytical frameworks to define the contribution of CFEs to the prediction of drug sensitivity (Figure 1E). ANOVA was used to identify single CFEs as markers of drug response. Logic models identified combinations of CFEs that improve the prediction of drug response. Lastly, we used machine-learning algorithms to assess the contribution of

**Figure 2. Representation of Cancer Functional Events in Cancer Cell Lines**

(A) First bar chart: the percentage coverage of cancer functional events (CFEs) in the pan-cancer dataset occurring in at least one cell line. Coverage for each class of CFEs individually and when combined is shown. Second bar chart: the median coverage by cancer type of frequently occurring (>5% of tumor samples) cancer-specific CFEs in at least one cell line. The solid line indicates coverage of CFEs occurring in >2 cell lines. Third bar chart: coverage in each cancer type of frequently occurring cancer genes (CGs). Missing cancer genes are grouped by the level of evidence supporting their classification as a cancer gene. The number of cell lines for each cancer-type and the full name of each cancer-type and associated acronym are shown.

(B) Matrix of Pearson correlations of CFE frequency between cell lines and patient tumors for each cancer-type and class of CFEs. Box and whisker plots show the correlations of CFEs within the same (on-diagonal) and between different (off-diagonal) cancer-types.

See also Figure S2, Table S2, and Data S1.

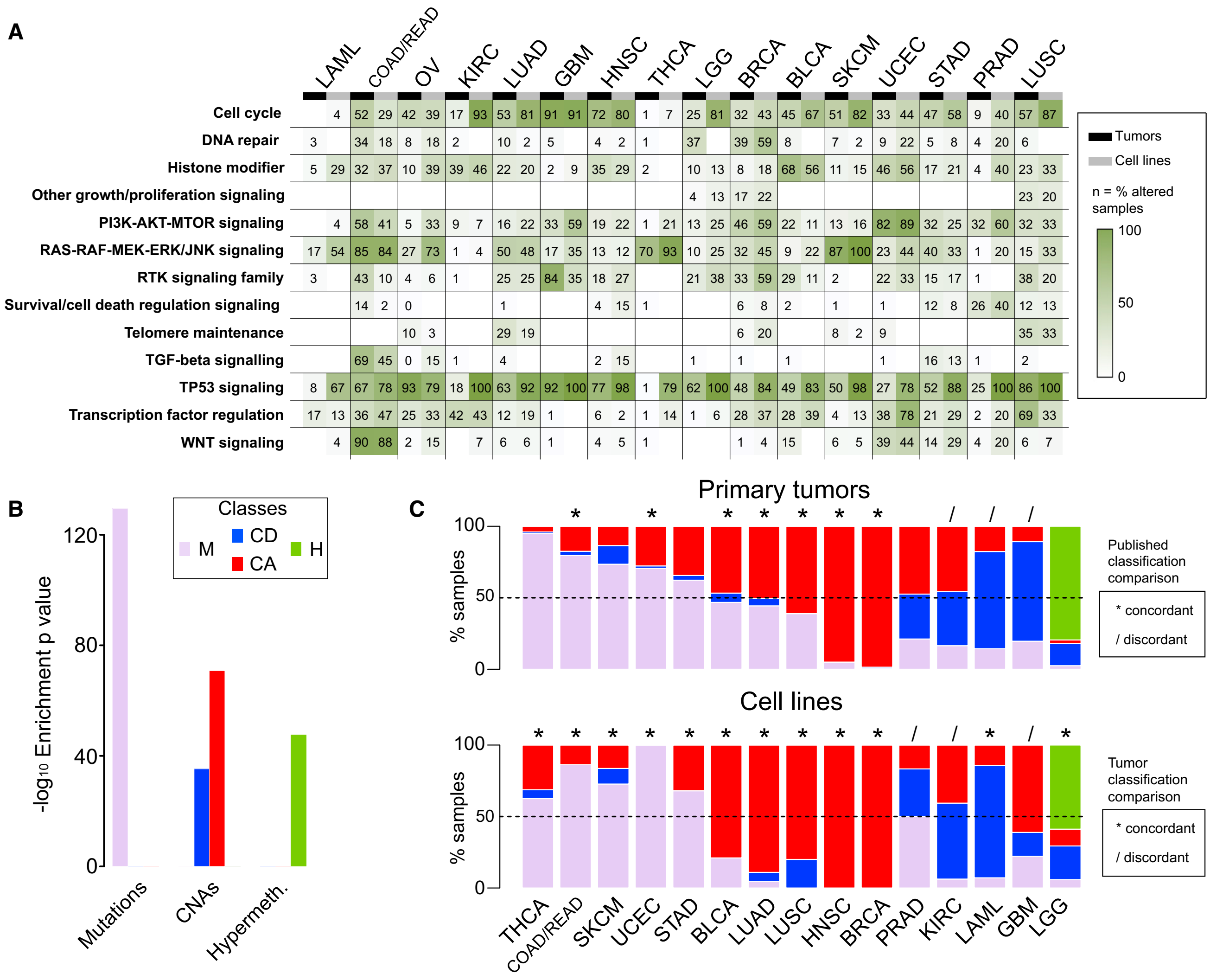


**Figure 3. Comparative Analysis of Pathway Alterations and Global CFE Signatures in Cell Lines and Tumors**

(A) Concordance of CFEs in cancer-associated pathways between cell lines and tumors.

(B) Enrichments of the dominant CFE type across four global classes.

(C) Classification of primary tumors and cell lines from each cancer type into global classes based on CFEs. Segment lengths are the percentage of samples (cell lines or primary tumors) falling within each global class. For primary tumors, results are compared to published classifications (Ciriello et al., 2013) (top diagram), and for cell lines, the comparison is with primary tumors from the same cancer type (bottom diagram). The classification of concordance is based on the identity of the predominant class of CFEs.

See also Figure S3, Table S3, and Data S1.

each molecular data type (CGs, RACS, iCpGs, and gene expression) in explaining variation in drug response. For consistency, all analyses used $IC_{50}$ values. We carried out a pan-cancer, as well as a cancer-specific, analysis (for those 18 cancer types of sufficient sample size, n > 15 cell lines).

### *ANOVA Analysis Defines a Landscape of Pharmacogenomic Interactions*

For pan-cancer ANOVA, the set of CFEs included 267 CGs, 407 RACSs, and three gene fusions (*BCR-ABL*, *EWSR1-FLI1*, and *EWSR1-X*). Overall, for the 265 compounds, we identified 688 statistically significant interactions between unique CFE-drug pairs (p value $< 10^{-3}$ at a false discovery rate [FDR] < 25%; Figure 4A), with 540 pan-cancer and 174 cancer-specific hits (Figure S4A; Table S4C). A subset of 262 CFE-drug pairs was additionally defined as large-effect interactions (Figure 4A). The effect size was quantified through Glass deltas (Δs) and Cohen's D (CD) (Supplemental Experimental Procedures).

The majority of CFE-drug interactions was exclusively identified in either the pan-cancer or cancer-specific analysis (n = 662 of 688 significant interactions, 96%, and n = 254 of 262 significant large-effect interactions, 97%), with few overlapping interactions (Figure 4A; Table S4C). The effect size was frequently greater for the cancer-specific associations than for pan-cancer associations (CD > 1 for 100% and 30% of hits,

respectively) (Table S4D). A possible explanation for this observation could be that cancer-specific associations, with fewer cell lines, require a larger effect size to be statistically significant. However, downsampled pan-cancer analyses confirmed that the increased effect size of cancer-specific associations is greater than expected by downsampling alone (Figures S4B and S4C; Supplemental Experimental Procedures). This indicates that sensitivity to many drugs is modulated by genomic alterations in the context of a defined tissue lineage.

Overall, 233 of 674 (34%) CFEs were significantly associated with the response to at least one compound, and more RACSs (62%) were associated with response than were CGs (38%). The importance of these two classes of CFEs varied by cancer type and was related to their prevalence (Figures 3C and S4G).

We identified significant associations for the majority of compounds (85%; n = 225 of 265). When compounds were classified by their nominal target into 20 specific biological processes (Figure S4H; Table S1F), CFEs best explained sensitivity to compounds targeting EGFR and ABL signaling, mitosis, and DNA replication and least explained sensitivity to compounds targeting TOR, IGF1R, and WNT signaling. For the latter, alternative non-genomic events may be the primary modulators of drug sensitivity. The proportion of cytotoxic and targeted compounds (Table S1F) associated with at least one significant large-effect interaction was similar (63% and 60%, respectively). However, compared to targeted agents, the significant interactions between CFEs and cytotoxics tended to be of a smaller effect size (average CD 0.96 vs. 1.32) and less significant (average $-\log_{10}$ p value 3.68 vs. 4.56).

We performed ANOVA on randomly downsampled subsets of cell lines (500, 300, 150, and 60 cell lines) and evaluated our ability to retain the set of statistically significant associations. The number of associations exponentially decreased as the number of cell lines was reduced, with a loss of ~80% of pan-cancer associations when using 500 cell lines (Figures S4D–S4F; Supplemental Experimental Procedures). This highlights the utility of using a large cell line collection to increase statistical power and to preserve representation of diverse genotypes and histologies.

### ANOVA Identifies Known and Novel Gene-Drug Associations

Among the individual CFE-drug associations, we identified many well-described pharmacogenomics relationships (Figure 4B). These included clinically relevant associations between alterations in *BRAF*, *ERBB2*, *EGFR*, and the *BCR-ABL* fusion gene and sensitivity to clinically approved drugs in defined tumor types, as well as associations between *KRAS*, *PDGFR*, *PIK3CA*, *PTEN*, *CDKN2A*, *NRAS*, *TP53*, and *FLT3* with drugs that target their respective protein products or pathways (Figure 4B; Table S4C). Moreover, we observed a secondary T790M *EGFR* mutation in lung adenocarcinoma (LUAD) and resistance to EGFR-targeted therapies (Gefitinib and Afatinib) (Godin-Heymann et al., 2008) (Figure 4D), as well as resistance of *NRAS* mutated melanoma patients to a BRAF inhibitor (Figure 4B; Table S4C) (Su et al., 2012).

A pathway-centric view highlighted the number of interactions between CFEs in cancer pathways (EGFR, ERK-MAPK, PI3K-MTOR, and DNA repair and cell-cycle-related pathways) and drugs targeting those CFEs (Figure S4I). For example, compounds targeting EGFR signaling showed potent activity in cells with *EGFR* and *ERBB2* alterations, but were ineffective in cells with downstream alterations in ERK-MAPK signaling, such as mutant *RAS*.

To explore the most important CFE-drug interactions, we focused on 262 associations with a large effect on drug sensitivity ($p < 10^{-3}$, FDR < 25%, and $\Delta > 1$, for both the cell line populations included in the test) (Figure 4C; Table S4C). For example, at the pan-cancer level, *U2AF1* mutations associate with sensitivity to multiple FLT3 inhibitors, such as AC220 ($p = 8.3 \times 10^{-8}$, CD = 2.5), Sorafenib ($p = 3.04 \times 10^{-6}$, CD = 2.8), Sunitinib ($p = 5.6 \times 10^{-5}$, CD = 2.5), and XL-184 ($p = 1.3 \times 10^{-4}$, CD = 1.9); *PTEN* mutations associate with sensitivity to an AKT inhibitor in COAD/READ ($p = 3.5 \times 10^{-6}$, CD = 2.4). The chemotherapeutic Mitomycin C is widely used to treat BLCA, and here, we detect, in the BLCA specific analysis, a sensitizing interaction with mutations in *TP53* ($p = 9.9 \times 10^{-5}$, CD = 2.8) that are highly prevalent in this cancer type. In LUSC cells, loss-of-function mutations in the DNA methyltransferase *MLL2* are associated with sensitivity to the clinical anti-androgen Bicalutamide ($p = 6.02 \sim 10^{-4}$, CD = 3); the BCL-2 inhibitor, ABT-263, shows activity in COAD/READ cells that harbor focal amplifications of *MET* ($p = 1.02 \times 10^{-4}$, CD = 2.8) or *FOXA1/CRNKL1* ($p = 1.31 \times 10^{-4}$, CD = 2.2), events found in almost 60% of colorectal tumors; and truncating mutations in the co-repressor of BCL6, *BCOR*, statistically interact ($p = 2.04 \times 10^{-5}$, CD = 3.5) with sensitivity to a PKC beta inhibitor in STAD (Figure 4D), and deletions of a RACS (2q37.3) containing *MTERFD2* and *SNED1* is associated with resistance to the HDAC inhibitor Vorinostat ($p = 5.4 \times 10^{-7}$, CD = 4; Figure 4D) in OV cell lines.

Interestingly, 24 of the 262 associations are driven by RACSs that do not contain known cancer genes (Tables S4C and S2D). For these regions, the patterns of drug sensitivity may give clues as to the likely contained driver cancer gene(s).

### Logic Formulas of Drug Response Refine Pharmacogenomic Modeling

Many genomic alterations occur together or in a mutually exclusive way that suggests a biological function (Babur et al., 2015). We hypothesized that combinations of CFEs could, in some contexts, improve our ability to explain variation in drug sensitivity. We employed a computational approach termed "logic optimization for binary input to continuous output" (LOBICO) to find the optimal logic model combining CFEs to explain the $IC_{50}$ values for a drug, for example, "if *RAS* or *RAF* mutated, then sensitive to MEK inhibition" (Knijnenburg et al., 2016). LOBICO binarizes the $IC_{50}$s, labeling cell lines as sensitive or resistant, and uses these together with the continuous $IC_{50}$s to find optimal models (Table S5C) (Supplemental Experimental Procedures). We employed 5-fold cross-validation (CV) to select the appropriate model complexity from a set of eight possible models, ranging from single CFE predictor models to complex multi-input models with up to four CFEs. We required solutions to have specificity greater than 80%. The input features included the CGs, RACSs, gene fusions, and binarized pathway activity scores derived from the basal gene expression profiles of the cell lines (Figure S5A; Tables S5A, S5B, and S5D). The latter is based on 11 transcriptional signatures of pathway activation (Parikh et al., 2010) (Table S5B; Supplemental Experimental

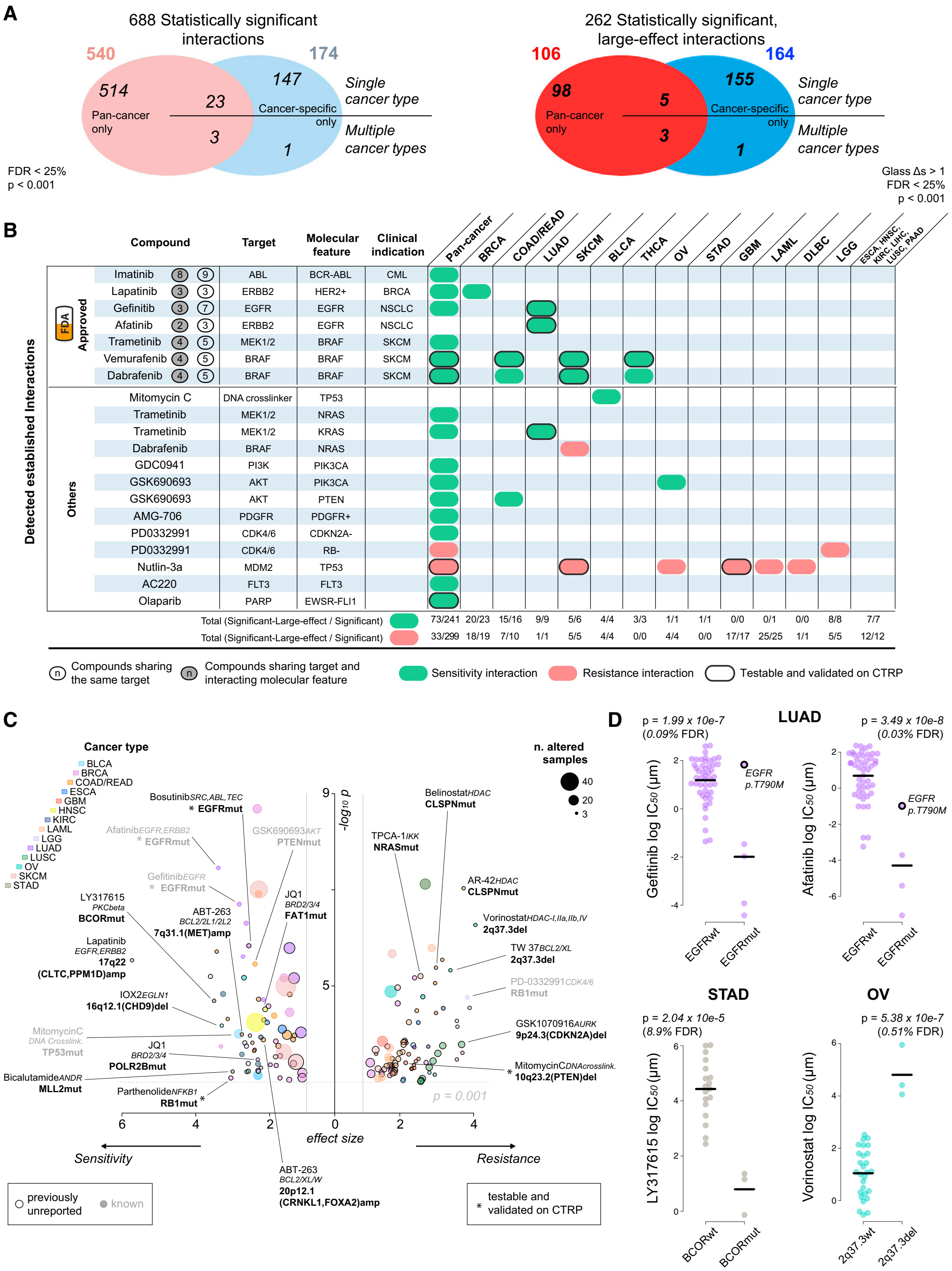


**Figure 4. Pharmacogenomic Modeling of Drug Sensitivity**

(A) Pan-cancer and cancer-specific ANOVA analyses for statistically significant interactions between differential drug sensitivity and CFEs. Cancer-specific interactions are divided into those identified in a single or multiple cancer-specific analyses.

(B) A summary of established pharmacogenomic interactions detected in this analysis including a subset of clinically approved markers. The total number of significant and significant large-effect interactions for each cancer type is provided. Testable interactions that were validated on the CTRP datasets are also indicated.

*(legend continued on next page)*

Procedures). LOBICO was executed for each drug separately utilizing pan-cancer and cancer-specific molecular datasets. This led to the inference of 1,112 logic models (Table S5E).

In the pan-cancer dataset we found that for 69% (182 of 265) of the drugs, the $IC_{50}$s were better explained than expected by chance (p value < 0.05 and FDR < 5%). Across the cancer-specific datasets, on average, 24% of the drugs were explained by the inferred logic models (Figure 5A). We termed these logic models (182 from the pan-cancer dataset and 208 from the 18 cancer-specific datasets) "predictive models". When considered together, the pan-cancer and cancer-specific LOBICO analyses identified predictive models for 208 out of 265 (78%) drugs. Importantly, for 85% of the 390 predictive models, a multi-input model achieved better performance than did the best single-predictor model (Figure 5B). Although the pan-cancer dataset produced the largest number of predictive models, the CV error was consistently higher than for cancer-specific datasets (Figure S5B). This is in agreement with the ANOVA analysis, where larger effect sizes were observed for the cancer-specific datasets. The response to drugs that target the p53 or ERK-MAPK pathway were especially well-predicted by LOBICO (Figure S5C).

We observed that CGs had the largest role in explaining drug response, followed by RACSs and the pathway activities derived from gene expression (Figure S5A; Supplemental Experimental Procedures). The small number of pathway signatures had a disproportionately large effect in the logic models, showing that basal pathway activation scores provide relevant information to predict drug response beyond the genomic CFEs (Costello et al., 2014) (Figure S5D).

LOBICO uncovered many known, as well as novel, associations (Table S5F). Figure 5C depicts a selection of particularly strong and consistent "and/or" combinations found for clinically approved drugs. For example, in the pan-cancer dataset, the "or" combination of *KRAS* or *BRAF* improved the precision and recall compared to single predictor models to explain cell line sensitivity to a number of MEK and RAF inhibitors (e.g., Trametinib in Figures 5C and 5D).

In general, the "or" combinations led to models with higher recall (Figure 5C, right quadrants) as compared with the single-predictor model. For example, HNSC cell lines that have an *EGFR* amplification or a *SMAD4* mutation account for 45% (10 out of 22) of cell lines sensitive to the ERRB2/EGFR inhibitor Afatinib, whereas considering only the *EGFR* amplified cell lines accounts for only 32% (7 out of 22) of the sensitive cell lines (Figure 5E). Conversely, "and" combinations led to models with higher precision (Figure 5C, left quadrants). For example, BRCA cell lines that lack a deletion of the *FAT1*/*IRF2* locus and are *TP53* mutant show increased sensitivity to the ERRB2/EGFR inhibitor Lapatinib. This is achieved at higher precision (57% instead of 45% for the single predictor model), but at a lower recall (80% instead of 100%) (Figure 5F). Collectively, LOBICO analysis highlights the importance of considering combinations of oncogenic alterations as biomarkers for drug response.

### Validation of Pharmacogenomic Modeling Results on Independent Datasets

We sought to validate our pharmacogenomic models using independent drug sensitivity datasets from the Cancer Cell Line Encyclopedia (CCLE) (Barretina et al., 2012) and the Cancer Therapeutics Response Portal (CTRP; second version) (Seashore-Ludlow et al., 2015). This analysis was for necessity restricted to only those compounds and cell lines shared with our own study (hereafter referred to as GDSC). The shared set consisted of 466 cell lines and 76 compounds from the CTRP study (Tables S4I–S4K) and 389 cell lines and 15 compounds from the CCLE study (Tables S4E–S4G; Supplemental Experimental Procedures). Validation was performed using $IC_{50}$ values from the GDSC and CCLE studies and AUC values from the CTRP study (where $IC_{50}$ values were not reported).

We performed ANOVA on the overlapping set of cell lines/compounds. We validated 53% (19 of 36 on CTRP) and 86% (6 of 7 on CCLE) of the testable sensitivity associations identified in the GDSC, and 21% (6 of 29 on CTRP) and 0% (0 of 7 on CCLE) of testable resistance associations ($p < 0.05$, Fisher's exact test CTRP: $p = 8.1 \times 10^{-9}$; CCLE: $p = 0.01$; Figures S4J and S4K; Tables S4H and S4L; Supplemental Experimental Procedures). A significant Pearson correlation of the CFE-drug interaction significance was observed between the GDSC dataset and the other two datasets (R = 0.86 for CTRP and R = 0.86 for CCLE; Figures S4J and S4K). Similarly, using LOBICO, we validated 44% (17 of 39) of testable models using the CTRP, including both single and multi-input models, and observed a significant Pearson correlation of the interaction significance between the two datasets (R = 0.96; Figures S5E and S5F; Data S1C). Thus, even within the relatively limited set of overlapping drugs and cell lines, resulting in reduced statistical power, we observed reasonable-to-good rates of validation for the set of pharmacogenomic interactions identified in our study, including a number of novel associations. Complete summaries of these comparisons are provided in Tables S4E–S4L and S5G, Data S1C, and Supplemental Experimental Procedures.

### Contribution of Different Molecular Data Types in Predicting Drug Response

To investigate the power of different combinations of molecular data to predict drug response, we built linear and non-linear models of drug sensitivity (elastic net [EN] regression and Random Forests [Costello et al., 2014]). As input features, we used CGs, RACSs, iCpGs, and gene expression data.

Here, we refer to EN models using $IC_{50}$ values (Table S4A), but very similar results were obtained with Random Forests (Figure S6F; Table S6A). We assessed the predictive power of

(C) Volcano plot with effect size (x axis) and significance (y axis) of large-effect cancer-specific pharmacogenomic interactions. Each circle corresponds to a significant CFE-drug interaction. Circle size is proportional to the number of altered cell lines, and the color indicates cancer type. A subset of interactions is labeled with drug name, target (italics), and name of the associated CFE (bold).

(D) Examples of cancer-specific pharmacogenomic interactions identified by our systematic ANOVA. Each circle represents the $IC_{50}$ of an individual cell line. The co-incident resistance-associated EGFR T790M mutation is labeled.

See also Figure S4 and Table S4.

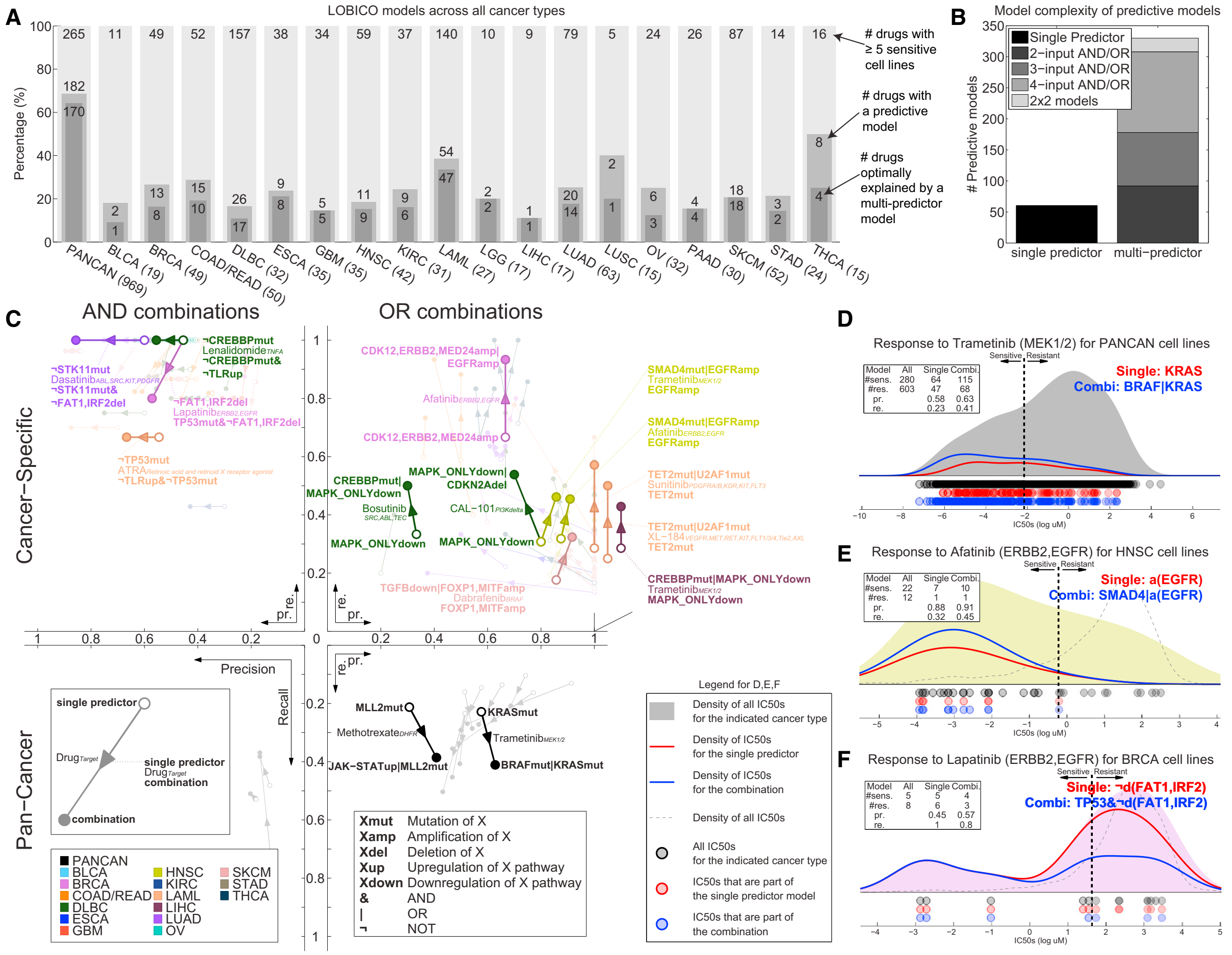


**Figure 5. Logic Models of CFEs Explain Drug Sensitivity**

(A) The number of predictive LOBICO models from the pan-cancer and cancer-specific analyses. The number of cell lines for each cancer type is given in brackets.

(B) Optimal model complexity for each of the predictive logic models.

(C) Strong AND/OR model combinations involving clinically approved drugs from the pan-cancer and cancer-specific analyses. Each arrow goes from the precision (x axis) and recall (y axis) of the single-predictor model to that of the logic combination. The arrow color reflects cancer type, and drug names and nominal targets (italics) are shown.

(D) Distribution of $IC_{50}$ values of all cell lines (gray) in response to Trametinib with respect to the KRAS mutant single-predictor model (red line) and the KRAS OR BRAF mutant combination (blue line). The dashed line is the $IC_{50}$ threshold used to classify cell lines as sensitive and resistant. The inset table shows the number of cell lines classified as sensitive or resistant for each model and the associated precision (pr.) and recall (re.).

(E) HNSC cell lines response to Afatinib with respect to EGFR amplification and the combination of EGFR amplification OR a SMAD4 mutation.

(F) BRCA cell lines response to Lapatinib with respect to lack of the FAT1/IRF2 deletion and the logical TP53 mutant AND lack of the FAT1/IRF2 deletion combination.

See also Figure S5, Table S5, and Data S1.

each model using the Pearson correlation coefficient ($R$) of observed versus predicted $IC_{50}$ values. For each of the 265 compounds, we built pan-cancer and cancer-specific models (for 18 cancer types) and considered a model with a corresponding $R_{\text{pan-cancer}} \geq 0.21$ and $R_{\text{cancer-specific}} \geq 0.25$ as predictive (Figures S6G and S6H; Supplemental Experimental Procedures).

In a pan-cancer analysis, the most predictive data type was gene expression, closely followed by the tissue of origin of the cell lines (Figure 6A). By comparison, genomic features (CG mutations and RACSs alterations) performed poorly. The predictive power of gene expression and tissue type was strongly correlated, while RACSs and CGs are less correlated with the tissue type (Figure S6A). This is consistent with the tissue specificity of gene expression (Ross et al., 2000).

Next, we compared the most predictive data types in pan-cancer versus cancer-specific analyses (Figures 6B and 6C). For

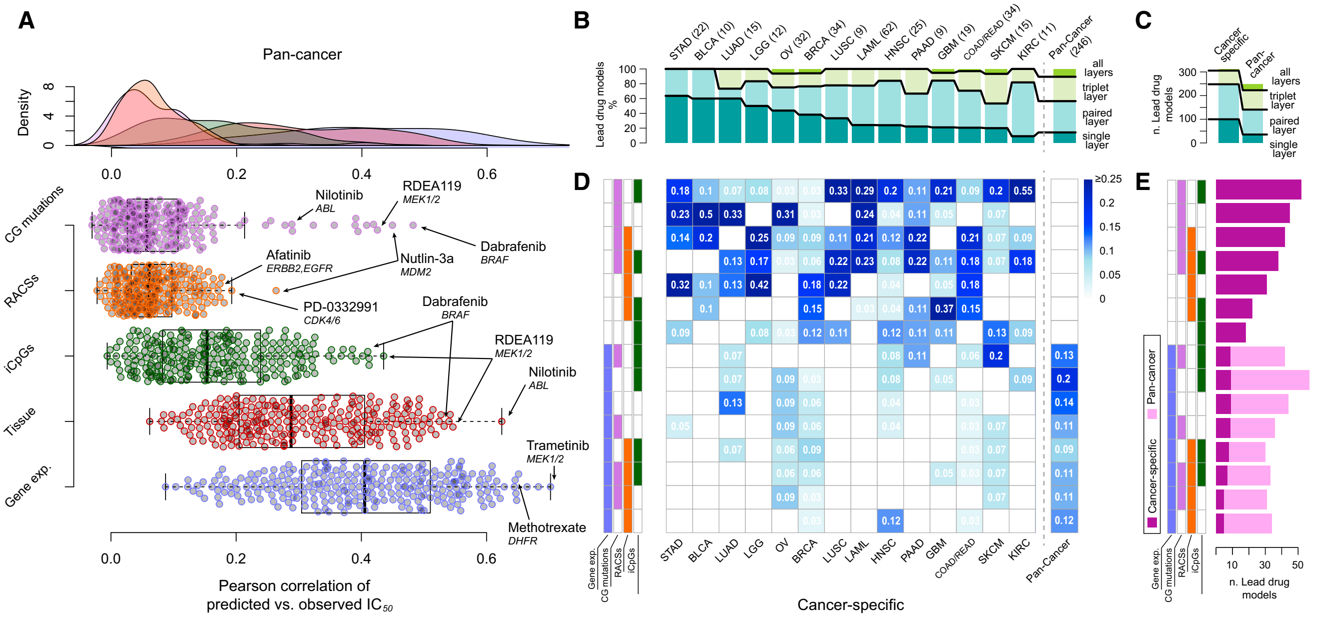


**Figure 6. Predictive Ability of Combinations of Molecular Data Types**

(A) Predictive performances of individual pan-cancer pharmacogenomic models using elastic net modeling and the indicated single data types. Selected outlier predictive models are labeled.

(B) The number of molecular data types included in the best-performing models (lead models) across the pan-cancer and cancer-specific analyses. The best-performing models use combinations of multiple data types. Absolute counts of best performing models are given.

(C) Absolute counts of lead models from the pan-cancer and cancer-specific analyses and the number of molecular data types used in the models.

(D) A heat map of the percentage of lead models identified in the pan-cancer and cancer-specific analyses incorporating different combinations of molecular data types.

(E) Absolute count of lead models identified in pan-cancer and cancer-specific analyses incorporating different combinations of molecular data types. Data types are ordered from most (top) to least (bottom) predictive in the cancer-specific analysis.

See also Figure S6 and Table S6.

each drug, we identified the best-performing combination of data types and the corresponding model, referred to as the “lead model”. Notably, paired molecular data types contributed to the most lead models in both the pan-cancer (~42% of all models) and the cancer-specific analyses (~45% for all cancer types) (Figures 4B and 4C). In the pan-cancer analysis, all of the lead models use gene expression data (Figures 6D and 6E), but for 211 drugs (~86%), the models are improved by including methylation, RACSs, CGs, or any combination of those additional data types. In addition, we identified 379 predictive (non-lead) models (~17%) independent of gene expression (Figures S6B–S6E).

In a cancer-specific analysis, the majority of lead models are based solely on genomics features (Figures 6D and 6E). For 120 cases (~38%) the lead model is based on genomics alone (CGs and RACS). We found that genomics in combination with methylation provided an additional 117 lead models (~37%), whereas genomics in combination with gene expression contributed 19 (~6%). The remaining lead models use methylation alone (~7%), gene expression alone (~3%), or a combination of genomic, epigenetic, and transcriptomic features (12%). Therefore, in the context of a cancer-specific analysis, ~74% (237 of 319) of lead models were explained by genomics, either alone or when combined with methylation (Figures 6D and 6E).

## DISCUSSION

### Constructing a Pharmacogenomics Resource

Cancer cell lines are important tools for drug development. Here, we have extended previous efforts with the systematic expansion of the pharmacological, genomic, transcriptomic, and epigenetic characterization of 1,001 human cancer cell lines. These datasets can be investigated through the COSMIC and Genomics of Drug Sensitivity in Cancer Web portal (http://www.cancerrxgene.org). To the best of our knowledge, this is the largest and most extensively characterized panel of cancer cell lines and should enable a broad range of studies linking genotypes with cellular phenotypes.

Our analysis of >11,000 patient tumor samples and the subsequent superimposing of salient cancer features on cell lines exemplifies how large-scale cancer sequencing can be used to empower biological research and maximizes the potential clinical relevance of the pharmacological models reported.

The majority of CFEs identified from a broad range of tumor types is captured within a large cell line panel and often at a frequency similar to that observed in patient cohorts. However, the picture is far from complete; many CFEs occurring at low to moderate frequency (2%–5%) are represented by a single cell line or not at all, and coverage by cancer type is variable. As

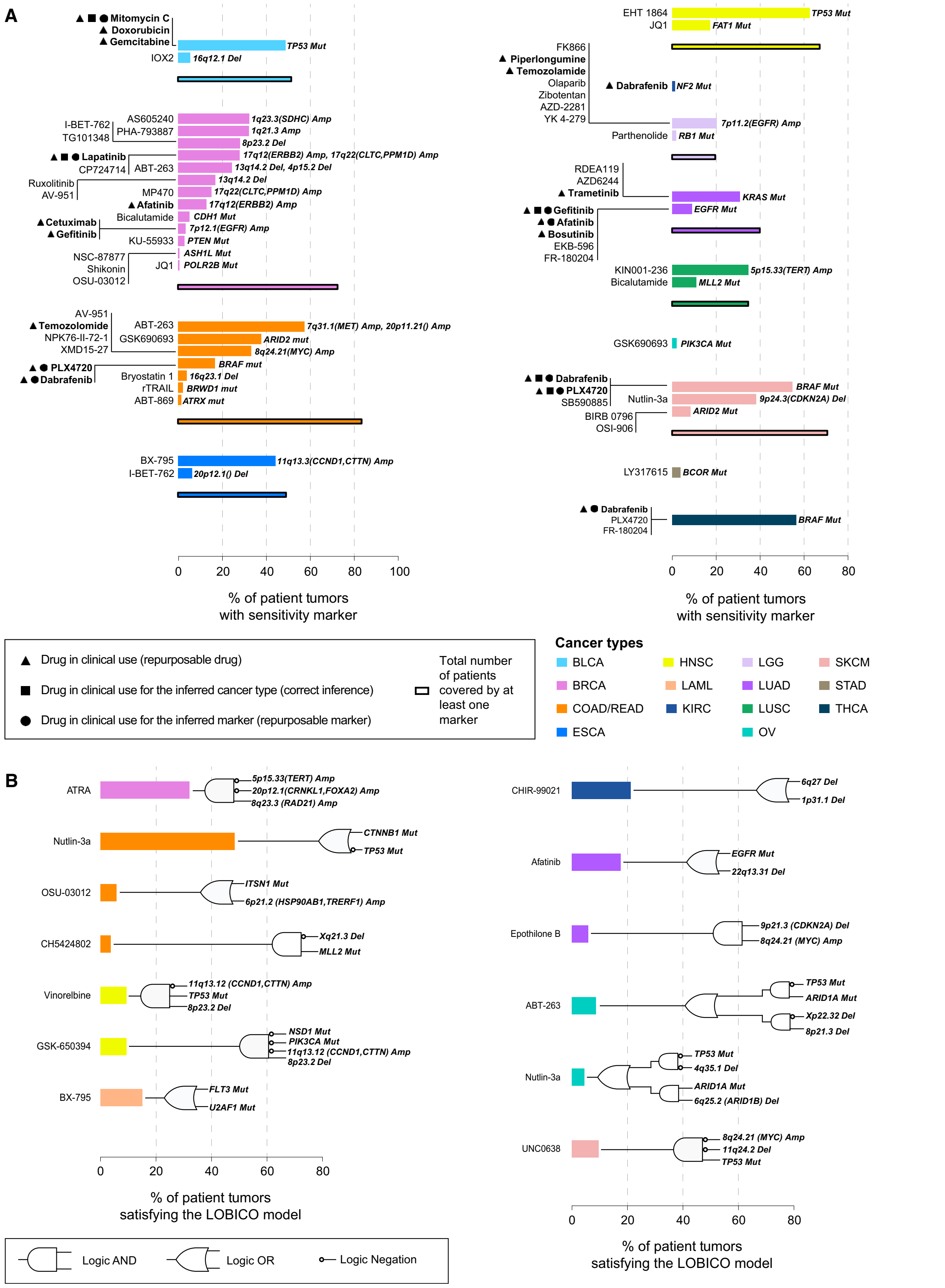


(legend on next page)

we enter an era of precision cancer medicine, where many drugs are active in small molecularly defined subgroups of patients (e.g., only 3%–7% of lung cancer patients harbor the drug sensitizing *EML4-ALK* gene fusion [Soda et al., 2007]), the scarcity of models for many cancer genotypes and tissues is a limitation. New cell culturing technologies enable derivation of patient cell lines with high efficiency and thus make derivation of a larger set of cell lines encompassing the molecular diversity of cancer a realistic possibility (Liu et al., 2012; Sato et al., 2011).

## Pharmacogenomic Models of Drug Sensitivity

Pharmacogenomic screens in cancer cell lines are an unbiased discovery approach for putative markers of drug sensitivity. We identified a wealth of molecular markers of drug sensitivity, including completely novel associations not easily explained with our current knowledge. With appropriate validation and follow-up studies, these putative biomarkers may aid patient stratification and help to explain the heterogeneity of clinical responses.

Going beyond single gene-drug interactions, "logic" combinations of CFEs consistently perform better than single events in sensitivity prediction. Clinical support for this comes from the observation that *BRAF* mutant melanoma patients treated with BRAF inhibitors show heterogeneity of response that may be explained by the presence of additional molecular alterations (Chapman et al., 2011). Our analyses suggest that clinical studies in cancer patients should be designed to enable combinations of genomic alterations to be detected, which has implications for both trial size and the statistical approaches employed.

We validated our pharmacogenomic models using independent datasets from the CCLE and CTRP. Consistent with previous reports, this demonstrated good consistency in the set of markers identifiable across these studies (Cancer Cell Line Encyclopedia Consortium, 2015) and lends additional support to the results presented here. However, our ability to validate some pharmacogenomic associations was restricted by the limited number of overlapping cell lines and compounds between these studies. Furthermore, the consistency between datasets is not perfect, and efforts toward standardization to reduce methodological and biological differences across the different studies are likely to improve future correlation between datasets.

## Glimpses of a Precision Medicine Landscape

For many of our pharmacological models, the defining CFE is present in clinical populations at a frequency that would make testing in a clinical trial setting feasible (Figure 7). For example, the alkylating agent Temozolamide (used to treat glioblastoma multiforme) shows activity in *MYC* amplified colorectal cancer lines (present in 33% of primary tumors) (Figure 7A). Overall, we found that a median of 50% of primary tumor samples harbor at least one CFE, or logic combination of CFEs, associated with increased drug response; ranging from 0.63% (OV) to 83.61% (COAD/READ) (Figure 7; Tables S7A–S7C; Supplemental Experimental Procedures). This suggests that there are likely to be a number of molecular subtypes within many cancers that, following appropriate validation, could be tested in the clinical trial setting using these stratifications for treatment selection.

Using machine learning, we determined that within each specific cancer type, genomic features (either driver mutations or copy number alterations) generated the most predictive models, with the addition of methylation data further improving our models. While informative in the pan-cancer setting, baseline gene expression data was less informative in the more clinically relevant tissue-specific setting. Prioritizing the design of diagnostics that deliver driver mutations, copy number alterations, and DNA methylation profiles might be the most cost effective means in the short-term to stratify patients for cancer treatment.

## Conclusions

The clinical development of molecularly targeted cancer therapies remains a formidable challenge. Our current analysis is restricted by the availability of patient genomic datasets, the cell lines and compounds screened, and methodological and biological variables, as well as the inherent limitation associated with the use of in vitro cancer cell lines. Nonetheless, our results represent a comprehensive attempt to describe the landscape of clinically relevant pharmacogenomics interactions in cellular models of cancer, complementing previous efforts (Barretina et al., 2012; Basu et al., 2013; Garnett et al., 2012; Seashore-Ludlow et al., 2015). The data resource and analyses described here should enable the matching of drug response with oncogenic alterations to provide insights into cancer biology and to accelerate the development of patient stratification strategies for clinical trial design.

# EXPERIMENTAL PROCEDURES

### Cancer Cell Line Characterization

Genomic data for a panel of 1,025 genetically unique human cell lines were assembled from the COSMIC database. 1,001 cell lines were included in this study (Table S1E). Variants and copy number alterations were identified as described in the Supplemental Experimental Procedures. Microsatellite instability data were assembled as detailed in the Supplemental Experimental Procedures. Gene fusions from a subset cell lines (~700) were identified by targeted PCR sequencing or split probe fluorescence in situ hybridization (FISH) analysis (Table S2C).

### Variant Identification in Tumors

Variant data from sequencing of 6,815 tumor normal sample pairs derived from 48 different sequencing studies were compiled (Rubio-Perez et al., 2015). To aid in the analysis, the tumor data were reannotated using a pipeline consistent with the COSMIC database (Vagrent: https://zenodo.org/record/16732#.VbeVY2RViko).

**Figure 7. A Precision Medicine Landscape**

(A) Percentages of primary tumor samples for each cancer type harboring a sensitivity marker to a given compound and the accumulate percentage of patients for all compounds.

(B) Percentages of primary tumors whose genomic features satisfy the logic model for sensitivity for a given drug. Corresponding logic circuits are shown to the right of the bars.

See also Table S7.

### Methylation Data

For primary tumors, raw data for 6,035 methylation samples, covering 18 tumor types, were downloaded from the TCGA data portal. For the cell lines, data were generated in-house as described in the Supplemental Experimental Procedures. In both cases, Infinium HumanMethylation450 BeadChip arrays were preprocessed using the R Bioconductor package Minfi. Only CpG site probes falling on the promoter region of the known genes were considered, i.e., TSS1500, TSS200, 5′ UTR, and 1st exon. Probes containing SNPs and non-specific probes, falling on sex chromosomes, and not associated with a gene were discarded. Methylation beta values of CpG islands were averaged across CpG sites.

### Identification of Cancer Functional Events

The selection of cancer-driver genes (together with the variant recurrence filter) of the recurrently copy-number-altered chromosomal regions and the informative CpG islands is detailed in the Supplemental Experimental Procedures.

### Gene Expression Data

Cell line pellets collected during exponential growth in RPMI or DMEM/F12 were lysed with TRIzol (Life Technologies) and stored at −70°C. Following chloroform extraction, total RNA was isolated using the RNeasy Mini Kit (QIAGEN). DNase digestion was followed by the RNAClean Kit (Agencourt Bioscience). RNA integrity was confirmed on a Bioanalyzer 2100 (Agilent Technologies) prior to labeling using 3′ IVT Express (Affymetrix). Microarray analysis was performed as described in the Supplemental Experimental Procedures.

### Cell Line versus Tumor Comparisons

All analyses evaluating the extent to which cell lines resemble primary tumors are detailed in the Supplemental Experimental Procedures.

### Cell Viability Assays

Experimental protocols used for compound screening are detailed in the Supplemental Experimental Procedures. Effects on cell viability were measured, and a curve-fitting algorithm was applied to this raw dataset to derive a multiparameter description of the drug response (half maximal inhibitory concentration ($IC_{50}$),and area under the curve [AUC]) through a multilevel mixed model (Vis et al., 2016) (Supplemental Experimental Procedures).

### Statistical Models of Drug Response

For each drug an ANOVA model was fitted to correlate drug response with the status of Cancer Functional Events (CFEs), as described in Garnett et al. (2012), implemented in GDSCtools (http//gdsctools.readthedocs.io) and detailed in the the Supplemental Experimental Procedures. The downsampling ANOVA simulation studies are detailed in the Supplemental Experimental Procedures. We applied the LOBICO (Knijnenburg et al., 2016) framework as detailed in the Supplemental Experimental Procedures. Machine learning models were computed as detailed in the Supplemental Experimental Procedures.

## ACCESSION NUMBERS

The accession numbers for the sequencing/copy number, transcriptional, and methylation data reported in this paper are, respectively, EGA: EGAS00001000978, GEO: GSE68379, and ArrayExpress: E-MTAB-3610.

## SUPPLEMENTAL INFORMATION

Supplemental Information includes Supplemental Experimental Procedures, six figures, and seven tables, and one data file and can be found with this article online at http://dx.doi.org/10.1016/j.cell.2016.06.017.

## AUTHOR CONTRIBUTIONS

Conceptualization, F.I., T.A.K., D.J.V., G.R.B., M.P.M., M.Sc., L.F.A.W., J.S.-R., U.M., and M.J.G.; Methodology, F.I., T.A.K., D.J.V., G.R.B., M.P.M., M.Sc., S.B., U.M., and M.J.G.; Software, F.I., T.A.K., D.J.V., M.P.M., M.Sc., T.C., H.L., and E.v.D.; Validation, F.I., T.A.K., D.J.V., M.P.M., N.A., S.B., H.L., P.G., and M.J.G.; Formal Analysis, F.I., T.A.K., D.J.V., M.P.M., and M.Sc.; Investigation, G.R.B., S.B., P.G., T.M., and L.R.; Resources, D.J.V., G.R.B., M.Sc., E.G., S.B., H.L., P.G., E.v.D., H.C., H.d.S., H.H., T.M., S.M., L.R., X.D., R.K.E., Q.L., X.M., J.W., T.Z., N.S.G., S.S., D.T., N.L.-B., P.R.-M., M.E., D.A.H., C.H.B., U.M., and M.J.G.; Data Curation, F.I., D.J.V., G.R.B., M.Sc., E.G., H.L., P.G., H.C., H.d.S., H.H., S.M., S.S., M.So., D.T., N.L.B., P.R.-M., L.F.A.W., J.S.-R., U.M., and M.J.G.; Writing – Original Draft, F.I., T.A.K., D.J.V., G.R.B., M.P.M., U.M., and M.J.G.; Writing – Review & Editing, F.I., T.A.K., D.J.V., G.R.B., M.P.M., M.Sc., N.A., L.F.A.W., J.S.-R., U.M., and M.J.G.; Visualization, F.I., T.A.K., M.P.M., M.Sc., and E.G.; Supervision, D.A.H., M.R.S., C.H.B., L.F.A.W., J.S.-R., U.M., and M.J.G.; Project Administration, F.I., U.M., and M.J.G.; Funding Acquisition, D.A.H., C.H.B., M.R.S., L.F.A.W., J.S.-R., U.M., and M.J.G.

## ACKNOWLEDGMENTS

This work was funded by the Wellcome Trust (086375 and 102696). F.I. was supported by the European Bioinformatics Institute and Wellcome Trust Sanger Institute post-doctoral (ESPOD) program. T.A.K. was supported by the National Cancer Institute (U24CA143835) and the Netherlands Organization for Scientific Research. D.T. was supported by the People Programme (Marie Curie Actions) of the 7th Framework Programme of the European Union (FP7/2007-2013; 600388) and the Agency of Competitiveness for Companies of the Government of Catalonia (ACCIÓ). N.L.-B. was supported by La Fundació la Marató de TV3. M.E. was funded by the European Research Council (268626), the Ministerio de Ciencia e Innovacion (SAF2011-22803), the Institute of Health Carlos III (ISCIII) under the Integrated Project of Excellence (PIE13/00022), the Spanish Cancer Research Network (RD12/0036/0039), the Health and Science Departments of the Catalan Government Generalitat de Catalunya 2014-SGR 633, and the Cellex Foundation. U.M. was supported by a Cancer Research UK Clinician Scientist Fellowship. We thank Aiqing He for expression data and Ilya Shmulevich for assistance with the LOBICO framework. We thank P. Campbell, M. Ranzani, J. Brammeld, M. Petljak, F. Behan, C. Alsinet Armengol, H. Francies, V. Grinkevich, and A. "Lilla" Mupo for useful comments. P.R.-M., H.C., and H.d.S. are employees and shareholders of Bristol-Myers Squibb. Research in the M.J.G. lab is supported in part with funding from AstraZeneca.

Received: August 7, 2015
Revised: December 23, 2015
Accepted: June 3, 2016
Published: July 7, 2016

## REFERENCES

Babur, Ö., Gönen, M., Aksoy, B.A., Schultz, N., Ciriello, G., Sander, C., and Demir, E. (2015). Systematic identification of cancer driving signaling pathways based on mutual exclusivity of genomic alterations. Genome Biol. *16*, 45.

Barretina, J., Caponigro, G., Stransky, N., Venkatesan, K., Margolin, A.A., Kim, S., Wilson, C.J., Lehár, J., Kryukov, G.V., Sonkin, D., et al. (2012). The Cancer Cell Line Encyclopedia enables predictive modelling of anticancer drug sensitivity. Nature *483*, 603–607.

Basu, A., Bodycombe, N.E., Cheah, J.H., Price, E.V., Liu, K., Schaefer, G.I., Ebright, R.Y., Stewart, M.L., Ito, D., Wang, S., et al. (2013). An interactive resource to identify cancer genetic and lineage dependencies targeted by small molecules. Cell *154*, 1151–1161.

Cancer Cell Line Encyclopedia Consortium; Genomics of Drug Sensitivity in Cancer Consortium (2015). Pharmacogenomic agreement between two cancer cell line data sets. Nature *528*, 84–87.

Chapman, P.B., Hauschild, A., Robert, C., Haanen, J.B., Ascierto, P., Larkin, J., Dummer, R., Garbe, C., Testori, A., Maio, M., et al.; BRIM-3 Study Group (2011). Improved survival with vemurafenib in melanoma with BRAF V600E mutation. N. Engl. J. Med. *364*, 2507–2516.

Ciriello, G., Miller, M.L., Aksoy, B.A., Senbabaoglu, Y., Schultz, N., and Sander, C. (2013). Emerging landscape of oncogenic signatures across human cancers. Nat. Genet. *45*, 1127–1133.

Cook, D., Brown, D., Alexander, R., March, R., Morgan, P., Satterthwaite, G., and Pangalos, M.N. (2014). Lessons learned from the fate of AstraZeneca's drug pipeline: a five-dimensional framework. Nat. Rev. Drug Discov. *13*, 419–431.

Costello, J.C., Heiser, L.M., Georgii, E., Gönen, M., Menden, M.P., Wang, N.J., Bansal, M., Ammad-ud-din, M., Hintsanen, P., Khan, S.A., et al.; NCI DREAM Community (2014). A community effort to assess and improve drug sensitivity prediction algorithms. Nat. Biotechnol. *32*, 1202–1212.

Garnett, M.J., Edelman, E.J., Heidorn, S.J., Greenman, C.D., Dastur, A., Lau, K.W., Greninger, P., Thompson, I.R., Luo, X., Soares, J., et al. (2012). Systematic identification of genomic markers of drug sensitivity in cancer cells. Nature *483*, 570–575.

Godin-Heymann, N., Ulkus, L., Brannigan, B.W., McDermott, U., Lamb, J., Maheswaran, S., Settleman, J., and Haber, D.A. (2008). The T790M "gatekeeper" mutation in EGFR mediates resistance to low concentrations of an irreversible EGFR inhibitor. Mol. Cancer Ther. *7*, 874–879.

Hanahan, D., and Weinberg, R.A. (2000). The hallmarks of cancer. Cell *100*, 57–70.

Kandoth, C., McLellan, M.D., Vandin, F., Ye, K., Niu, B., Lu, C., Xie, M., Zhang, Q., McMichael, J.F., Wyczalkowski, M.A., et al. (2013). Mutational landscape and significance across 12 major cancer types. Nature *502*, 333–339.

Knijnenburg, T., Klau, G., Iorio, F., Garnett, M., McDermott, U., Shmulevich, I., and Wessels, L. (2016). Logic models to predict continuous outputs based on binary inputs with an application to personalized cancer therapy. bioRxiv, doi: http://dx.doi.org/10.1101/036970.

Lawrence, M.S., Stojanov, P., Polak, P., Kryukov, G.V., Cibulskis, K., Sivachenko, A., Carter, S.L., Stewart, C., Mermel, C.H., Roberts, S.A., et al. (2013). Mutational heterogeneity in cancer and the search for new cancer-associated genes. Nature *499*, 214–218.

Lawrence, M.S., Stojanov, P., Mermel, C.H., Robinson, J.T., Garraway, L.A., Golub, T.R., Meyerson, M., Gabriel, S.B., Lander, E.S., and Getz, G. (2014). Discovery and saturation analysis of cancer genes across 21 tumour types. Nature *505*, 495–501.

Liu, X., Ory, V., Chapman, S., Yuan, H., Albanese, C., Kallakury, B., Timofeeva, O.A., Nealon, C., Dakic, A., Simic, V., et al. (2012). ROCK inhibitor and feeder cells induce the conditional reprogramming of epithelial cells. Am. J. Pathol. *180*, 599–607.

Mok, T.S., Wu, Y.-L., Thongprasert, S., Yang, C.-H., Chu, D.-T., Saijo, N., Sunpaweravong, P., Han, B., Margono, B., Ichinose, Y., et al. (2009). Gefitinib or carboplatin-paclitaxel in pulmonary adenocarcinoma. N. Engl. J. Med. *361*, 947–957.

Nelson, M.R., Tipney, H., Painter, J.L., Shen, J., Nicoletti, P., Shen, Y., Floratos, A., Sham, P.C., Li, M.J., Wang, J., et al. (2015). The support of human genetic evidence for approved drug indications. Nat. Genet. *47*, 856–860.

Parikh, J.R., Klinger, B., Xia, Y., Marto, J.A., and Blüthgen, N. (2010). Discovering causal signaling pathways through gene-expression patterns. Nucleic Acids Res. *38*, W109–W117.

Ross, D.T., Scherf, U., Eisen, M.B., Perou, C.M., Rees, C., Spellman, P., Iyer, V., Jeffrey, S.S., Van de Rijn, M., Waltham, M., et al. (2000). Systematic variation in gene expression patterns in human cancer cell lines. Nat. Genet. *24*, 227–235.

Rubio-Perez, C., Tamborero, D., Schroeder, M.P., Antolín, A.A., Deu-Pons, J., Perez-Llamas, C., Mestres, J., Gonzalez-Perez, A., and Lopez-Bigas, N. (2015). In silico prescription of anticancer drugs to cohorts of 28 tumor types reveals targeting opportunities. Cancer Cell *27*, 382–396.

Sato, T., Stange, D.E., Ferrante, M., Vries, R.G.J., Van Es, J.H., Van den Brink, S., Van Houdt, W.J., Pronk, A., Van Gorp, J., Siersema, P.D., and Clevers, H. (2011). Long-term expansion of epithelial organoids from human colon, adenoma, adenocarcinoma, and Barrett's epithelium. Gastroenterology *141*, 1762–1772.

Seashore-Ludlow, B., Rees, M.G., Cheah, J.H., Cokol, M., Price, E.V., Coletti, M.E., Jones, V., Bodycombe, N.E., Soule, C.K., Gould, J., et al. (2015). Harnessing connectivity in a large-scale small-molecule sensitivity dataset. Cancer Discov. *5*, 1210–1223.

Shaw, A.T., Kim, D.-W., Nakagawa, K., Seto, T., Crinó, L., Ahn, M.-J., De Pas, T., Besse, B., Solomon, B.J., Blackhall, F., et al. (2013). Crizotinib versus chemotherapy in advanced ALK-positive lung cancer. N. Engl. J. Med. *368*, 2385–2394.

Soda, M., Choi, Y.L., Enomoto, M., Takada, S., Yamashita, Y., Ishikawa, S., Fujiwara, S., Watanabe, H., Kurashina, K., Hatanaka, H., et al. (2007). Identification of the transforming EML4-ALK fusion gene in non-small-cell lung cancer. Nature *448*, 561–566.

Stratton, M.R., Campbell, P.J., and Futreal, P.A. (2009). The cancer genome. Nature *458*, 719–724.

Su, F., Viros, A., Milagre, C., Trunzer, K., Bollag, G., Spleiss, O., Reis-Filho, J.S., Kong, X., Koya, R.C., Flaherty, K.T., et al. (2012). RAS mutations in cutaneous squamous-cell carcinomas in patients treated with BRAF inhibitors. N. Engl. J. Med. *366*, 207–215.

Tamborero, D., Gonzalez-Perez, A., and Lopez-Bigas, N. (2013a). OncodriveCLUST: exploiting the positional clustering of somatic mutations to identify cancer genes. Bioinformatics *29*, 2238–2244.

Tamborero, D., Gonzalez-Perez, A., Perez-Llamas, C., Deu-Pons, J., Kandoth, C., Reimand, J., Lawrence, M.S., Getz, G., Bader, G.D., Ding, L., and Lopez-Bigas, N. (2013b). Comprehensive identification of mutational cancer driver genes across 12 tumor types. Sci. Rep. *3*, 2650.

van Dyk, E., Reinders, M.J.T., and Wessels, L.F.A. (2013). A scale-space method for detecting recurrent DNA copy number changes with analytical false discovery rate control. Nucleic Acids Res. *41*, e100.

Vis, D.J., Bombardelli, L., Lightfoot, H., Iorio, F., Garnett, M.J., and Wessels, L.F.A. (2016). Multilevel models improve precision and speed of $IC_{50}$ estimates. Pharmacogenomics *17*, 691–700.

Vogelstein, B., Papadopoulos, N., Velculescu, V.E., Zhou, S., Diaz, L.A., Jr., and Kinzler, K.W. (2013). Cancer genome landscapes. Science *339*, 1546–1558.

Wong, C.C., Martincorena, I., Rust, A.G., Rashid, M., Alifrangis, C., Alexandrov, L.B., Tiffen, J.C., Kober, C., Green, A.R., Massie, C.E., et al.; Chronic Myeloid Disorders Working Group of the International Cancer Genome Consortium (2014). Inactivating CUX1 mutations promote tumorigenesis. Nat. Genet. *46*, 33–38.

Zack, T.I., Schumacher, S.E., Carter, S.L., Cherniack, A.D., Saksena, G., Tabak, B., Lawrence, M.S., Zhsng, C.-Z., Wala, J., Mermel, C.H., et al. (2013). Pan-cancer patterns of somatic copy number alteration. Nat. Genet. *45*, 1134–1140.

# Supplemental Figures



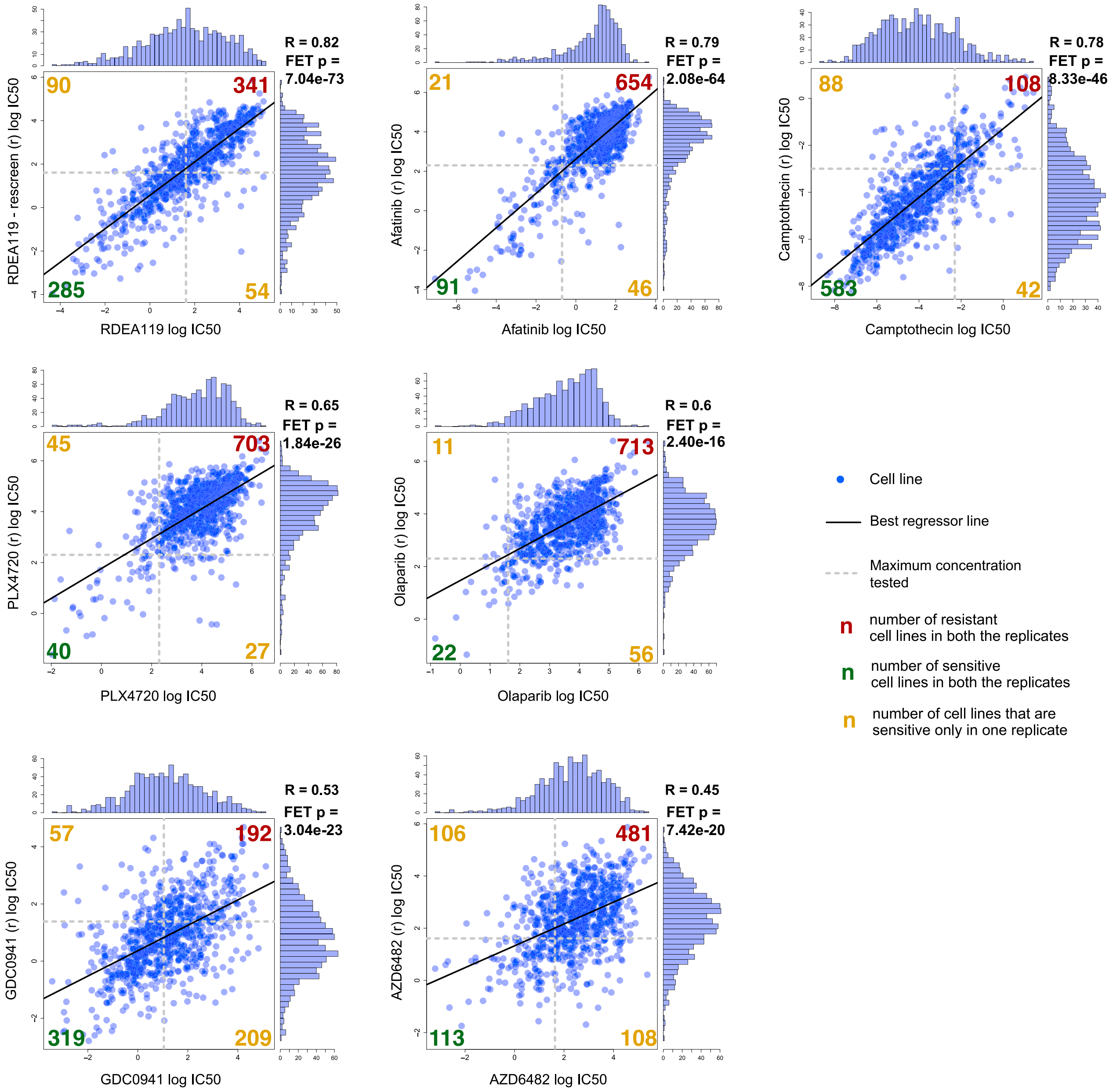


**Figure S1. Screened Compound Duplicates, Related to Figure 1**

Histograms, scatter plots and Pearson correlation scores between $IC_{50}$ profiles for 7 compounds screened in biological duplicates. In all cases replicate data were generated at least one year apart. Superimposed to each scatter plot is a contingency table (and a corresponding Fisher exact test p-value) showing consistency of sensitive ($IC_{50} \leq$ maximal tested concentration) and resistant ($IC_{50} >$ maximal tested concentration) cell lines across replicates.

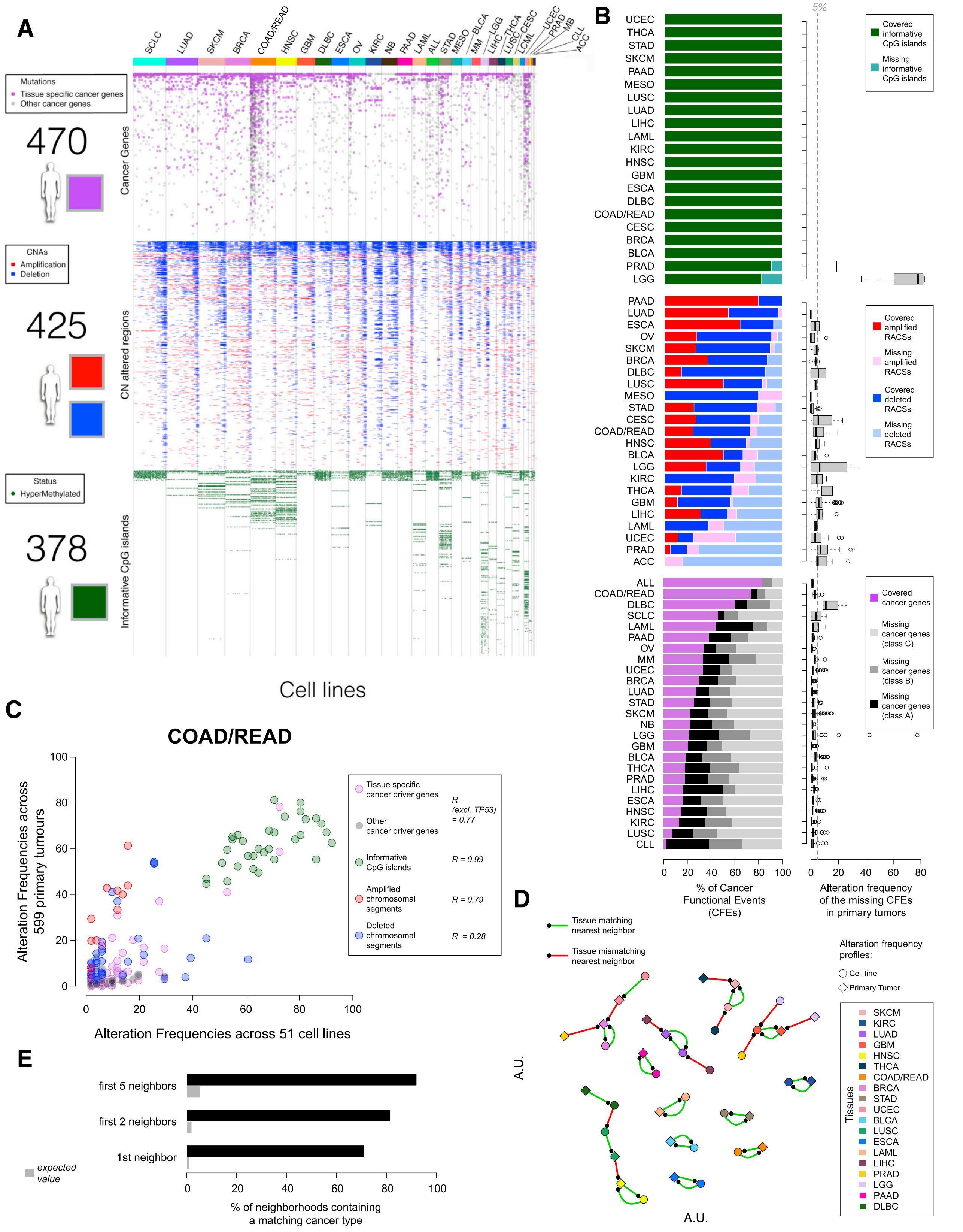


(legend on next page)

**Figure S2. Cancer Functional Events on Cancer Cell Lines, Related to Figure 2**

(A) Status of 1,273 Cancer Functional Events (CFEs) identified from primary tumor data in 1,001 cancer cell lines. Each column is a cell line, colors at the top indicate different cancer types, and each row is a CFE. The heatmap is horizontally divided in three parts with (i) high confidence cancer driver genes; (ii) focal recurrently aberrant copy number segments and (iii) informative CpG islands. A white space denotes absence of the functional events, whereas presence is indicated using the color schemes in the adjacent legends.

(B) Number of cancer-specific CFEs occurring in at least one cell line from the corresponding tissue, across the three molecular data types. Box plots on the right show the frequency of the missing CFEs in the primary tumors for each cancer type. Percentages of missing cancer genes for each cancer types are grouped based on their confidence (i.e., A = more than two signals of positive selection, B = two signals of positive selection, C = one signal of positive selection).

(C) Example of CFE frequency scatter plot for COAD/READ. Each circle is a CFE whose occurrence frequency across cell lines and primary tumors is given by its coordinates, respectively on the x- and y axis. Different CFE types are indicated by color and corresponding correlation scores are reported in the inset.

(D) Nearest neighbor analysis for similarities among cell lines and primary tumors based on frequency profiles accounting for all the CFEs. The proximity of two points is proportional to the correlation across the two corresponding CFE frequency profiles. A line connects a point to its closest neighbor (indicated by the small black dot).

(E) Performance of a k-nearest-neighbor classifier based on a comprehensive correlation distance between cell lines and primary tumors, accounting for all the CFEs.

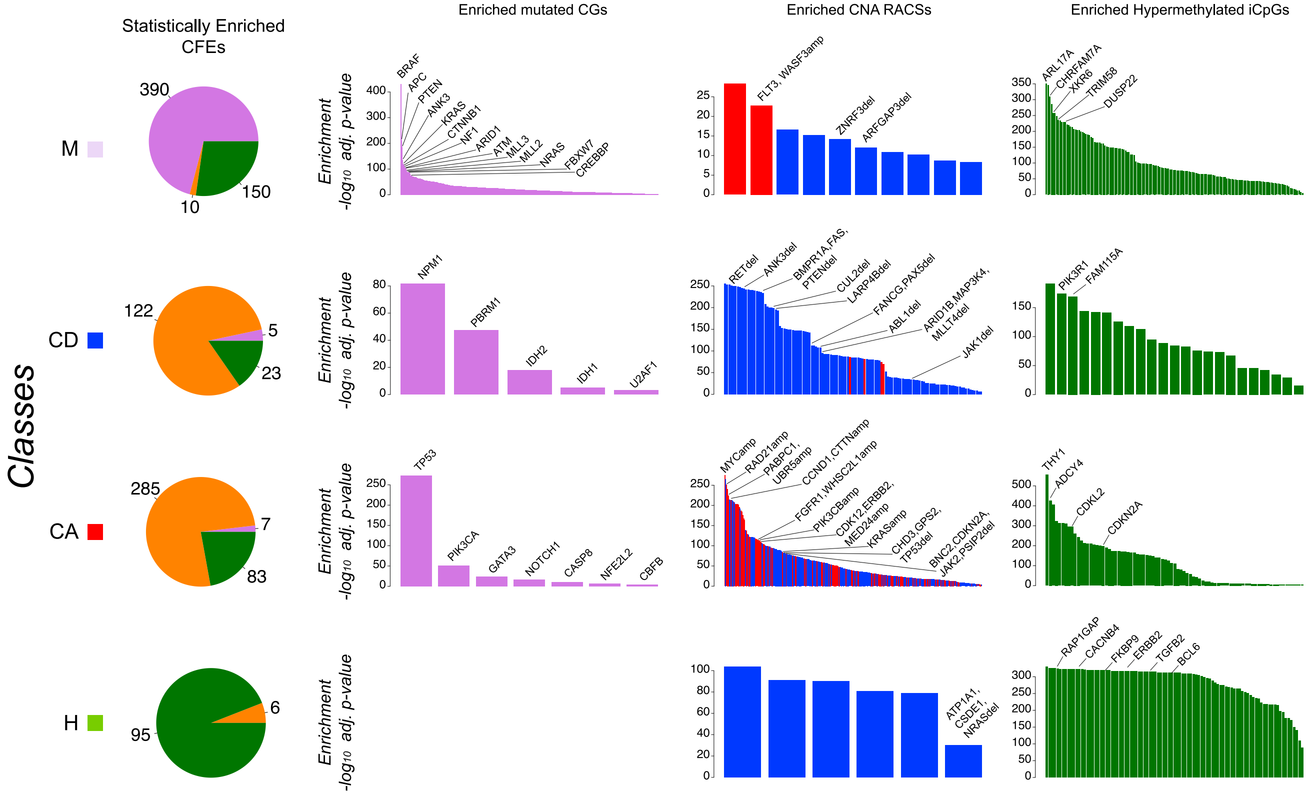


**Figure S3. Enrichment of Cancer Functional Events in Global Signatures, Related to Figure 3**

Enrichment analysis for global signatures of cancer functional events (CFEs) across different molecular data types identifies 4 classes of CFEs and cell-line/primary-tumor samples (on different rows). Pie charts on the left indicate the proportions of individually enriched CFE data types within each class (orange color indicates generic RACSs, both amplified and deleted). Bar diagrams on the right indicate, for each class and each CFE data type (on different columns), enrichment results for individual cancer functional events. Selected CFEs are highlighted.

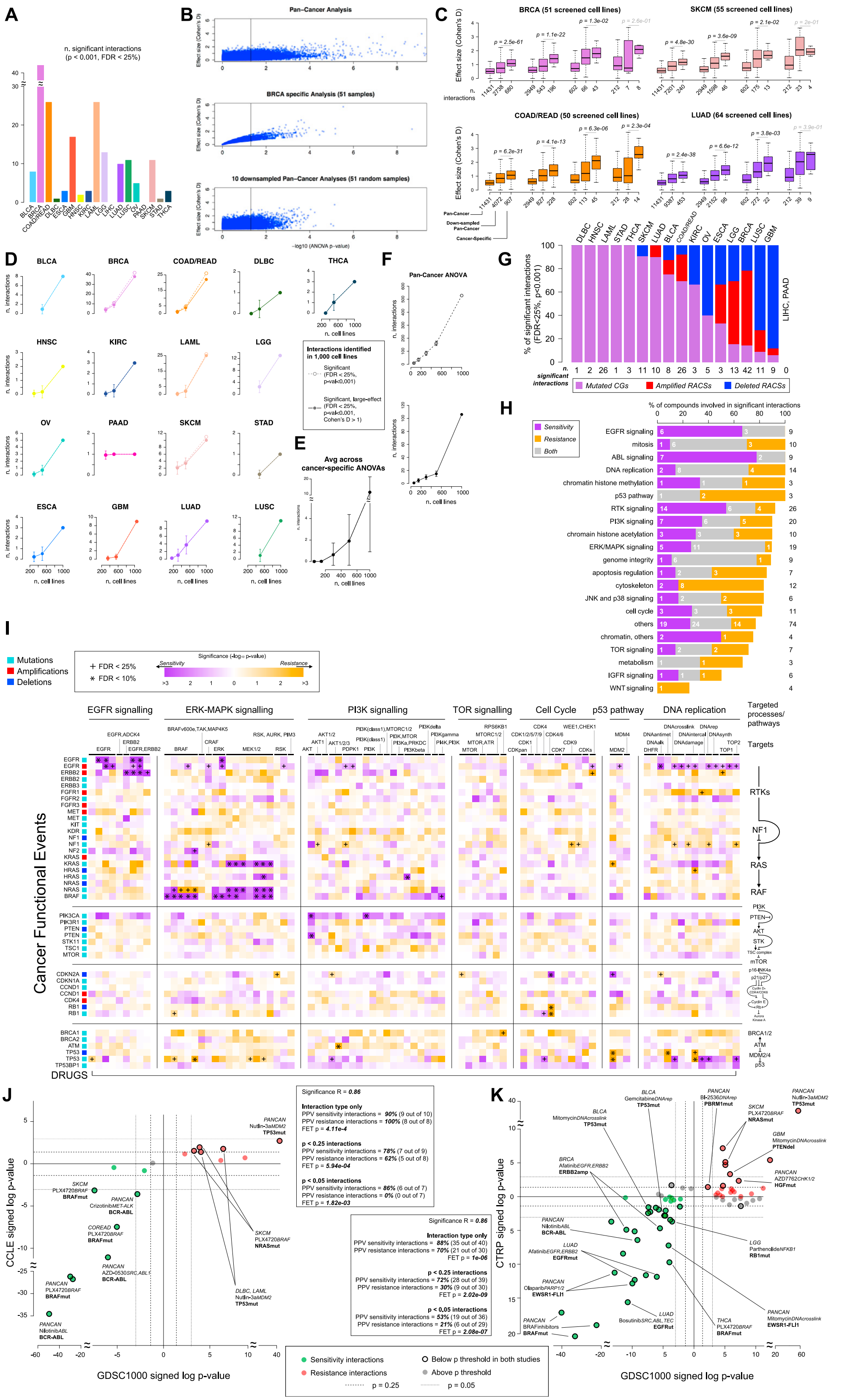

(legend on next page)

**Figure S4. ANOVA Result Summaries, Down-Sampled ANOVA Result Summaries, and ANOVA Validation Using CCLE and CTRP Datasets, Related to Figure 4**

(A) The number of statistically significant CFE-drug interactions for each cancer type.

(B) Example of ANOVA down-sampling analysis outcomes. Each point is a tested drug-CFE interaction, with position on the x-/y axis indicating significance and effect size, respectively. The vertical line correspond to the significance level $p = 0.05$. The effect size increment observed in the BRCA specific ANOVA is more evident and less variable than that observed in the down-sampled pan-cancer ANOVA.

(C) Effect-size variation for 4 different levels of statistical significance (indicated by the 4 groups of three box-plots) across pan-cancer, down-sampled, and cancer-specific ANOVAs. Each plot refers to a different cancer type (as indicated also by different colors). The effect size increment with respect to the pan-cancer analyses is consistently and significantly greater in the cancer-specific analyses than the down-sampled pan-cancer analyses. The total numbers of significant interactions (and the same value averaged across the sub-sampling simulations) according to the p-value threshold under consideration are reported.

(D) Number of significant (dashed lines) and significant large-effect (solid lines) pharmacogenomic interactions identified across 18 cancer-specific ANOVAs (using the whole panel of cell lines) that are retained in simulated down-sampled cancer-specific ANOVAs involving 500, 300, 160 and 60 cell lines. A missing dot means that, for the cancer type under consideration, a cancer-specific analysis is not possible due to reduced sample sizes.

(E) Average number of significant large-effect pharmacogenomic interactions identified across 18 cancer-specific ANOVAs (using the whole panel of cell lines) that are identifiable in simulated down-sampled cancer-specific ANOVAs.

(F) Number of significant (top plot) and significant large-effect (bottom plot) pan-cancer pharmacogenomic interactions that are identifiable in simulated down-sampled pan-cancer ANOVAs.

(G) Proportions of cancer functional event (CFE) types involved in significant pharmacogenomic interactions for each cancer type.

(H) Percentage of drugs involved in at least one significant CFE-drug interaction (pan-cancer or cancer-specific) across drugs classified into cancer associated pathways and processes.

(I) Pathway-centric overview of the identified pharmacogenomic interactions. Cells are color-coded according to corresponding $-\log_{10}$ p-values. Compounds are identified by the nominal therapeutic target.

(J) ANOVA results on overlapping GDSC-CCLE datasets. Each circle represents a drug-CFE association. The y axis is the signed $\log_{10}$ p-values of the identified interactions on the CCLE and the x axis that on the GDSC. Markers highlighted in red or green are significant in both studies. FET: Fisher exact test of consistency of marker behavior on all or only significant associations. A subset of associations is labeled with cancer-type, drug name, drug target (italics) and associated CFE (bold text).

(K) ANOVA results on overlapping GDSC-CTRP datasets.

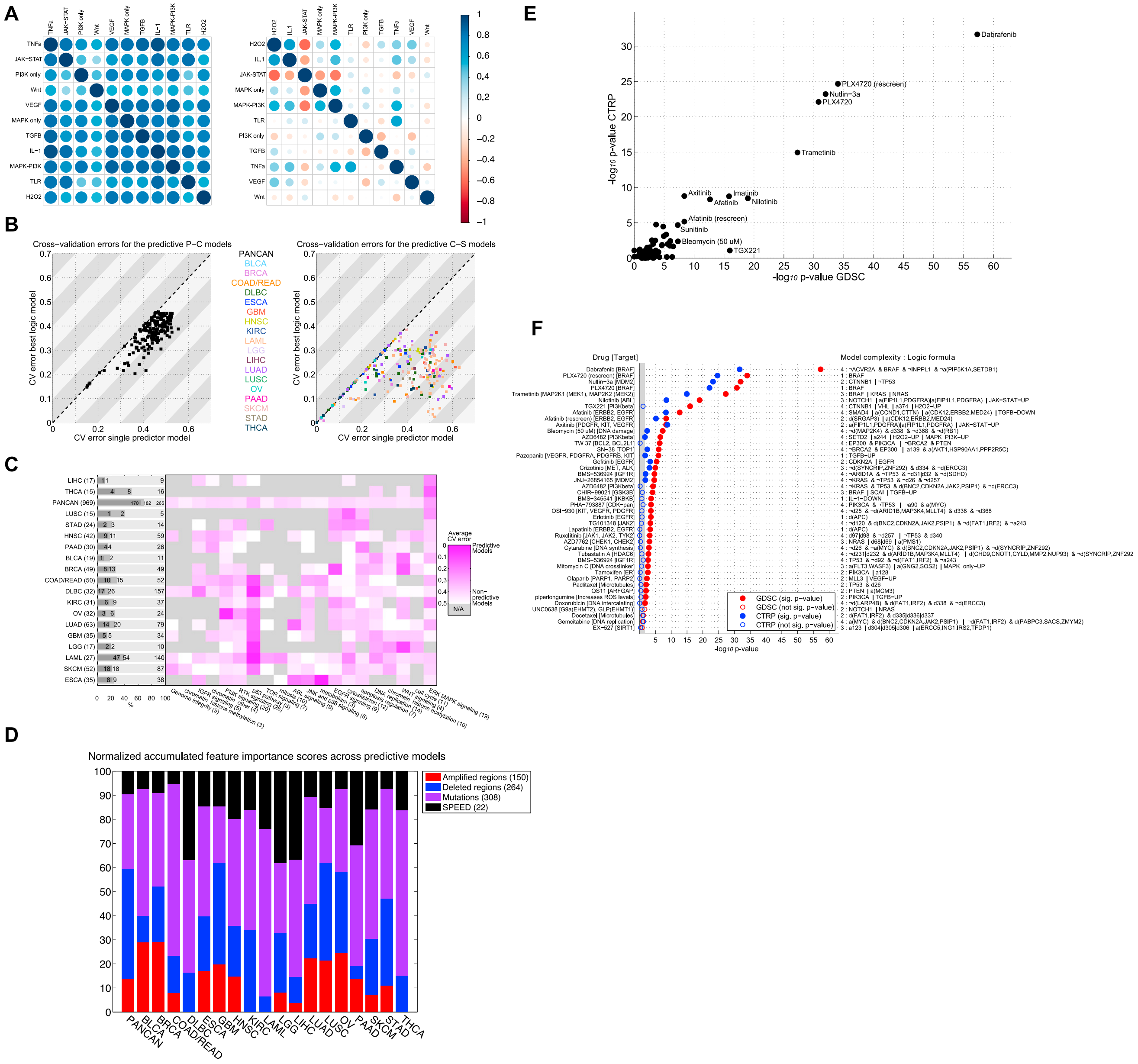

**Figure S5. LOBICO Performance and Validation of LOBICO Models on CTRP, Related to Figure 5**

(A) Pearson correlation of SPEED pathway activity scores across all cell lines using the original publication cutoffs (left) and our optimized cutoffs (right).

(B) Multi-predictor models outperform single predictor models. Scatter plot with the 5-fold cross-validation (CV) error for single predictor models (x axis) and the best (lowest CV error) multi-predictor model (y axis) averaged across 10 repeats for the cancer-specific datasets and 5 repeats for the pan-cancer dataset. Each point represents one of the 390 predictive logic models. The CV errors for the pan-cancer dataset (n = 182) are on the left; the CV errors from the 18 cancer-specific datasets (n = 208) are on the right.

(C) CV errors across cancer types and drug classes. Left: Number of drugs for which LOBICO was run, i.e., the drugs with 5 or more sensitive cell lines, number of drugs where a predictive model was inferred, and number of drugs, where the predictive model was a multi-predictor model, for the pan-cancer and each cancer-specific analysis. Center: CV error averaged across all drugs in a drug class (columns) for which LOBICO was run on the pan-cancer or cancer-specific dataset (rows). Grey indicates that no LOBICO models were run for the drugs in a drug class.

(D) Feature importance scores across data types: Normalized feature importance (FI) scores for each cancer type grouped into four categories (amplified RACSs; deleted RACSs; mutations in CGs; SPEED pathway activity). These scores were averaged across the drugs for which the LOBICO analysis was performed.

(E) t test p-values for LOBICO models on GDSC and CTRP. The scatter plot depicts the $-\log_{10}$ p-values for t tests that quantify the difference between cell lines predicted to be sensitive and resistant according to LOBICO. The x axis depicts p-values for the difference between these two groups based on the $IC_{50}$s within

(legend continued on next page)

GDSC. The y axis depicts p-values for the difference between these two groups based on the AUCs within CTRP. Drugs with a p-value lower than $10^{-7}$ are annotated.

(F) t test p-values on GDSC and CTRP for predictive LOBICO models. The scatter plot depicts the $-\log_{10}$ p-values for t tests that quantify the difference between cell lines predicted to be sensitive and resistant according to LOBICO. The 43 drugs are sorted based on the t test p-value derived from the GDSC $IC_{50}$s. P-values are considered significant at $p < 0.023$ (1/43).

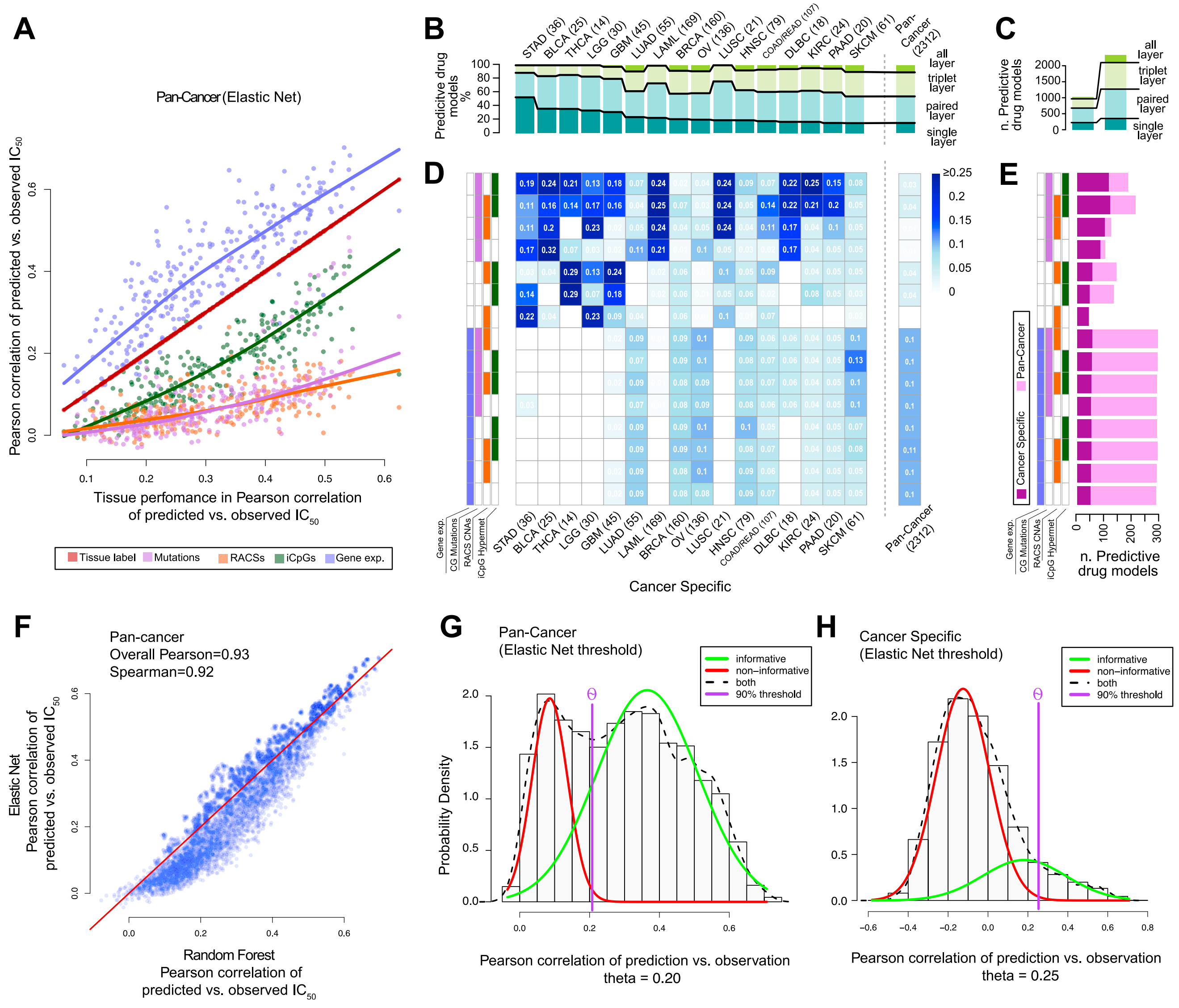


**Figure S6. Predictive Ability Assessment of Individual Molecular Feature Layers and Layer Combinations, Related to Figure 6**

(A) Predictive performance (Pearson correlation of predicted versus observed $IC_{50}$ values) of tissue label versus other feature layers in pan-cancer analysis with Elastic Net.

(B) Percentages of all the predictive models ($R_{pan\text{-}cancer} \geq 0.2$ and $R_{cancer\text{-}specific} \geq 0.25$) across different cancer types and molecular data type. Absolute counts of best performing models are indicated above the bars.

(C) Absolute counts of pan-cancer and cancer-specific models separated by number of feature layers.

(D) Heatmap split by cancer types and possible feature combination, showing the percentage of all predictive models.

(E) Count of all predictive models by data type combination separated in pan-cancer and cancer-specific analysis.

(F) Comparison of Random Forests versus Elastic Net performances in the pan-cancer analysis.

(G) Deriving pan-cancer threshold of predictive models by fitting a mixed Gaussian distribution across all build models, while assuming that one distribution is informative and the other one is not. A model is considered predictive if the ratio of informative to non-informative is at least 9, resulting in a minimal Pearson correlation of ~0.2 pan-cancer models achieving high performances due to tissue bias.

(H) Deriving cancer-specific threshold in same manner as for pan-cancer, resulting in minimal Pearson correlation of ~0.25. Negative correlations result from overfitting and too small sample sizes.

Cell, Volume *166*

## Supplemental Information

# A Landscape of Pharmacogenomic Interactions in Cancer

**Francesco Iorio, Theo A. Knijnenburg, Daniel J. Vis, Graham R. Bignell, Michael P. Menden, Michael Schubert, Nanne Aben, Emanuel Gonçalves, Syd Barthorpe, Howard Lightfoot, Thomas Cokelaer, Patricia Greninger, Ewald van Dyk, Han Chang, Heshani de Silva, Holger Heyn, Xianming Deng, Regina K. Egan, Qingsong Liu, Tatiana Mironenko, Xeni Mitropoulos, Laura Richardson, Jinhua Wang, Tinghu Zhang, Sebastian Moran, Sergi Sayols, Maryam Soleimani, David Tamborero, Nuria Lopez-Bigas, Petra Ross-Macdonald, Manel Esteller, Nathanael S. Gray, Daniel A. Haber, Michael R. Stratton, Cyril H. Benes, Lodewyk F.A. Wessels, Julio Saez-Rodriguez, Ultan McDermott, and Mathew J. Garnett**

**SUPPLEMENTAL EXPERIMENTAL PROCEDURES**

**Table of Contents**

1. Variant identification in cell lines
2. Microsatellite instability data
3. Selection of cancer driver genes
4. Variants recurrence filter
5. Identification of recurrently copy number altered chromosomal regions
6. Cell line methylation profiling
7. Identification of informative CpG island and methylation data discretisation
8. Transcriptional data pre-processing
9. Tumors/cell lines integrated analysis: Frequency profile comparisons
10. Tumors/cell lines integrated analysis: Knn classification performances and euclidean embedding
11. Tumors/cell lines integrated analysis: identification of classes of samples and selected functional events
12. Tumors/cell lines integrated analysis: enrichment analysis of event types across the identified classes
13. Tumors/cell lines integrated analysis: enrichment analysis of individual cancer functional event occurrences in a given class
14. Cell viability assay, dose response curve fitting model, comparison across replicates and compound cluster analysis
15. ANOVA model, effect-size, significance and p-value correction
16. ANOVA down-sampling studies
17. Pathway activity signatures and inference (SPEED analysis)
18. LOBICO to predicted drug response
19. LOBICO: cross-validation and feature importance scores
20. LOBICO: statistical test to identify predictive models
21. LOBICO: finding interesting pairwise AND and OR combinations
22. LOBICO: plotting a selected group of AND and OR combinations
23. Model selection, filtering and projection on primary tumor data
24. Validation of the ANOVA interactions and LOBICO models on independent datasets
25. Machine learning models

**1. Variant identification in cell lines**

After sequencing variants were identified by comparison to a reference genome. A matched lympoblastoid line was used as the reference genome for a small subset (n=39) of the cell lines where this was available. Differences from the reference genome were identified using the CaVEMan and Pindel algorithms identifying substitution and small insertions/deletions, respectively (https://github.com/cancerit). The resulting variants were then screened against approx. 8,000 normal samples to remove sequencing artefacts and germline variants (428 in-house normal exomes, 6500 normal exomes (NHLBI GO Exome Sequencing Project, June 20$^{th}$ 2012 release), 1000 genomes project (29$^{th}$ March 2012 release)) as well as variants in the DBSNP database (only those with associated minor allele frequency).

The remaining putatively somatic variants were classed as validated if present in other large scale cell-line sequencing datasets (the CCLE targeted sequencing (Barretina et al., 2012), NCI60 exome sequencing or previous capillary sequencing of 70 known cancer genes across 770 cell lines (Garnett et al., 2012)) and all such validated variants, together with other high quality variants (read depth $\geq$ 15 and a mutant allele burden $\geq$ 15% with no reads in the reference normal) were entered into the COSMIC cell line project database. Additional validation was carried out for putatively oncogenic 'low confidence' variants seen in

genes listed in the Cancer Gene Census (v67). Several transcripts are listed in COSMIC for some genes and this results in duplication of variants when exported, such duplicates were removed from the dataset.

All the BAM files have been deposited on the European Genome-phenome Archive (accession number: EGAS00001000978).

**2. Microsatellite instability data**

Analysis of microsatellite instability (MSI) was carried out according to the guidelines set down by "The International Workshop on Microsatellite Instability and RER Phenotypes in Cancer Detection and Familial Predisposition" workshop. Samples were screened using the markers BAT25, BAT26, D5S346, D2S123 and D17S250 and were characterised as MSI if two or more markers showed instability.

**3. Selection of cancer driver genes**

As described in (Rubio-Perez et al., 2015) we identified a set of high confidence cancer genes and we filtered individual variants based on the their occurrence frequency in COSMIC (v68) (http://cancer.sanger.ac.uk/cosmic/), as follows. We analysed sequence data from 6,815 matched tumor normal sample sets collated from 48 tumor-resequencing cohorts covering 28 major human cancer types. Briefly, three methods identifying complementary signals of positive selection were used: (a) OncodriveFM (Gonzalez-Perez and Lopez-Bigas, 2012), which identifies genes biased towards the accumulation of mutations with high functional impact; (b) OncodriveCLUST (Tamborero et al., 2013a), which identifies genes with an abnormal clustering of mutations across the protein sequence; and (c) MutSigCV (Lawrence et al., 2013), which identifies genes mutated at frequencies significantly above the background mutation rate. Genes stated as non-expressed in the corresponding cancer tissue were excluded from the observation set but used for the construction of the background models. The analysis was performed for each tumor cohort and in a pooled sample set to increase the statistical power to detect lowly-recurrent drivers acting consistently across several tumor types. The results of each method were combined by following the rationale explained by (Tamborero et al., 2013b). This analysis identified 461 cancer genes, which were classed as having evidence levels of A, B or C, as below and where genes with the former had the strongest evidence for selection and C the weakest. Level A = the gene exhibited more than one signal of positive selection (significant by more than one method) across one cohort of somatic mutations; Level B = the gene displayed only one single signal of positive selection in a certain cohort, and was a well-established cancer driver (i.e. included in the Cancer Gene Census); and Level C = the gene displayed only one single signal of positive selection, and was connected through a protein-protein or otherwise functional interactions (in the Reactome (Croft et al., 2014) or the PathwayCommons networks (Cerami et al., 2011)) to a gene with A or B evidence levels.

A recent analysis of nonsense mutations across 7,651 diverse tumors identified 55 putative recessive oncogenes (Wong et al., 2014), 46 of which were present in the set of 461 genes described above. The remaining 9 genes were added to the list of cancer genes (*AMOT*, *ASXL2*, *FTSJD1*, *LARP4B*, *MBD2*, *PHLPP1*, *RNF43*, *SACS* and *ZNRF3*). Therefore the final set consisted of 470 high confidence cancer genes.

We compared these results with the current version of the Cancer Gene Census, a manually curated catalogue of cancer genes, which is widely used as a benchmark (http://cancer.sanger.ac.uk/census). The current version (v72) contains 189 cancer genes driven by somatic point mutations, nonsense and frame shift mutations, of which 99 were identified in 1 or more of the 27 tumor types studied. The majority of the Cancer Gene Census genes not identified in this study are either found in tumor types not included in this analysis (e.g. basal cell carcinoma) and/or are mutated at very low frequency (e.g. FANCA in AML).

**4. Variants recurrence filter**

Variants from both the cell lines and tumors were screened against the 'systematic screen data' from COSMIC (v68) (derived from large-scale clinical datasets) to identify the recurrent variants most likely to contribute to carcinogenesis ('driver mutations'). We defined recurrence for missense variants, which are

generally activating and therefore 'pile-up' within a limited number of codons within the gene, differently to protein truncating mutations, which are inactivating events that occur across the footprint of the gene. For missense variants the number of non-synonymous variants in each codon of all genes within the systematic screen data in COSMIC (v68) was calculated, and any codon with ≥ 3 variants was classed as recurrently mutated. Inframe indels were treated in the same manner (although separately). With regards to protein truncating variants all genes that contained > 10 truncating variants (frameshifting indels, essential splice and nonsense mutations) within the systematic screen data were classed as recurrently inactivated.

**5. Identification of recurrently copy number altered chromosomal regions**

Segment copy number data was downloaded from the TCGA (Cancer Genome Atlas Research Network et al., 2013) (8,182 samples) and analysed with ADMIRE (van Dyk et al., 2013). The cohorts of COAD and READ were merged due to their high similarity in tissue type and response profile. The ADMIRE analysis results comprised copy number segments statistically different from expectation. Filter criteria were defined to focus the analysis on potential driver segments. The filter list required the segments to include at least one protein coding or antisense gene, but no more than 100 of them. It required the deletions to include an exon (a proxy for gene disruption) and amplifications to span a gene (as sub-genic amplifications are unlikely to be functional). The false discovery rate (FDR) controlled p-value was required to be smaller than 0.05, and the segment was required to be at recursion level two or higher unless it was a top-level segment. To ensure clinical relevance, the identified segment needed to be affected in at least 2.5% of the subjects. The latter was evaluated on two levels, using the overall background variance, and using the local background variance. The first was calculated on the $\log_2$ values not part of any identified segment, regardless of filtering. The second was calculated on the recursion level below the identified segment.

Within each tumor type the segments obtained after filtering (Table S2D) were further compacted by pruning all overlapping segments such that only the shortest were retained. This results in a fairly concise set of segments per tumor type. The pan-cancer set of segments was derived from the entire collection of filtered cancer specific segments, but only the largest overlapping segment was retained (Table S2E).

CEL files containing copy number profiles for all the cell lines in the panel (from SNP6.0 arrays) have been deposited on the European Genome-phenome Archive (accession number: EGAS00001000978)

**6. Cell line methylation profiling**

DNA samples were assessed for integrity, quantity and purity by electrophoresis in a 1.3% agarose gel, Picogreen quantification, and Nanodrop measurements. All samples were randomly distributed into 96 well plates. Bisulfite conversion of 500 ng of genomic DNA was performed using EZ DNA methylation kit (Zymo Research. Irvine) following manufacturer's instructions. 200 ng of bisulfite converted DNA were used for hybridization on the HumanMethylation450 BeadChip (Illumina, Inc. San Diego). Briefly, samples were whole genome amplified followed by an enzymatic end-point fragmentation, precipitation and resuspension. The resuspended samples were hybridized onto the beadchip for 16 hours at 48ºC and washed. A single nucleotide extension with labeled dideoxy-nucleotides was performed and repeated rounds of staining were applied with a combination of fluorescently labeled antibodies differentiating between biotin and DNP. Fluorescent signal from the microarray was measured with a HiScan scanner (Illumina, Inc. San Diego) using iScan Control Software (V 3.3.29).

Raw methylation profiles have been deposited on Gene Expression Omnibus (GEO) (accession number: GSE68379).

**7. Identification of informative CpG island and methylation data discretisation**

After pre-processing, the beta signal of each CpG island was analysed in the context of each cancer type *C*. Pre-processed beta-signals for primary tumors and cell-lines are available at http://www.cancerrxgene.org/gdsc1000/.

Due to the high tissue specificity of the epigenomic profiles, a systematic Hartigan's dip test for unimodality was executed on each of the beta signals across the tumor samples of *C* (for each *C* with methylation data available for at least 100 tumor samples), with the aim of identifying a set of CpG islands for which this signal did not distribute unimodally. Such CpG islands were deemed unlikely to be tissue-specific, hence consistently hyper methylated or hypo methylated across all the *C* tumor samples. Additionally, they were considered *informative*, because by using their bimodal beta signal distribution is it possible to stratify the tumor samples from *C* into two classes of lowly and highly methylated samples, respectively.

Once a set of informative CpG islands was identified, for each cancer type *C*, we found that for all of them the beta signal distribution was bimodal, hence we fitted a two Gaussians mixture model distribution to each of their beta signals, using the standard Expectation-Maximization (EM) algorithm. Finally, by examining the means of the two Gaussian distributions in the resulting fitted model, we labeled them as the generator of the low and high beta values, respectively. For each informative CpG islands we fixed a discretization threshold equal to the beta value for which the posterior probability of the high-beta distribution was at least 10-fold higher than that of the low-beta distribution. For each cancer type *C* for which methylation data for less than 100 tumor samples was available (DLBC, MESO, PAAD), or no methylation data was available from a matched tumor dataset (ALL, CLL, LCML, MB, MM, NB, OV, SCLC), we used as *C*-specific set the 19 CpG islands found informative in at least 2 other cancer types. In this case, a discretization threshold for a CpG island in this pooled set was computed as the median of all of its discretization threshold values obtained from the analyses of the cancer types with available tumor data (as explained above).

The cancer specific threshold value of each informative CpG islands was finally used to discretize its corresponding beta values across both the tumor and the cell line samples from the cancer type under consideration, thus coding all the methylation datasets in a binary fashion (where 1 indicates a relatively high-level of methylation and zero means a low level).

The R-code used to perform this analysis is available on request, all the intermediate results and plots are available at http://www.cancerrxgene.org/gdsc1000/, and the final list of informative CpG islands is reported in Table S2H.

**8. Transcriptional data pre-processing**

Microarray data generated as specified in the Experimental Procedures of the main text, was analyzed as described on the Human Genome U219 96-Array Plate using the Gene Titan MC instrument (Affymetrix). The robust multi-array analysis (RMA) algorithm (Irizarry et al., 2003) was used to establish intensity values for each of 18562 loci (BrainArray v.10, (Dai et al., 2005)).

Raw data was finally deposited in ArrayExpress (accession number: E-MTAB-3610). The RMA processed dataset is available at http://www.cancerrxgene.org/gdsc1000/.

**9. Tumors/cell lines integrated analysis: Frequency profile comparisons**

For each data-omic *D*, cancer type *C*, and sample type *T* (cell lines or tumors) a frequency profile *P(D,C,T)* was assembled, containing *n* entries: one for each of the cancer functional events (CFEs) involving genomic features from the *D* data-omic (i.e. high confidence cancer driver gene mutations, copy number alterations of recurrently amplified/deleted chromosomal regions, methylation status of informative CpG islands). The value of the *i-th* entry of this profile corresponded to the percentage of *T* samples from cancer type *C* in which the *i-th* CFE was present.

Once all these profiles were assembled for all the possible *(D,C,T)* triplets, for a given *D* we computed Pearson's correlation (*R*) scores between each pair of profiles *P(D,C,tumors)* and *P(D,C,cell lines)*, for all the cancer type *C* for which *D* data was available for tumors and cell lines both. Grouping the resulting *R*-scores based on the omic *D* and arranging them into pairwise comparison matrices $M_D$ (containing *m* rows and columns, with *m* = number of cancer types for which *D* data was available for cell lines and tumor

both), which the generic entry *i,j* contains $R(P(D,C_i,tumors), P(D,C_j,cell\ lines))$, yielded the results showed in the heatmaps of Figure 2B and Table S2L. To avoid correlation boosts due to consistently high frequency of occurrence across all the cancer types and sample types, the CFE accounting for *TP53*-mutations was excluded from these comparisons. Additionally to make the *R*-scores comparable across different cancer types, the same set of CFE (i.e. the pan-cancer set) was used to assembled each *P(D,C,T)* profile.

The tendency of the *R*-scores computed between profiles of cell lines and tumors from the same cancer type to be higher than those computed between profiles of cell lines and tumors from different cancer types (i.e. the statistical difference between the on/off diagonal values of the three $M_D$ matrices) was quantified with a Welch's t-test (yielding the box plots in Figure 2B).

**10. Tumors/cell lines integrated analysis: Knn classification performances and euclidean embedding**

To evaluate the performances of a K-nearest-neighbors classifier in assigning the correct cancer lineage to a given Cancer Functional Events (CFEs) frequency profile (computed as described in the previous section) by looking at its closest neighbor (i.e. the most correlated other profile), and to visualise all the profiles into a low dimensional space, the *R*-score computation was extended also to cell-line versus cell-line, and tumor versus tumor comparisons. In other words, for a given data omic *D* all the possible comparison $R(P(D,C_1,T_1), P(D,C_2,T_2))$, with $C_1$ and $C_2$ both belonging to the set of cancer types for which *D* data was available for cell lines and tumors both and $T_1$ and $T_2$ can be both cell lines or tumors. After this, all the resulting *R*-scores were scaled in [0,1] on a data-omic basis, then averaged across the different omics. The resulting final averaged set of *R*-scores (Table S2M) was finally used to compute the performances of a K-nearest neighbor classifier (Figure S2E) and to embed all the profiles into a bi-dimensional space where their pair-wise distance is inversely proportional to their pair-wise correlation (Figure S2D). This was obtained by making use of the t-SNE tool (Van der Maaten and Hinton, 2008), by giving as input to the dimensional scaling algorithm the final averaged set of *R*-scores, and performing 5,000 optimization-iterations with a perplexity parameter equal to 50% of the number of points to be embedded (i.e. the total number of cell lines and tumors frequency profiles).

**11. Tumors/cell lines integrated analysis: identification of classes of samples and selected functional events**

Two multi-omics datasets were assembled, respectively for cell lines and tumors, by pooling together all the Cancer Functional Events (CFEs) occurrence profiles across all the cell line and tumor samples and the three data omics layers (i.e. mutations, copy number alterations and hypermethylations). Subsequently these two datasets were merged together (Table S3B) and modeled as a bipartite network *G* (Table S3C, in simple interaction format (sif)), where the first class of nodes corresponded to CFEs, the second class of nodes corresponded to samples (both cell lines and tumors) and a link connected the node corresponding to the *i-th* CFE to the node corresponding to *j-th* sample, if the *i-th* CFE occurred in the *j-th* sample. A fast community detection algorithm, based on a greedy strategy (Newman, 2004) was then applied to this network, yielding 4 communities (i.e. groups of densely interconnected nodes), or classes, containing both CFE and sample nodes.

**12. Tumors/cell lines integrated analysis: enrichment analysis of event types across the identified classes**

Given a class $C = \{S,E\}$, where *S* is a set of sample-nodes and *E* is a set of cancer functional event (CFE) nodes, the statistical enrichment of a given CFE type *T* (i.e. mutated high confidence cancer genes, amplified and delete recurrently copy number altered chromosomal regions, and hypermethylated informative CpG islands) in *C* was quantified through a p-value assignment derived from an hypergeometric test with parameter *x, k, n, N*, where *N* was the total number of nodes corresponding to CFEs of any type in the bipartite network *G* (modeling the combined cell-lines/tumors dataset); n was the total number of nodes corresponding to CFEs of type *T* in in *G*; $k = |E|$ was the total number of CFE nodes in class *C*; *x* was the total number of nodes corresponding to CFE of type *T* in class *C*.

**13. Tumors/cell lines integrated analysis: enrichment analysis of individual cancer functional event occurrences in a given class**

Given a class $C = \{S,E\}$, where $S$ is a set of sample nodes and $E$ is a set of Cancer Functional Event (CFE) nodes, and an individual CFE $e$ belonging to $E$. The number of occurrences of $e$ across all the samples in the combined datasets was equal to the degree of the corresponding node in $G$, defined as above, (i.e. number of sample nodes connected to the e node). Then the statistical enrichment of these occurrences in class $C$ was computed through a p-value assignment derived from an hypergeometric test with parameter $x$, $k$, $n$, $N$, where $N$ was the total number of occurrences of all the CFEs across all the samples of the combined cell-line/tumor datasets (equal to the total number of links in the network $G$); n is the total number of occurrences of e in the combined cell-line/tumor datasets (equal to the degree of the e node in $G$); $k$ was the total number of links between nodes in $S$ and nodes in $E$ (i.e. the total number of links in class $C$); $x$ was the total number of links connecting the e node to nodes in $S$ (i.e. the total number of links connecting e to the sample nodes of $C$).

**14. Cell viability assay, dose response curve fitting model, comparison across replicates and compound cluster analysis**

The majority of the screened compounds were sourced from SelleckChem. We have adopted industry standards for the storage of compounds under inert conditions (low O2, dark, low humidity) to maximize their stability. Compound stability and precipitation have been monitored through visual inspection of compound solutions and using an Echo acoustic dispenser plate audit function. Furthermore, the same 10 cell lines have been screened every 2 - 3 months with compounds to confirm that compound activity was retained.

Cells were seeded in 384-well microplates at ~15% confluency in culture medium with 10% FBS and Penicillin/Streptomycin. The optimal cell number for each cell line was determined to ensure that each was in growth phase at the end of the assay (~85% confluency). For adherent cell lines, after overnight incubation cells were treated with either 9 concentrations of each compound (2-fold dilutions series), or 5 concentrations of each compound (4-fold dilution series), using liquid handling robotics (Beckman Coulter), and then returned to the incubator for assay at a 72-h time point. Cells were fixed in 4% formaldehyde for 30 minutes and then stained with 1µM of the fluorescent nucleic acid stain Syto60 (Invitrogen) for 1 hour. For suspension cell lines, cells were treated with compound immediately following plating, returned to the incubator for a 72-h time point, then stained with 55 µg/ml Resazurin (Sigma) prepared in Glutathione-free media for 4 hours. Quantitation of fluorescent signal intensity is performed using a fluorescent plate reader at excitation and emission wavelengths of 630/695 nM for Syto60, and 535/595 nM for Resazurin.

All screening plates were subjected to stringent quality control measures and to assess the quality of our screening a Z-factor score comparing negative and positive control wells was calculated across all screening plates.

In estimating the characteristic $IC_{50}$ dose-response value, all data is fitted in a single model (Vis et al., 2016). The assumed dose-response model is a two-parameter sigmoid that models the relative viability. The latter is obtained by scaling the observed intensities to the mean intensities of the control wells. Since the assumed response is strictly between 0 and 100% relative viability, the values are capped at 0 and 100.
Particularly, in this non-linear mixed effect model, it is assumed that the position and shape parameters vary.

$$f(x_{pos_{ij}}, x_{shape_i}, x) = \frac{1}{1 + e^{-\frac{x - x_{pos_{ij}}}{x_{shape_i}}}}$$

The parameters $x_{pos}$ and $x_{shape}$ are allowed to vary across the cell lines and for each cell line-drug pair a drug specific deviance is accommodated. On the cell line level, the two parameters are assumed to be correlated. The variation due to the drug is nested in the cell line level. The model is fitted using The R Project for Statistical Computing package nlme (Pinheiro et al., 2007). The x-positions represent the concentration dilution series, in which nine is the highest and one is the lowest screening concentration, such that a unit decrease translates to a two-fold dilution. Doubling the interval on x accommodates

alternative designs with five concentration points and four-fold dilutions. The interpretation of the dose-response curve as a function of the dilution series removes the scaling differences in the maximum test concentrations between the drugs, which ranges from the low nanoMolar (Bryostatin 1) to the milliMolar (DMOG) range. Note that the $IC_{50}$s are recorded as the natural logarithm of the half-maximal inhibitory μM concentrations

To evaluate reproducibility of results we screened 7 compounds in biological replicates observing a median Pearson correlation between patterns of $IC_{50}$ values across cell lines equal to 0.65 when considering all the 7 compounds, and equal to 0.78 when considering the compounds for which the majority of compared $IC_{50}$ values fell within the range of tested concentrations in at least one replicate (Figure S1). Furthermore, the sets of sensitive ($IC_{50} \leq$ maximal tested concentration) and resistant ($IC_{50} >$ maximal tested concentration) cell lines were highly overlapping across replicates, yielding statistically significant Fisher exact test p-values for all the 7 compounds.

Finally we performed a cluster analysis of the screened compounds based on similarity of their AUC profile across cell lines. Cell lines and compounds with less than 50% of AUC values available were not considered in this analysis and remaining missing AUCs were imputed with a k-nearest-neighbour (knn) approach. The first filtering reduced the original dataset (containing AUC values for 81% of all the possible compound/cell-line combinations) from 265 drugs and 990 cell lines to 223 drugs and 925 cell lines (with AUC values for 92% of the compound/cell-line combinations). On this dataset, the remaining 8% of missing AUCs were imputed through knn. The cluster analysis was performed through consensual non-negative matrix factorization (Brunet et al., 2004), with clustering cardinalities *k* ranging in [5,30], a maximal number of 500 iterations in each clustering and 20 clustering trials for each *k*. The optimal number of clusters was determined as a trade-off between the best cophenetic coefficient across the different values of *k* and the maximal *k*. Results are reported in Table S1G, together with a silhouette width score for each samples, quantifying how much it is well-placed in its cluster.

## 15. ANOVA model, effect-size, significance and p-value correction

A drug–response vector consisting of *n* $IC_{50}$ values from treatment of *n* cell lines was constructed for each drug. The model was linear (no interaction terms) with dependant variables represented by the described vector and factors including tissue type, and screening medium (for the pan-cancer analysis only), micro-satellite instability status (for the cancer types with positive samples for this feature) and the status of a CFE (one model for each CFE). These factors were selected based on a preliminary analysis assessing their impact on differential drug response in the pan-cancer context as well as for all the cancer types included in the study. This was performed through a type-II error ANOVA modelling drug response as a linear combination of the tissue of origin of the cell lines (only in the pan-cancer context), screening medium (i.e. DMEM or RPMI/F12) and growth properties (i.e. adherent, semi-adherent or suspension) (Table S1E). We observed that for growth properties these variables are essentially homogenous for cell lines within a specific cancer type, and so the contribution of these factors to the cancer-specific analysis is negligible. In contrast, media type has an impact on the response to several compounds when performing a pan-cancer analysis.

For the pan-cancer analysis, the union of all the cancer-specific CFEs (across omics layers and cancer types) was used. To reduce the number of tests, the set of informative CpG islands were excluded from this analysis. Additionally, only CFEs occurring in at least 3 cell lines were considered and CFEs with identical pattern of positive occurrence were merged together, thus resulting into a final set of 677 (individual or combined) features across 987 cell lines (screened against at least 1 drug). In order to include as many cell lines as possible in the pan-cancer analysis (even those not matching a TGCA type), values of the tissue factor were determined by looking at the GDSC.description_1 label in the cell lines annotation file (Table S1E). Whereas for the cancer-specific analysis, only cell lines with a matching TCGA label were used. The tissue factors corresponding to 'digestive_system' and 'urogenital_system' were further sub-classed by using the more specific GDSC.description_2 label.

For all the tested gene-drug associations, effect size estimations versus pooled standard deviation (quantified through the Cohen's d), effect sizes versus. individual standard deviations (quantified through

two different Glass deltas, for the CFE positive and the CFE negative population respectively), CFE p-values and all the other statistical scores where obtained from the fitted models. A CFE-drug pair was tested only if at least three cell lines were contained in the two sets resulting from the dichotomy induced by the CFE-status (i.e. at least 3 positive cell lines and at least 3 negative cell lines), for the pan-cancer and all the cancer-specific analyses as well.

The resulting p-values were corrected (all together those obtained in the pan-cancer analysis and on a cancer type basis those obtained in a given cancer-specific analysis), with the Tibshirani-Storey method (Storey and Tibshirani, 2003). A p-value threshold of $10^{-3}$ and a false discovery rate threshold equal to 25% were finally used to call significant associations across all the performed analyses. A pan-cancer and 18 cancer-specific (where at least 15 cell-line samples and sequencing and copy-number variation data was available for primary tumors) analyses were performed.

A Python package implementing the ANOVA analysis described in this section is available at http://gdsctools.readthedocs.io, together with detailed instructions on how to reproduce the results presented in our manuscript.

**16. ANOVA down-sampling studies**

This analysis aimed to assess whether the tendency of the cancer-specific interactions to associate with larger effect sizes (compared to the pan-cancer interactions) originates solely from the population-size reduction. Four cancer types (BRCA, COAD/READ, SKCM and LUAD) were selected because they are among those with the largest numbers of cancer specific associations and more than 20 available samples.

For a cancer type $C$ with $n$ available samples, 10 down-sampled pan-cancer ANOVAs were executed. To perform each of these analyses, a simulated cell line dataset was composed by randomly selecting $n$ samples from the pan-cancer input matrix of features and their columns corresponding to their corresponding positive CFEs. Then effect-sizes of the resulting ANOVA tests (for fixed levels of significance) where compared across pan-cancer, down-sampled and $C$-specific analyses. As expected, all the tested cancer types showed a strong consistent increase of effect size due to the down-sampling. Nevertheless, within the cancer specific analyses, this increment was significantly more evident and less variable.

A second down-sample ANOVA study was conducted to determine how many of the statistical significant and large-effect pharmacogenomic interactions (p-value < 0.001, FDR < 25%, Glass Δs > 1) reported in our study ($S$) would be still detectable if using reduced cell line sub-sets. To this aim, for $n$ = 500, 300, 150, and 60, and a number of iterations $k$ = 1,…,50, $n$ cell lines were randomly selected from the whole panel (reported in Table S1E), in a way that the spectrum of values of the tissue factor used in the pan-cancer ANOVA (assembled as described in the supplementary experimental procedures section 15), was preserved. On this set of cell lines a pan-cancer and 18 cancer-specific ANOVAs were performed. The number of significant and large-effect interactions $S_k$, were then determined in each of these $k$ iterations and the overlap between $S_k$ and $S$ assessed. The average cardinalities of these overlaps across all the iterations were then collected for all $n$ values, and reported in Figure S4D, S4E and S4F. These results show that only a limited number of the interactions identified based on an analysis of the whole panel of 991 screened cell lines would have been still detectable on n cell lines, with an average loss of 95% of high confidence cancer specific interactions and a loss of 70% of high confidence pan-cancer interactions already at the first n (= 500, Figures S4D, S4E and S4F).

**17. Pathway activity signatures and inference (SPEED analysis)**

To infer the activity of different signaling pathways, we used activation signature genes provided by the SPEED platform (Parikh et al., 2010). These are derived from multiple activation-response experiments using a total of 215 perturbations performed in 77 different experiments and covering 11 different signaling pathways [URL: http://speed.sys-bio.net/cgi-bin/database_statistics.py]. We chose this platform in favor of more recently derived breast cancer activation signatures (Gatza et al., 2010; 2014) to cover a large variation in experimental conditions and obtain signatures that are the consensus between different tissues. Those signatures represent genes that are differentially expressed when a pathway is perturbed. Comparing the expression level of those perturbation-response genes in the basal expression profile of different cell

lines gives us insight about the constitutive activity caused by mutations, copy number alterations, or other signaling aberrations. This activity may represent well-known mutational activations, but more importantly can also represent signaling activation for which there is no clear mutational marker available.

In order to derive consensus signature sets of genes for 11 different pathways the authors of (Parikh et al., 2010) defined the following quantities: z-scores – the number of standard deviations a certain gene is expressed in the perturbed condition compared to the basal condition; overall expression – the percentile of top expressed genes that a gene needs to belong to in order to be considered; overlap – in which percentage of experiments this needs to be the case; and uniqueness – if a given gene can be associated to more than one pathway.

In the original SPEED publication the authors suggested to use a constant cutoff of $z < 1\%$, expression > 50%, overlap > 20% and allowing non-unique genes. However, they only evaluated gene lists by their overlap with Gene Ontology categories, and not based on how well their enrichment scores are able to differentiate between microarrays where a given pathway perturbation is present and those where it is absent. This resulted in pathways that were highly correlated, as shown in Figure S5A. To counteract this, we performed a scan of the four adjustable platform parameters to optimize the order obtained by GSEA scores between control and stimulated experiments. The set of control arrays comprised all the un-stimulated arrays in the database, and the stimulated set all arrays in the database where a certain pathway was perturbed. This way, we allow cross-activation of pathways while minimizing the fit to random differences in gene expression by different initial conditions. We trained the parameters using 5x cross validation and selected the model with the best fit on the part of the data set not used for training. As a performance measure we used the area under the precision-recall curve that was obtained by the order of raw GSEA scores. Table S5B shows the improved separation between basal and pathway-perturbed arrays.
We calculated the raw basal GSEA scores for all cell lines using the gene lists obtained. As we do not have a background set, we transformed the resulting bimodal distribution for each pathway across all cell lines into a normal distribution using a kernel density estimator (kCDF function from the R package sROC) followed by the transformation $\log(x/(1 - x))$.

**18. LOBICO to predicted drug response**
As binary input features we used the set of cancer functional events (CFEs), excluding iCpGs and including gene expression derived binary pathway activity scores computed with the SPEED framework (Parikh et al., 2010) as detailed in SEP17.

Each of the 11 SPEED signatures resulted in two binary features: one representing upregulation by thresholding the continuous SPEED activity scores at a value of 3, and one representing downregulation (thresholding at a -3). These threshold values corresponded to three standard deviations above/below the mean of the normalized scores. The output feature was the response of the cancer cell lines to an anti-cancer drug as measured by the $IC_{50}$, discretized as detailed in below.

LOBICO was employed in a pan-cancer and 18 cancer-specific analyses and run for each drug individually, when the number of sensitive cell lines was 5 or higher. A cross-validation strategy was employed and feature importance scores derived as detailed in SEP19. Statistical tests to identify predictive models, selection of interesting pairwise AND and OR combinations and representative cases highlighted in Figure 5C, are detailed in the SEP20 to 22.
LOBICO finds the optimal logic function of the binary features that minimizes the error, defined as the sum of the weighted misclassified cell lines. The weight is proportional to the binarization threshold distance. Consequently, misclassification of cell lines close to the binarization threshold does not affect the optimization criterion, whereas there is a large penalty for misclassifying cell lines that are extremely sensitive or resistant to the drug. The total weight of each class is normalized in order to balance class importance. LOBICO was constrained to find solutions with a specificity of 0.8 or higher and applied with the same eight different complexities as in (Knijnenburg et al., 2016) ranging from simple single-predictor models to multi-input AND and OR models.

Note that the $IC_{50}$s were recorded as the natural logarithm of the half-maximal inhibitory μM concentrations. Although LOBICO uses the continuous $IC_{50}$ values, it is necessary to define a binarization threshold. This threshold is used to divide the cell lines into two classes: the sensitive cell lines and the resistant cell lines. We employed the procedure described in (Knijnenburg et al., 2016) to find the binarization threshold for each of the drugs. The binarization threshold for each of drugs was determined using all cell lines in the pan-cancer dataset.

There is one minor change in the 'upsampling' step of this procedure with respect to (Knijnenburg et al., 2016): In (Knijnenburg et al., 2016), the standard deviation of an $IC_{50}$ was derived from the confidence interval of the $IC_{50}$. Here, the standard deviation of an $IC_{50}$ was set 0.2, which was the typical across all $IC_{50}$s. This adjustment was made because we employed a different curve fitting algorithm compared to our previous work (Garnett et al., 2012). The new curve fitting algorithm does not provide a confidence interval per $IC_{50}$. Additionally, parameter $t$ was set to 0.03 instead of 0.05 in (Knijnenburg et al., 2016).

## 19. LOBICO: cross-validation and feature importance scores

For each LOBICO analysis, a stratified 5-fold cross-validation (CV) strategy was employed. The 5-fold CV was repeated 10 times for the cancers-specific datasets and 5 times for the pan-cancer dataset with different random seeds for assigning samples to test and training folds. We derived feature importance (FI) scores for each of the binary features for each inferred logic model of a certain model complexity. Additionally, we computed aggregated FI scores, which are based on all models complexities that have a CV error equal or smaller than the CV error for the single-predictor model.

## 20. LOBICO: statistical test to identify predictive models

We implemented a straightforward statistical test to decide whether an inferred logic model performed significantly better than random. First, we selected the optimal model complexity of the employed logic model, i.e. the model complexity with the lowest average CV error. Then, we took the inferred class labels (sensitive (1) and resistant (0)) of the inferred logic model (with the optimal complexity) when applied to the test folds in the CV. We did this for each of the repeats, and counted the average number of predicted sensitive (1) cell lines across the repeats, say $x$. We created 1,000,000 binary vectors with the original length (number of cell lines) with ones in $x$ random positions and recorded the error (i.e. the sum of the weighted misclassified cell lines) associated with each permutation. The permutated errors, the mean of which is 0.5, were compared to the original CV error. The permutation test P-value was computed using EPEPT (Enhanced P-value Estimation for Permutation Tests) (Knijnenburg et al., 2011; 2009). This was done for all 1112 logic models. We derived Q-values for each of the 1112 P-values. A logic model was called 'predictive' when both its p-value and q-value (FDR) were smaller than 0.05. The performance of the inferred logic models was measured using the normalized CV error. This error is between 0 and 1, and is 0.5 in the case of randomly predicting the sensitivity of cell lines.

## 21. LOBICO: finding interesting pairwise AND and OR combinations

We identified relevant AND and OR combinations of CFEs that explain drug sensitivity. We considered the AND and OR pairs that met the following constraints across all repeats (5 repeats for the pan-cancer dataset and 10 repeats for the cancer-specific datasets):

- The pairwise combination should be present in the logic formula of the optimal or sub-optimal solution of a predictive model ($p$-value $< 0.05$, $q$-value $<0.05$), either in the logic formulas associated with the optimal model complexity (according to CV) or in logic formulas with lower model complexity.
- The pairwise combination should be present in the predictive model of a drug in at least 4 of the 6 folds (the 5 CV training folds plus the model on the complete dataset).
- The pairwise combination should be found across at least 2 drugs in the same drug class.
- The feature importance (FI) score of each member of the pair should be higher than 0.03.
- The sum of the feature importance scores of both members of the pair should be higher than 0.10.

**22. LOBICO: plotting a selected group of AND and OR combinations**

The arrows that are highlighted in color and annotated with text in Figure 5C meet the following constraints (applied in this order):

- The drug associated with the arrow is clinically approved as notated in Table SF1.
- The difference in the F-measure (harmonic mean of precision and recall) when going from the single predictor model to the combination is at least 0.04.
- The improvement in precision or recall when going from the single predictor model to the combination is at least 0.10.
- At most one feature in a combination can be a binarized pathway activity (SPEED signature).
- Per quadrant, a combination cannot occur more than twice. (The same combination can occur for different drugs and different cancer types.) In case of more than two instances of the same combination, we selected the two with the highest differential F-measure.
- Per quadrant, a drug cannot occur more than once per cancer type. In case of more than one instance of the same drug for a particular cancer type, we selected the one with the highest differential F-measure.
- Per quadrant, a drug cannot occur more than three times. In case of more than three instances of the same drug, we selected the three with the highest differential F-measure.
- Per quadrant, no more than two colored arrows per cancer type are allowed. In case of more than two instances of the same cancer type, we selected the two with the highest differential F-measure.

The grey arrows in the background represent all combinations mentioned in the main text and are available in Table S5F. However, for clarity at most 10 grey arrows were plotted per cancer per quadrant based on the differential F-measure.

**23. Model selection, filtering and projection on primary tumor data**

The models shown in Figure 7B have been selected as follows: from the whole list of predictive models in Table S5E, we selected those (i) obtained in cancer-specific analyses, (ii) guaranteeing a cross-validation error lower than 0.25, (iii) not including SPEED pathway activity scores, (iv) not including negations only as terms, (v) not including (for the cancer type under consideration) not-cancer-specific cancer genes, (vi) referring to cancer types for which primary tumor samples with both mutations and copy number alteration data were available.

From the selected models we removed terms containing features whose relative importance is lower than 0.05, and dropped out the resulting filtered models with only one input feature in input.
The final list of selected models is contained in Table S7C. From this list, those models for which the difference between the percentage of primary-tumor and cell-line samples satisfying them was greater than 25 were not visualized in Figure 7B.

**24. Validation of the ANOVA interactions and LOBICO models on independent datasets**

Drug response data in the Cancer Cell Line Encyclopaedia (CCLE) (Barretina et al., 2012) dataset (latest version) was downloaded from the CCLE web-portal:
http://www.broadinstitute.org/ccle/data/browseData?conversationPropagation=begin
(file: CCLE_NP24.2009_Drug_data_2015.02.24.csv: Pharmacologic profiles for 24 anticancer drugs across 504 CCLE lines, 24-Feb-2015).

Drug response data in the Cancer Therapeutic Response Portal version 2 (CTRP) dataset (Seashore-Ludlow et al., 2015) was downloaded from the supplementary information files of the corresponding main publication, available online on the Cancer Discovery journal web-site at:
http://cancerdiscovery.aacrjournals.org/content/early/2015/10/14/2159-8290.CD-15-0235/suppl/DC1
(Supplemental Tables S1 – S7, file: 145780_2_supp_3058746_nrhtdz.xlsx).

From these files, identifiers of screened cell-lines and compounds in the two studies were extracted and mapped to the cell-lines and compound identifiers of our study (refered to as GDSC). This resulted in 389 overlapping cell-lines, the $CL_{GDSC\text{-}CCLE}$ set (Table S4E), and 15 overlapping compounds for the CCLE, the $D_{GDSC\text{-}CCLE}$ set (Table S4F), and 466 overlapping cell-lines, the $CL_{GDSC\text{-}CTRP}$ set (Table S4I), and 76 overlapping compounds for the CTRP, the $D_{GDSC\text{-}CTRP}$ set (Table S4J). All the available drug response indicators included in the CCLE and CTRP where extracted for all the cell-lines and compounds in $CL_{GDSC\text{-}CCLE}$ and $D_{GDSC\text{-}CCLE}$ from the CCLE file mentioned above, and for all the cell-lines and compound in $CL_{GDSC\text{-}CTRP}$ and $D_{GDSC\text{-}CTRP}$ from the CTRP file mentioned above. For CCLE, the drug response indicators are the half-maximal inhibitory concentrations ($IC_{50}$s) and the Compound Activity Areas, defined as 1 – the area under the drug/cell-line dose response curve (AUC). For the CTRP the drug response indicators are the AUCs; $IC_{50}$ values were not available for the CTRP study. These drug response indicators are contained in Table S4G and Table S4K, side-by-side with corresponding $IC_{50}$s and AUCs from the GDSC, together with information on the range of tested concentrations across the considered screenings.

To validate the pan-cancer and cancer-specific interactions identified through ANOVA we took the following strategy:

For each of the studies CCLE and CTRP, here denoted by *S*:

*i*) Pan-cancer and cancer-specific high-confidence pharmacogenomic interactions $I_{GDSC\text{-}S}$ were extracted from those originally identified in the GDSC (Table S4C). This subset $I_{GDSC\text{-}S}$ included only interactions involving compounds in the $D_{GDSC\text{-}S}$ set, with an ANOVA p-value < $10^{-3}$, both Glass Δs (measures of the effect size of the interaction) > 1, and FDR < 25%. This resulted in a subset of 32 interactions for $I_{GDSC\text{-}CCLE}$ and 106 interactions for $I_{GDSC\text{-}CTRP}$.

*ii*) Each interaction $i$ = *(CFE, Compound)* in $I_{GDSC\text{-}S}$ was retested two times on the subset of cell lines in $CL_{GDSC\text{-}S}$ using $IC_{50}$ values from the GDSC, and the drug response indicator from the study *S,* i.e. $IC_{50}$ values if *S = CCLE,* and AUC values if *S = CTRP*. These newly performed ANOVA tests were executed using the same settings of the GDSC ANOVA described in SEP 15. Specifically, we used tissue of origin, screening medium, micro-satellite instability (MSI) in addition to the cancer functional event (CFE) under consideration as co-factors for pan-cancer interactions, and only MSI in addition to the CFE under consideration for cancer-specific interactions. In the case that in the GDSC or in *S*, there were not sufficient drug response observations were available for the cell lines in $CL_{GDSC\text{-}S}$ (threshold values for the ANOVA factors described in SEP 15) then *i* was deemed not-testable on the study under consideration, i.e. GDSC or *S*. For *S* = CCLE, 22 out of 32 interactions in $I_{GDSC\text{-}S}$ were re-testable on both GDSC and CCLE. For *S* = CTRP, 70 out of 106 interactions in $I_{GDSC\text{-}S}$ were re-testable on both GDSC and CTRP. In each test, the status of the CFE under consideration was determined using data from the GDSC.

*iii*) Each of the interactions in $I_{GDSC\text{-}S}$ that was re-testable on both the GDSC and *S* when restricting the analysis on $CL_{GDSC\text{-}S}$, was considered validated on *S* if both the re-performed ANOVA tests yielded a p-value smaller than a given threshold *P* and the test had the same 'interaction sign', i.e. increased drug sensitivity or drug resistance. Different threshold values for *P* values were used: 1 (i.e. concordance of the interaction sign only), 0.25 (lax threshold), and 0.05 (stringent threshold).

*iv*) To assess the overall reproducibility of significant ANOVA associations in GDSC and *S*, we built and analyzed contingency tables that compare counts of significant interactions in GDSC and *S*. Specifically, for each threshold value of P, we assembled a 3x3 table as follows. The rows of a 3x3 contingency table accounted for the total number of testable interactions that were significantly sensitive (1st row), non-significant (2nd row), and significantly resistant (3rd row), on $CL_{GDSC\text{-}S}$ when using $IC_{50}$ values from the GDSC, whereas the columns accounted for the total number of testable interactions that were significantly sensitive (1st column), non-significant (2nd column), and significantly resistant (3rd column), on $CL_{GDSC\text{-}S}$ when using the drug response indicator values from *S*. Additionally, we derived 2x2 contingency tables from the 3x3 matrices by discarding the central row and column, i.e. accounting only for significant interactions in both the

studies. Fisher exact tests were performed on these contingency matrices for all the considered threshold *P* values.

*v*) Finally, a Pearson correlation test was performed between the two patterns of ANOVA $-log_{10}$ p-values resulting from the two re-performed tests described in *ii,* across all the testable interactions in $I_{GDSC-S}$.

Results from this analysis have been assembled in Table S4H and Figure S4J for *S* = CCLE, and Table S4L and Figure S4K for *S* = CTPR.

To validate the LOBICO models we followed a similar strategy. Here, we focused solely on the CTRP dataset, since the CCLE has only 15 drugs in common with the GDSC. Specifically, we ran LOBICO for the 466 common cell lines between the GDSC and the CTRP (Table S4I) for each of the 76 common compounds (Table S4J) using the same settings as for the original analysis (described in paragraphs 18 to 22). Of note, the GDSC contains rescreens or duplicate screens for 10 of the 76 compounds resulting in a total of 86 LOBICO analyses.

For each of the 86 analyses, we selected the best model according to cross-validation (CV) and applied that model to the cell lines dividing them into a group that is predicted to be sensitive and a group that is predicted to be resistant. Then, we performed a t-test comparing these two groups both for the GDSC $IC_{50}$s as well as for the CTRP AUCs. The t-test was only performed when both groups had at least 5 cell lines.

**25. Machine learning models**

Predictive models of drug activity (i.e. $IC_{50}$) for each compound, using 4 different molecular data types and their combinations, were built across 18 cancer types and across a pan-cancer setting with Elastic Net (EN) and Random Forests (RF), for a total of 137,726 models. All models were 1,000 times bootstrapped, with 80% of the data used for training, 10% for cross-training (determining model parameters) and the remaining 10% exclusively for testing. Based on the cross-training the EN mixing parameter (between 0 and 1, in intervals of 0.1) and number of input features were fitted for the EN regression, using the R-package 'glmnet' version 2.0-2. The RF implementation was based on R-package 'randomForest' version 4.6-10 and during cross training the number of trees was determined (between 10 and 1000, in intervals of 10). Using the test set, we estimated predictive power by calculating Pearson correlation between the predicted and observed drug activity. Models are assumed predictive if their observed Pearson correlation has a likelihood of over 90% to be derived from the informative versus the non-informative distribution across all models, which for pan-cancer and cancer-specific setting are ~0.2 (Figure S6G) and ~0.26 (Figure S6H), respectively.

## SUPPLEMENTAL REFERENCES


Barretina, J., Caponigro, G., Stransky, N., Venkatesan, K., Margolin, A.A., Kim, S., Wilson, C.J., Lehár, J., Kryukov, G.V., Sonkin, D., et al. (2012). The Cancer Cell Line Encyclopedia enables predictive modelling of anticancer drug sensitivity. Nature 483, 603–607.

Brunet, J.-P., Tamayo, P., Golub, T.R., and Mesirov, J.P. (2004). Metagenes and molecular pattern discovery using matrix factorization. Proceedings of the National Academy of Sciences of the United States of America 101, 4164–4169.

Cancer Genome Atlas Research Network, Weinstein, J.N., Collisson, E.A., Mills, G.B., Shaw, K.R.M., Ozenberger, B.A., Ellrott, K., Shmulevich, I., Sander, C., and Stuart, J.M. (2013). The Cancer Genome Atlas Pan-Cancer analysis project. Nature Genetics 45, 1113–1120.

Cerami, E.G., Gross, B.E., Demir, E., Rodchenkov, I., Babur, Ö., Anwar, N., Schultz, N., Bader, G.D., and Sander, C. (2011). Pathway Commons, a web resource for biological pathway data. Nucleic Acids Res 39, D685–D690.

Ciriello, G., Miller, M.L., Aksoy, B.A., Senbabaoglu, Y., Schultz, N., and Sander, C. (2013). Emerging landscape of oncogenic signatures across human cancers. Nature Genetics 45, 1127–1133.

Croft, D., Mundo, A.F., Haw, R., Milacic, M., Weiser, J., Wu, G., Caudy, M., Garapati, P., Gillespie, M., Kamdar, M.R., et al. (2014). The Reactome pathway knowledgebase. Nucleic Acids Res 42, D472–D477.

Dai, M., Wang, P., Boyd, A.D., Kostov, G., Athey, B., Jones, E.G., Bunney, W.E., Myers, R.M., Speed, T.P., Akil, H., et al. (2005). Evolving gene/transcript definitions significantly alter the interpretation of GeneChip data. Nucleic Acids Res 33, e175.

Garnett, M.J., Edelman, E.J., Heidorn, S.J., Greenman, C.D., Dastur, A., Lau, K.W., Greninger, P., Thompson, I.R., Luo, X., Soares, J., et al. (2012). Systematic identification of genomic markers of drug sensitivity in cancer cells. Nature 483, 570–575.

Gatza, M.L., Lucas, J.E., Barry, W.T., Kim, J.W., Wang, Q., Crawford, M.D., Datto, M.B., Kelley, M., Mathey-Prevot, B., Potti, A., et al. (2010). A pathway-based classification of human breast cancer. Proceedings of the National Academy of Sciences 107, 6994–6999.

Gatza, M.L., Silva, G.O., Parker, J.S., Fan, C., and Perou, C.M. (2014). An integrated genomics approach identifies drivers of proliferation in luminal-subtype human breast cancer. Nature Genetics 46, 1051–1059.

Gonzalez-Perez, A., and Lopez-Bigas, N. (2012). Functional impact bias reveals cancer drivers. Nucleic Acids Res 40, e169.

Irizarry, R.A., Hobbs, B., Collin, F., Beazer-Barclay, Y.D., Antonellis, K.J., Scherf, U., and Speed, T.P. (2003). Exploration, normalization, and summaries of high density oligonucleotide array probe level data. Biostatistics 4, 249–264.

Knijnenburg, T., Klau, G., Iorio, F., Garnett, M., McDermott, U., Shmulevich I., Wessels L. (2016). Logic models to predict continuous outputs based on binary inputs with an application to personalized cancer therapy. bioRxiv doi: http://dx.doi.org/10.1101/036970.

Knijnenburg, T.A., Lin, J., Rovira, H., Boyle, J., and Shmulevich, I. (2011). EPEPT: a web service for enhanced P-value estimation in permutation tests. BMC Bioinformatics 12, 411.

Knijnenburg, T.A., Wessels, L.F.A., Reinders, M.J.T., and Shmulevich, I. (2009). Fewer permutations, more accurate P-values. Bioinformatics 25, i161–i168.

Lawrence, M.S., Stojanov, P., Polak, P., Kryukov, G.V., Cibulskis, K., Sivachenko, A., Carter, S.L., Stewart, C., Mermel, C.H., Roberts, S.A., et al. (2013). Mutational heterogeneity in cancer and the search for new cancer-associated genes. Nature 499, 214–218.

Newman, M.E.J. (2004). Fast algorithm for detecting community structure in networks. Phys Rev E Stat Nonlin Soft Matter Phys 69, 066133.

Parikh, J.R., Klinger, B., Xia, Y., Marto, J.A., and Bluthgen, N. (2010). Discovering causal signaling pathways through gene-expression patterns. Nucleic Acids Res 38, W109–W117.

Pinheiro, J., Bates, D., DebRoy, S., and Sarkar, D. (2007). Linear and nonlinear mixed effects models. R Package Version.

Rubio-Perez, C., Tamborero, D., Schroeder, M.P., Antolín, A.A., Deu-Pons, J., Perez-Llamas, C., Mestres, J., Gonzalez-Perez, A., and Lopez-Bigas, N. (2015). In silico prescription of anticancer drugs to cohorts of 28 tumor types reveals targeting opportunities. Cancer Cell 27, 382–396.

Seashore-Ludlow, B., Rees, M.G., Cheah, J.H., Cokol, M., Price, E.V., Coletti, M.E., Jones, V., Bodycombe, N.E., Soule, C.K., Gould, J., et al. (2015). Harnessing Connectivity in a Large-Scale Small-Molecule Sensitivity Dataset. Cancer Discov 5, 1210–1223.

Storey, J.D., and Tibshirani, R. (2003). Statistical significance for genomewide studies. Proceedings of the National Academy of Sciences of the United States of America 100, 9440–9445.

Tamborero, D., Gonzalez-Perez, A., and Lopez-Bigas, N. (2013a). OncodriveCLUST: exploiting the positional clustering of somatic mutations to identify cancer genes. Bioinformatics 29, 2238–2244.

Tamborero, D., Gonzalez-Perez, A., Perez-Llamas, C., Deu-Pons, J., Kandoth, C., Reimand, J., Lawrence, M.S., Getz, G., Bader, G.D., Ding, L., et al. (2013b). Comprehensive identification of mutational cancer driver genes across 12 tumor types. Sci Rep 3, 2650.

Turcan, S., Rohle, D., Goenka, A., Walsh, L.A., Fang, F., Yilmaz, E., Campos, C., Fabius, A.W.M., Lu, C., Ward, P.S., et al. (2012). IDH1 mutation is sufficient to establish the glioma hypermethylator phenotype. Nature 483, 479–483.

Van der Maaten, L., and Hinton, G. (2008). Visualizing data using t-SNE. Journal of Machine Learning Journal of Machine Learning Research 9, 2579-2605

van Dyk, E., Reinders, M.J.T., and Wessels, L.F.A. (2013). A scale-space method for detecting recurrent DNA copy number changes with analytical false discovery rate control. Nucleic Acids Res 41, e100.

Vis, D.J., Bombardelli, L., Lightfoot, H., Iorio, F., Garnett, M.J., Wessels, L.F.A. Multilevel models improve precision and speed of IC50 estimates. Pharmacogenomics, 17 (7) [in press] doi: 10.2217/pgs.16.15.

Wong, C.C., Martincorena, I., Rust, A.G., Rashid, M., Alifrangis, C., Alexandrov, L.B., Tiffen, J.C., Kober, C., Chronic Myeloid Disorders Working Group of the International Cancer Genome Consortium, Green, A.R., et al. (2014). Inactivating CUX1 mutations promote tumorigenesis. Nature Genetics 46, 33–38.

**Supplemental Data S1**

**A. Cancer functional events covered by the cell lines**

The median percentage of cancer-type specific (C-S) driver genes (CGs) found mutated in at least one cell line sample across individual cancer types was equal to 80% (min = 0% for CLL and max = 100% for LUAD, BRCA, GBM, OV, NB, ALL, LIHC) when considering CGs mutated in at least 5% of the tumor samples from the cancer type under consideration (Figure 2A). The median value was 22% (min = 3% for CLL, max = 83% for ALL) (Figure S2B) when considering all the CGs.

When considering C-S RACSs amplified in at least 5% of the primary tumor samples from the corresponding cancer type, the median coverage across cancer types was equal to 88% (Figure 2A) (min = 0% for ACC, THCA, LAML, KIRC and MESO, max = 100% for CESC, DLBC, ESCA, BRCA, LUAD, and PAAD). The median value was equal to 80% (min = 0% for ACC, LAML, KIRC and MESO, max = 100% for DLBC, BRCA, ESCA, and PAAD), when considering all the C-S RACSs (Figure S2B).

Similarly, the median percentage of coverage for C-S recurrently deleted RACSs (i.e. deleted in at least 5% of the tumor samples from the corresponding cancer type), was equal to 78% (Figure 2A) (min = 0% for ACC, max = 100% for PAAD, LUAD and MESO). The median value dropped to 70% when considering all the deleted RACSs (Figure S2B) (min = 0% for ACC, and max = 100% for PAAD, LUAD, and MESO, in both the cases).

All the C-S sets of informative CpG islands (iCpG) with a finite discretization threshold (computed as detailed in the SEP) were observed as hypermethylated in at least one cell line sample from the cancer type under consideration (according to the same threshold), but for LGG (83%) and PRAD (90%).

In many of the cases where cancer functional event (CFE) coverage in the cell line panel was modest, a CFE was either altered at a very low frequency in patient tumors of the relevant cancer type, or few cell lines of the relevant cancer type were available. For example, CGs absent in the corresponding cell lines had a median frequency of 1.30% in primary tumor samples (Table S2K) (min median = 0.35% for BRCA specific CGs missing in BRCA cell lines, max median = 11% for for DLBC specific CGs missing in DLBC cell lines) (Table S2K). Similarly, the majority of C-S RACSs absent in cell lines had a low alteration frequency in the primary tumors (median average alteration frequency across cancer types = 4.3%, min = 0.04% for LUAD, max = 7.11% for PRAD) (Figure S2B and Table S2K). Furthermore, many CGs absent in the cell lines are associated with less strong evidence for positive selection (56% classed as Level C, 23% classed as Level B), with the median composition of the three confidence levels across cancer types = 54% C, 24% B, 23% A (Figure S2B and Table S2K).

**B. Classes of cancer functional event and enrichment analysis**

By applying the method described in (Ciriello et al., 2013) to our integrated dataset of tumor and cell lines, as described in the experimental procedures and the extended experimental procedures (SEP) 11, we identified 4 classes of Cancer Functional Events: M, CD, CA, and H.

Class M is enriched for Cancer Genes (CGs) mutations, classes CD and CA for recurrently altered chromosomal segments (RACSs) copy number alterations, and class H for hypermethylated informative CpG islands (iCpGs). All these feature types are defined in the experimental procedures and the SEP3, 5, and 7.

The M class represented 34% of samples (36% of the primary tumors and 28% of the cell lines). It was enriched for CG mutations (Fisher exact test (FET) p-value = $5.41 \times 10^{-130}$) and was dominated by cell lines and tumors from THCA (95% of the tumors and 63% of the cell lines), SKCM (73% of the tumors and 73% of the cell lines), and COAD/READ (80% of the tumors and 86% of the cell lines) (Figure 3C, Table S3D, and Table S3E).

When looking at enrichments of individual CFEs in class M (as detailed in the SEP12), we found that the top enriched 10 mutations involved: *BRAF*, *APC*, *PTEN*, *ANK3*, *KRAS*, *CTNNB1*, *NF1*, *ARID1A*, *ATM* and *MLL3* (Table S3F). Class M included, as individually enriched, deleted segments containing *ZNF3*, and *ARFGAP3* (Figure S3 and Table S3F) and hypermethylated CpG islands in the promoter regions of *DUSP22* and *ARL17A*.

Class CD represented 15% of the samples (15% of the tumors and 13% of the cell lines) and was enriched for RACSs copy number alterations (CNAs) (FET p-value = $5.12 \times 10^{-36}$), particularly for RACSs deletions (FET p-value = $2.04 \times 10^{-15}$). It was dominated by, consistently across cell lines and tumors, the LAML (68% of the tumors and 79% of the cell lines), KIRC (38% of the tumors and 53% of the cell lines), and GBM (70% of the tumors and 17% of the cell lines) samples, and partially covered PRAD, SKCM, and LUAD samples, consistently across cell lines and tumors (Figure 3C, Table S3D, and Table S3E).

The most significantly enriched RACS alterations in the CD class involved deletions of segments containing *RET*, *ANK3*, *PTEN*, *CUL2*, *ABL1*, *JAK1* and *MAP3K4*. Class CD included 5 individually enriched mutations involving, *NPM1*, *PBRM1*, *IDH2*, *IDH1*, and *U2AF1*. Thus making the AML samples highly representative of this class and its underlying signature. A small number of iCpG hypermethylations were enriched as well, for example in the promoters of *FAM115A* and *PIK3R1* (Figure S3 and Table S3F).

Class CA was enriched for copy number alteration CFEs (FET p-value = $2.95 \times 10^{-12}$) and represents, consistently across cell lines and tumors, most of the BRCA (99% of the tumors and 100% of the cell lines), HNSC (95% of the tumors and 100% of the cell lines), LUAD (51% of the tumors and 89% of the cell lines) and LUSC (61% of the tumors and 80% of the cell lines) (Figure 3C, Table S3D, and Table S3E).

Class CA was prominently enriched for amplified RACSs (FET p-value = $2.38 \times 10^{-16}$, Table S3H). Among these we found amplifications of regions containing *MYC* (the most significant amplified gene), *RAD21*, *PABPC1*, *FGFR1*, *PIK3CB*, *ERBB2*, and *KRAS*, and deletions of segments including *CDKN2A* and *TP53*; consistently with this, enriched hypermethylated iCpGs were found in the promoters of *CDKN2A* and *CDKL2* (among the others). 7 mutations were enriched at the individual level and included alterations in *TP53*, *PIK3CA*, *NOTCH1*, *GATA3*, *CASP8* and *NFE2L2* (Figure S3 and Table S3F).

Finally, the H class was enriched for hypermethylated iCpGs (p-value = $2.19 \times 10^{-48}$) and was exclusively composed by LGG samples (80% of the tumors and 59% of the cell lines), consistent with the hypermethylation phenotype of glioma (Turcan et al., 2012).

Using this classification of tumor samples, we observed a good concordance between the contributions of each our 4 classes to the considered 15 cancer types and that reported in the previous study: 70% of M-to-M and C-to-CA/CD matching classifications, including BLCA and LUAD partially belonging to M and C in both cases with ratios close to 50% (Ciriello et al., 2013). Additionally, in our case, a clear partition of the C class into two subclasses (CD and CA, respectively enriched for copy number deletions and amplifications) emerged unsupervised from the data and correctly covered samples from relevant cancer types. Finally, due to the inclusion of a larger epigenomic dataset, we were able to detect an additional class (enriched for hypermethylated CpG islands) correctly classifying most of the LGG samples to this.

Finally, we observed concordance using these 4 classes when applied to primary tumors and cell lines of the same tissue type (the predominant CFE type, i.e. mutations, copy number alterations and hypermethylation) is consistently detected for 100% of the cancer types. For 12 out of 15 cancer types, 80% are consistently classified even when distinguishing between CA (associated to amplifications) and CD (associated to deletions). Collectively, these data demonstrate that in addition to concordance between primary tumor samples and matched cell lines at the level of CFE frequencies, this is also evident when looking at more global signatures of functional events (Table S3G).

**C. Validation of the LOBICO models on an independent dataset**

LOBICO was successfully run for 83 out of the 86 compounds in common between our study and the CTRP ($2^{nd}$ version, (Seashore-Ludlow et al., 2015)) (see Supporting Experimental Procedure, paragraph 24). For the remaining 3 compounds, fewer than 5 cell lines were sensitive according to the automated procedure to binarize the $IC_{50}$s, and thus LOBICO analysis was not performed. Table S5G provides details of all models. For 80 of the 83 models the groups of predictive and sensitive cell lines contained at least 5 cell lines. The p-values for the t-tests on the GDSC $IC_{50}$s and CTRP AUCs for the 81 models were highly correlated (Figure S5E) (Pearson correlation: 0.96, p-value = $2.0 \times 10^{-43}$). When the 10 rescreens are removed the correlation is similar (0.95), but with a higher, yet still very significant p-value of $4.8 \times 10^{-37}$. Selection of significant t-test p-values using a Bonferroni corrected p-value of 0.01 as a cut-off showed significant overlap between GDSC and CTRP (Fisher exact test, p = $1.7 \times 10^{-6}$). This value drops to p = $1.5 \times 10^{-5}$ in case the rescreens were not considered.

We applied a further filter to focus on interesting results. Particularly, we considered the 44 'predictive' LOBICO models. These models have a p-value < 0.05 and FDR < 5% according to the permutation tests (See SEP Section 20). Note that these are different from the t-test p-values. Of these 44 models, 7 were single-predictor models and 37 were multi-predictor models according to CV. For 43 of these 44 models the groups of predictive and sensitive cell lines contained at least 5 cell lines. The t-test p-values for these 43 models for GDSC and CTRP are depicted in Figure S5F. Also from this figure, there is clearly a large agreement between the two studies; LOBICO models inferred on GDSC data show separation between drug response measurements both for the GDSC $IC_{50}$s as well as the CTRP AUCs. Specifically, 9 out of the 10 best GDSC models were also significant in CTRP. (Here, we used a P-value cutoff of a 0.023, i.e. 1/43, corresponding to a FWER of 1 and a corresponding FDR smaller than 1%, to decide upon significance). In total, there were 39 models that were statistically significant for the GDSC data, 17 (44%) of which also showed significance in the CTRP dataset. We have created violin plots with the underlying drug response data for these models (available at: http://www.cancerrxgene.org/gdsc1000/Logic_models.html).
